# Solid-phase fluorescent BODIPY–peptide synthesis *via in situ* dipyrrin construction†

**DOI:** 10.1039/d0sc04849f

**Published:** 2020-09-24

**Authors:** Yue Wu, Wing-Sze Tam, Ho-Fai Chau, Simranjeet Kaur, Waygen Thor, Wei Shen Aik, Wai-Lun Chan, Markus Zweckstetter, Ka-Leung Wong

**Affiliations:** Department of Chemistry, Hong Kong Baptist University Kowloon Hong Kong SAR China kulicechan@hkbu.edu.hk klwong@hkbu.edu.hk wai-lun.chan@mpibpc.mpg.de; Department for NMR-based Structural Biology, Max Planck Institute for Biophysical Chemistry Am Fassberg 11 37077 Göttingen Germany mazw@nmr.mpibpc.mpg.de; German Center for Neurodegenerative Diseases (DZNE) Von-Siebold-Str. 3a 37075 Göttingen Germany markus.zweckstetter@dzne.de

## Abstract

Traditional fluorescent peptide chemical syntheses hinge on the use of limited fluorescent/dye-taggable unnatural amino acids and entail multiple costly purifications. Here we describe a facile and efficient protocol for *in situ* construction of dipyrrins on the N-terminus with 20 natural and five unnatural amino acids and the lysine's side chain of selected peptides/peptide drugs through Fmoc-based solid-phase peptide synthesis. The new strategy enables the direct formation of boron–dipyrromethene (BODIPY)–peptide conjugates from simple aldehyde and pyrrole derivatives without pre-functionalization, and only requires a single-time chromatographic purification at the final stage. As a model study, synthesized EBNA1-targeting **BODIPY1–Pep4** demonstrates intact selectivity *in vitro*, responsive fluorescence enhancement, and higher light cytotoxicity due to the photo-generation of cytotoxic singlet oxygen. This work offers a novel practical synthetic platform for fluorescent peptides for multifaceted biomedical applications.

## Abstract 2

Solid-phase fluorescent BODIPY–peptide synthesis *via in situ* dipyrrin construction offers an efficient fluorescent peptide synthetic platform for multifaceted biomedical applications.

## Graphical abstract

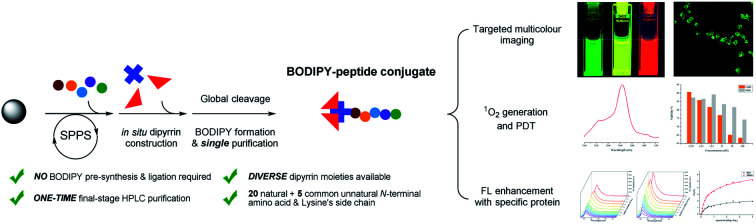

## Introduction

Fluorescent peptide/peptidomimetic-based targeting and visualization of the subcellular localization,^1,2^ and probing and modulation of intra/inter-biomolecular interactions,^3,4^ of the protein target of interest provides a versatile platform, either *in vitro*/*vivo* or in assays,^5–7^ for fundamental biochemistry/chemical biology research,^8–10^ as well as drug discovery and development.^11–14^ With the growing significance and recent successes in peptide (and peptide-containing) therapeutics,^15–20^ by virtue of their (i) scalable modular synthetic accessibility and tunability, (ii) target-specific selectivity, potency and efficacy, (iii) good biocompatibility, low systemic toxicity and easy clearance, there is a great demand for more general and flexible synthetic methodologies and ligation protocols to make peptides fluorescent, upon functional *de novo* design, for in-depth biomedical investigations.

**Fig. 1:**
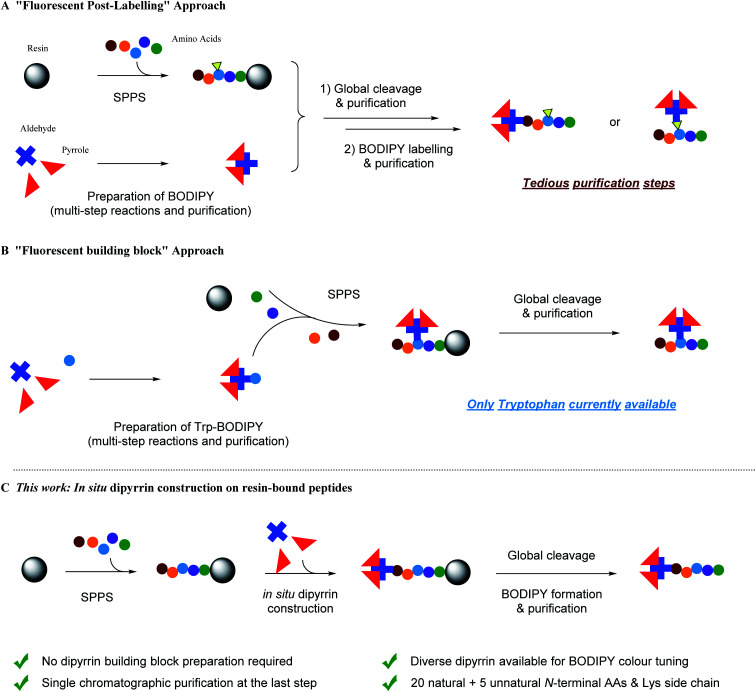
Schematic representation of strategies for BODIPY-based fluorescent peptide synthesis. (A) Post-SPPS ligation of fluorescent BODIPY dyes on peptides requiring multistep transformations and purifications. (B) Use of BODIPY-derived fluorescent unnatural amino acid/pre-functionalized building blocks during SPPS. (C) This work: *in situ* dipyrrin construction during SPPS and then boron complexation to form BODIPY dyes with a single chromatographic purification step.

To date, despite the development of automated flow-based approaches,^21,22^ solid-phase peptide synthesis (SPPS) remains the most common method for peptide preparation at both academic and industrial research settings.^23–27^ In SPPS, amino acids with the protected N-terminus (as well as the protected side chain if necessary) are used as building blocks: upon the covalent attachment onto insoluble resin beads and the iterative amidation and N-Fmoc deprotection, tailor-designed peptides of known sequence can be prepared in very good to excellent yields. Meanwhile, all the excess reactants and unbound side-products can be removed by simple washing after each single step, with the only purification step being required until the final cleavage of the peptide from the resin. However, when it comes to synthetic fluorescent peptides, *i.e.* fused with fluorescent proteins (FPs) by molecular biology and site-specific protein labelling protocols,^28–30^ or labelled/modified with small-molecule fluorophores (*e.g.* FITC, TRITC, AMCA, 5/6-FAM, Cy3/5, Lucifer Yellow, *etc.*) at either the N-terminus or at the internal lysines/cysteines/unnatural amino acids' linkable/clickable side chains with straightforward functional group coupling reactions and bioorthogonal ligations,^31–38^ multiple transformation steps followed by tedious and costly purifications are required. In addition, relatively large fusion FPs and steric fluorophores can alter the physio–chemical properties of short peptides and adversely interfere with their interactions with targets *in vitro*/*vivo*.^39^ Therefore, employing an optimal spacer/linker is technically a key routine solution for this “fluorescent post-labelling” approach (Fig. 1A).

To circumvent the above-mentioned limitations, the “fluorescent building block” approach has emerged as a highly promising alternative for fluorescent peptide production (Fig. 1B).^40^ For instance, Schultz *et al.* pioneered the use of genetic encoding to introduce fluorescent unnatural amino acids into proteins at specific sites.^41,42^ In addition, Imperiali *et al.* developed an SPPS-based approach for incorporation of fluorescent unnatural amino acids into peptides by design.^43–45^ Since then, a kaleidoscope of novel organic fluorophore-based fluorescent unnatural amino acids with different photophysical properties and structures have been developed.^40^ However, interdisciplinary expertise in synthetic biology and chemistry, experience in careful and judicious peptide design, and sufficient research budget are prerequisites for these approaches.

Over the past few decades, fluorinated boron–dipyrromethene (BODIPY) and its derivatives^46^ have lent themselves to multifarious functional fluorophore scaffolds for diverse biomedical applications,^47^*e.g.* fluorescence sensing and imaging (*via* the Förster resonance energy transfer (FRET) or photoinduced electron transfer (PeT) mechanisms), positron emission tomography imaging, photodynamic therapy, as well as *in vitro* and in *vivo* assays, due to their high synthetic availability and cost-effectiveness, tunable photophysical properties and neutral total charge for better cell permeability. BODIPY–peptide conjugates are therefore widely used as dual targeting–imaging tools in life sciences,^7,48–54^ although a “fluorescent post-labelling” strategy has to be used since BODIPY derivatives are generally vulnerable to strong acid and usually not so adaptable to SPPS conditions.^55–57^ To this end, Vendrell *et al.* documented the first “fluorescent building block” approach by developing a fluorogenic Trp–BODIPY amino acid in a spacer-free manner (*i.e.* direct carbon–carbon bond linkage between a tryptophan's indole moiety at the C2-position and a BODIPY dye by palladium-catalyzed C_sp2_–C_sp2_ Heck reaction).^58,59^ Both linear and cyclic peptides can be prepared.^60–62^ In addition, Ackermann *et al.* disclosed a palladium-catalyzed bioorthogonal late-stage C_sp3_–H activation strategy to append BODIPY dyes to the alanine and phenylalanine residues within a peptide.^63^

Because tryptophan might not be present in the peptide sequence of interest, while phenylalanine and alanine are often involved in functional interactions and conformational controls of the peptide's secondary/tertiary structures, *i.e.* should best not be perturbed, complementary and more universal methods are required to create BODIPY–peptide conjugates. For the BODIPY precursor, there are two common ways to synthesize dipyrrin derivatives:^64^ first, Method A, condensation between one aldehyde and two pyrroles, followed by oxidation, which yields symmetrical dipyrrins; second, Method B, reaction of an α-ketopyrrole/α-formylpyrrole with another pyrrole, giving non-symmetrical dipyrrins. For both methods, a lot of impurities, including excess pyrroles, intra-pyrrole crossing products, and the oxidant and its derivatives, are mixed with desired product, which renders the purification step a laborious and tedious task. Inspired by the advantage of SPPS, we hypothesize that SPPS and dipyrrin synthesis, in this regard, can be perfectly integrated (Fig. 1C).

Herein, we describe a new facile and efficient protocol for *in situ* dipyrrin construction on the N-terminus and the side chains of peptides through Fmoc-based solid-phase peptide synthesis (SPPS), where simple aldehyde and pyrrole derivatives can be directly utilized as building blocks without pre-functionalization in SPPS, and only one final-stage chromatographic purification step is required. In connection to our on-going research program to develop peptide-based EBNA1 inhibitors against Epstein–Barr virus (EBV)-associated maligenancies,^65–67^ we constructed dipyrrin moieties on our own library of established EBNA1-targeting peptide **Pep4** and synthesized **BODIPY–Pep4**, which manifests no erosion of biotargeting performance *in vitro* but enhanced light cytotoxicity over dark cytotoxicity. This can be correlated to the photodynamic therapy (PDT) effect of BODIPY by photoinduced cytotoxic singlet oxygen (^1^O_2_) generation. Taken together, this study offers a new pragmatic SPPS methodology for fluorescent peptide production and facilitates a cornucopia of biomedical applications.

## Results and discussion

**Fig. 2:**
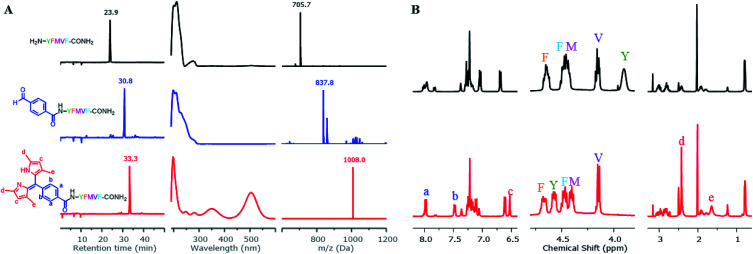
Direct construction of dipyrrin on resin–bound **Pep1**. (A) The crude samples (resin) were taken and cleaved, with the resulting solutions being monitored by HPLC and ESI-MS. From top to bottom: unconjugated peptide (black), aldehyde–peptide conjugate (blue), dipyrrin–peptide conjugate **DP1–Pep1** (red); from left to right: their HPLC chromatographs, UV-Vis spectra corresponding to the peak on HPLC, and ESI-MS spectra. (B) Comparisons of ^1^H-NMR spectra of **Pep1** and **DP1–Pep1**: four protons from the aldehyde building block (a and b) and two protons from the pyrrole (c) were found in the aromatic region; the α-H of tyrosine (Y, near the N-terminal) shifts largely after conjugation; 12 protons from the four methyl groups of dipyrrin (d and e) were found at 2.4 and 1.7 ppm.

To construct dipyrrin on the N-terminus of resin–bound **Pep1** (H_2_N–YFMVF–COOH), which was previously used as an EBNA1-targeting peptide,^65–67^ the benzaldehyde moiety was installed on the N-terminus through routine SPPS, followed by condensation with 2,4-dimethylpyrrole under the catalysis of BF_3_·OEt_2_ on the resin. Subsequently, 2,3-dichloro-5,6-dicyano-1,4-benzoquinone (DDQ) was added to oxidize the newly formed dipyrrolomethane into dipyrrin *in situ*: the resin turned red after this step indicating the formation of dipyrrin. After global cleavage, the dipyrrin–peptide conjugate **DP1–Pep1** was obtained with 51% yield, which is comparable to the yield of the unconjugated peptide (**Pep1**, 60%). The reaction was monitored by HLPC, ESI-MS and ^1^H-NMR (Fig. 2).

**Fig. 3:**
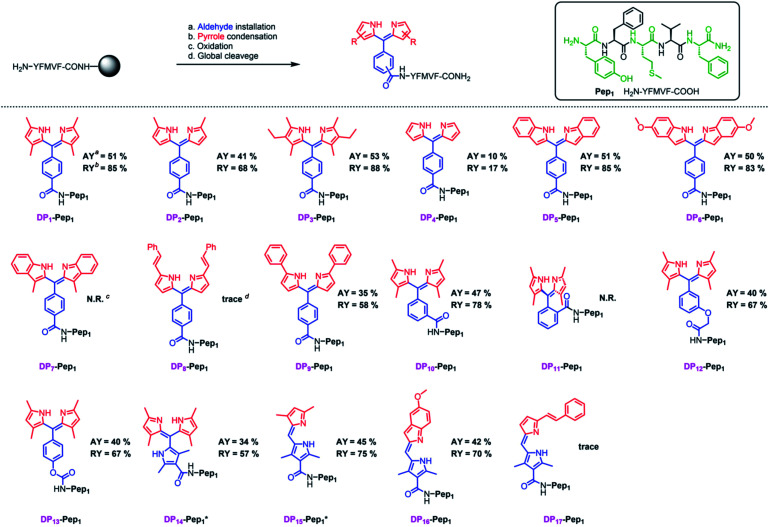
*In situ* construction of dipyrrin derivatives on the peptide YFMVF. Condition: (a) aldehyde-containing carboxylic acid, PyBOP, DIPEA, DMF, 3 h. (b) Pyrrole derivatives, BF_3_·OEt_2_, r.t., DMF, overnight; (c) DDQ, 1 h, DCM; (d) TFA/TIPS/H_2_O, v/v/v, 95/2.5/2.5, r.t., 2 h. ^*a*^Absolute Isolated yield compared with the substitution value of the resin–bound peptide. ^*b*^Relative yield compared with the isolated yield of the unconjugated peptide.^*c*,*d*^ No/Very few desired products were detected by LC-MS.

We next explored the substrate diversity of various aldehydes and pyrroles under the above-mentioned procedure to synthesize a series of dipyrrin conjugates of **Pep1** (Fig. 3). Various simple pyrrole derivatives and indole derivatives were tried, and most of them yielded corresponding symmetrical dipyrrin conjugates in good both absolute and relative yields. The failures of **DP7** and **DP11** suggest that the reaction can be hindered by the steric effect, while the trace amount of **DP8/17** hints towards the sensitivity of the conjugated vinyl group towards the conditions. Since functionalized α-ketopyrroles/α-formylpyrroles (*e.g.* –COOH group containing) have very few commercial sources and are hard to synthesize as building blocks, 5-formyl-2,4-dimethyl-3-pyrrolecarboxylic acid (FDMPA) attracted our attention for its commercial availability and low cost (below 10 USD per g). FDMPA was able to act as an aldehyde analogue to prepare **DP14**. For the unsymmetrical cases, we adopted the Method B. Treated by POCl_3_ with other pyrrole derivatives, **DP15** and **DP16** were obtained in good yields.

**Fig. 4:**
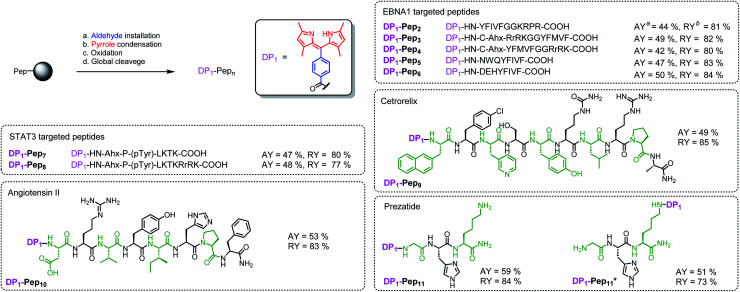
*In situ* construction of **DP1** on different peptides. ^*a*^Absolute isolated yield compared with substitution value of the resin–bound peptide. ^*b*^Relative yield compared with the isolated yield of the unconjugated peptide.

We then verified the scope of this methodology for peptides with different amino acid compositions (Fig. 4). 4-Formylbenzoic acid and 2,4-dimethylpyrrole were used to construct 1,3,7,9-tetramethyldipyrrin (**DP1**) for a series of peptides that cover all 20 natural amino acids as well as five commonly used unnatural amino acids. All were obtained with comparable yields as the corresponding unconjugated peptides. The synthesized peptides can be used in a variety of studies, such as EBNA1-targeting peptides (**Pep1–Pep6**), STAT3-targeting peptides (**Pep7** and **Pep8**), as well as some FDA-approved peptide drugs including cetrorelix (**Pep9**, as GnRH antagonists for treating prostate cancer, endometriosis, uterine fibroids), angiotensin II (**Pep10**, used in treatment of sepsis, septic shock, diabetes mellitus, and acute renal failure) and prezatide (**Pep11**, a copper chelator with potential applications in wound healing and other different functions). In addition to N-terminal conjugation, the dipyrrin moiety can also be installed on the side chain of the lysine residue of **Pep11** (the lysine with a removable protecting group on the amine group on the side chain), which substantiates that our procedure can be applied “generally and flexibly” on side chain modifications as well.

**Fig. 5:**
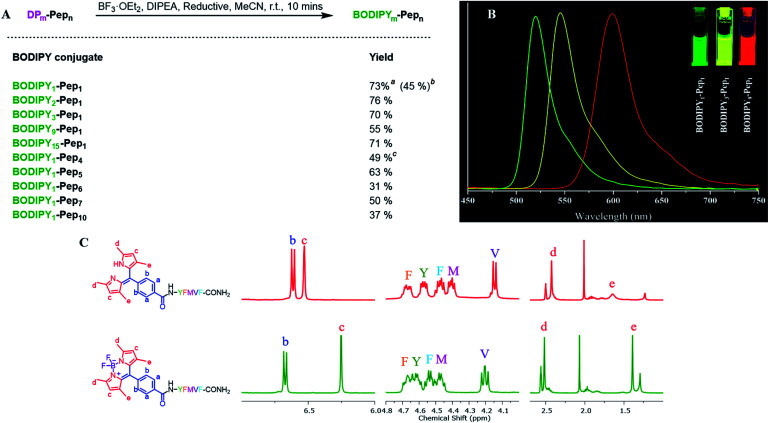
(A) Boron complexation for dipyrrin–peptide conjugates. ^*a*^Purified dipyrrin conjugate was used; isolated yield compared with the dipyrrin conjugate. ^*b*^Crude dipyrrin conjugate was used; isolated yield compared with the substitution value of the resin–bound peptide. ^*c*^EDT was used as the reductant. (B) The fluorescence spectra of 10 μM BODIPY–peptide conjugates (derived from **DP1**, **DP3** and **DP9** respectively) in DMSO under 365 nm UV light excitation. (C) Comparison of ^1^H-NMR spectra of **DP1–Pep1** and **BODIPY1–Pep1**. The chemical shifts of both pyrrole–H (c) and methyl–H (e) are shifted towards the high field.

Next, we investigated SPPS boron complexation, which is a long-standing challenge for dipyrrin–peptide conjugates to deliver BODIPY–peptide conjugates (Fig. 5A). Traditionally, this complexation step is conducted in less-polar solvents. However, peptides often suffer from poor solubility in these solvents, while polar solvents resulted in no complexation. Upon systematic solvent screening, we found acetonitrile (ACN), which displays moderate solubility for peptides, to be optimal: both the purified dipyrrin–peptide conjugates and the crude product from SPPS were converted in ACN into the corresponding BODIPY conjugates within 10 minutes with acceptable yields. Notably, bulky base DIPEA should be used and the products should be separated from the reacting mixture rapidly, in order to avoid racemization. ^1^H-NMR spectra of **DP1–Pep1** and **BODIPY1–Pep1** are shown in Fig. 5C and S8–S11.† In addition, BODIPY-conjugates with different colours of fluorescence were prepared (Fig. 5B), further illustrating the possibility to achieve fast fluorescence labelling of *de novo* peptides with diverse BODIPY dyes for bioimaging and biosensing. It is axiomatic that the photophysical properties of BODIPY dyes are largely influenced by the extent of electron delocalization around the boron centre (*e.g.* most BODIPY dyes emitting around 510 nm in green colour, like **BODIPY1–Pep1**) by peripherical substitutions on the dipyrrin to (i) extend the π-conjugation system with vinyl/ynyl/aromatic groups (*e.g.* highly red-shifted **BODIPY9–Pep1** to red colour) and (ii) subtly fine-tune it with either electron-donating (*e.g.* aliphatic groups in **BODIPY3–Pep1** for slightly shifting to yellow colour) or electron-withdrawing groups (*e.g.* halogens).^46,68^

**Fig. 6:**
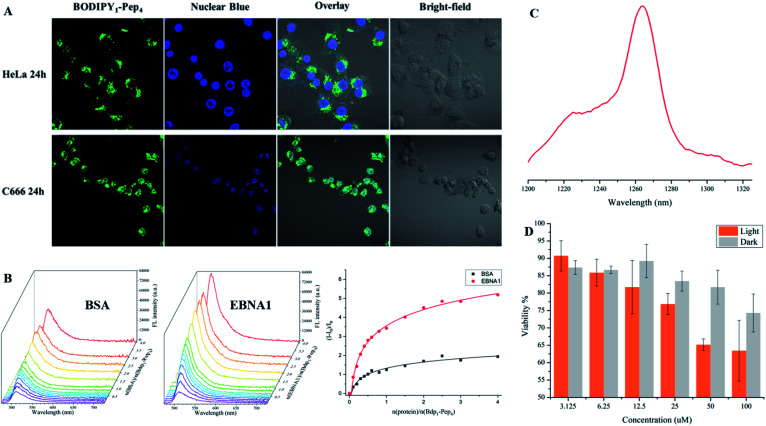
Imaging of fluorescent peptides. (A) Confocal imaging of **BODIPY1–Pep4** in HeLa and C666 cell lines. The green fluorescence signal was detected in the nucleus of C666 (EBNA+) but not in the nucleus of HeLa (EBNA−) cells. (B) Fluorescence titration of BSA (left) and EBNA1 (middle) proteins, both at 1.2 μM, with **BODIPY1–Pep4**. A nearly 5-times enhancement over the course of the titration with EBNA1 was observed (right). (C) Singlet oxygen signal illustrating the potential of **BODIPY1–Pep4** as a PDT agent. (D). Comparison of dark/light cytotoxicity of **BODIPY1–Pep4** towards C666 cell line. Light toxicity was significantly higher than the dark toxicity. Error bars represent the standard derivation of the mean.

To support the practical use of the BODIPY–peptide conjugates synthesized by our protocol, we performed confocal imaging for **BODIPY1–Pep4** (where **Pep4** had been designed as a nucleus-penetrating EBNA1-specific peptide in our previous research)^65–67^ with both a HeLa cell line (EBNA1^−^) and the C666 cell line (EBNA1^+^) (Fig. 6A and S6†). As expected, the signal of BODIPY accumulated relatively fast in the nucleus of C666 cells where the EBNA1 proteins are located, while the uptake of BODIPY into the nucleus of HeLa cells was slow. The confocal imaging experiments confirmed selective EBNA1-targeting performance of **BODIPY1–Pep4***in vitro* despite the use of another chromophore. In addition, **BODIPY1–Pep4** demonstrated an almost 5-fold binding-responsive fluorescence enhancement (Fig. 6B, S1 and S2†), as well as a dark/light cytotoxicity difference due to the photo-generation of cytotoxic singlet oxygen (^1^O_2_) from the BODIPY moiety (Fig. 6C and D).

## Conclusion

We have developed an efficient and convenient methodology to conjugate peptides with dipyrrin moieties during SPPS that can be further derived into highly emissive bioactive BODIPY–peptide conjugates for multicolour imaging. Various dipyrrin derivatives can be constructed on either the N-terminus or the side chain of peptides in both symmetrical and unsymmetrical ways and the substrate scope comprises all 20 natural and five unnatural amino acids with good yield. The workload and cost for synthesizing dipyrrin/BODIPY–peptide conjugates is greatly reduced by this protocol, which therefore holds tremendous promise for expediting the screening of peptide-based fluorescent probes, as well as for the development of high-throughput (HTS) fluorescence screening platforms and the creation of novel metal–peptide nano-frameworks. The BODIPY-labelled EBNA1-specific peptide (**BODIPY1–Pep4**) obtained from this methodology exhibited excellent *in vitro* performance and can serve as a potential photosensitizer for PDT. This work furnishes a new pragmatic alternative SPPS methodology for fluorescent peptide production that can leverage and impact multifaceted biomedical applications.

## Conflicts of interest

The authors declare no conflicts of interest.

## Supplementary Material

SC-011-D0SC04849F-s001
