# Supplementary material for: Solid-phase fluorescent BODIPY–peptide synthesis via in situ dipyrrin construction
Source: Chem Sci. 2020 Sep 24;11(41):11266–73. doi: 10.1039/d0sc04849f (PMC8162834; doi:10.1039/d0sc04849f)
Supplement: SC-011-D0SC04849F-s001 [file SC-011-D0SC04849F-s001.pdf]

Electronic Supplementary Material (ESI) for Chemical Science.
This journal is © The Royal Society of Chemistry 2020

Content

**Titles**	**Pages**
Emission spectra of $BODIPY_1$-Pep_4 in mixture solvent of water and DMSO	S2
Photophysical properties of BODIPY-peptide conjugates	S3
Distribution of signal of $BODIPY_1$-Pep_4 and nuclear blue in cell lines	S4
Chemical experiments	S5–S12
Biological experiments	S13-S14
NMR spectra of products	S15–S31
HPLC chromatographs and MALID-TOF HRMS spectra of products	S32–S76

Emission spectra of $BODIPY_1$-Pep_4 in mixture solvent of buffer and DMSO

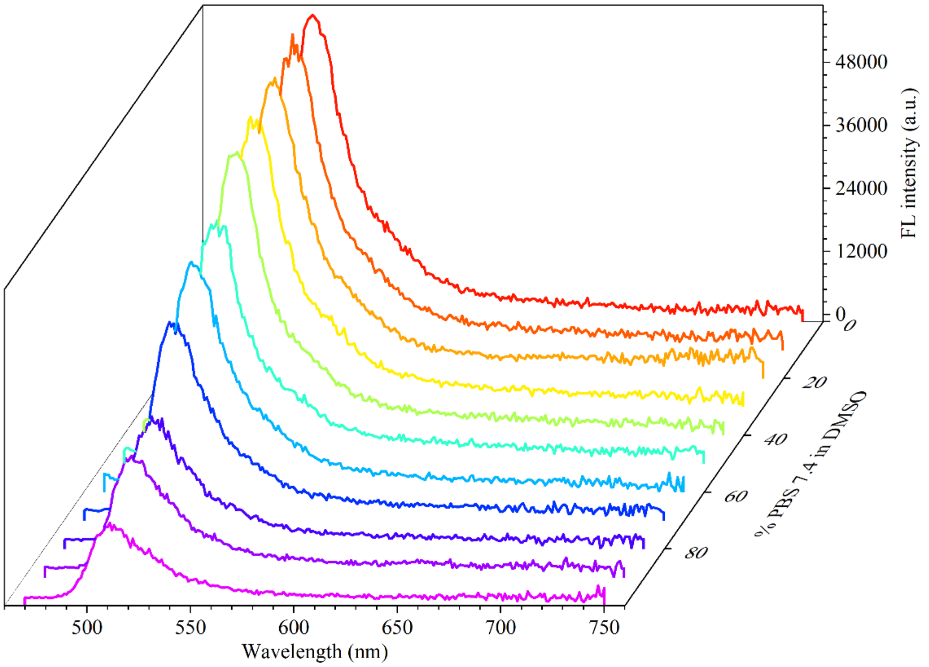

Fig. S1. Comparison of emission spectrum of **$BODIPY_1$-Pep_4** (1.2 uM) in solvents with different ratios of PBS7.4 and DMSO.

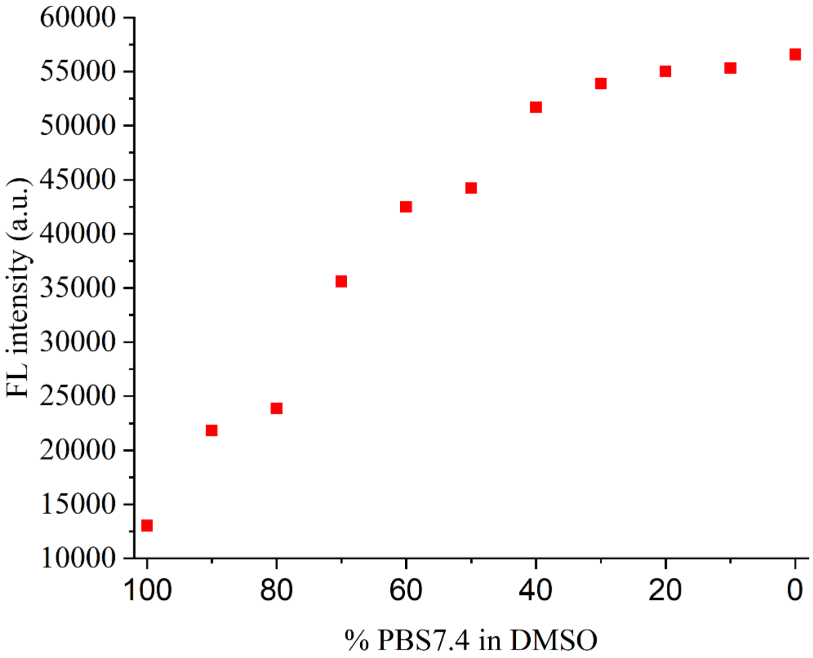

Fig. S2. Comparison of fluorescent intensity of **$BODIPY_1$-Pep_4** (1.2 uM) at 512 nm in solvents with different ratios of PBS7.4 and DMSO.

Photophysical properties of BODIPY-peptide conjugates

Table. S1. Photophysical property of BODIPY$_n$-Pep$_1$.[a]

	λ absorption (nm)	ε (× 10^4 M^{-1} cm^{-1})	λ excitation (nm)	λ emission (nm)	τ (ns)	Φ (%) [b]
**BODIPY$_1$-Pep$_1$**	503	5.81	501	515	3.18	50.1
**BODIPY$_3$-Pep$_1$**	526	5.10	526	542	5.23	34.0
**BODIPY$_9$-Pep$_1$**	560	4.07	557	599	0.991	5.58

[a.] all data are recorded in DMSO

[b.] the quantum yields are measured by comparative method with reference rhodamine 6G in water (Φ = 95%).

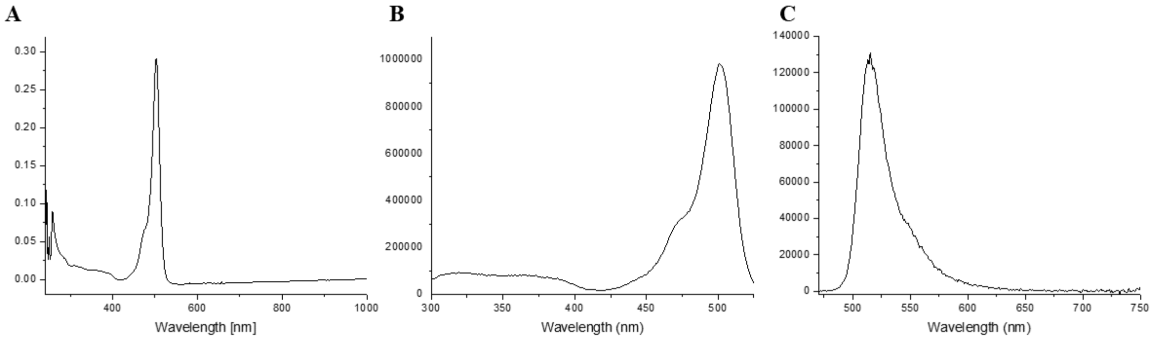

Fig. S3. Absorption (A), excitation (B) and emission (C) spectra of **BODIPY$_1$-Pep$_1$.**

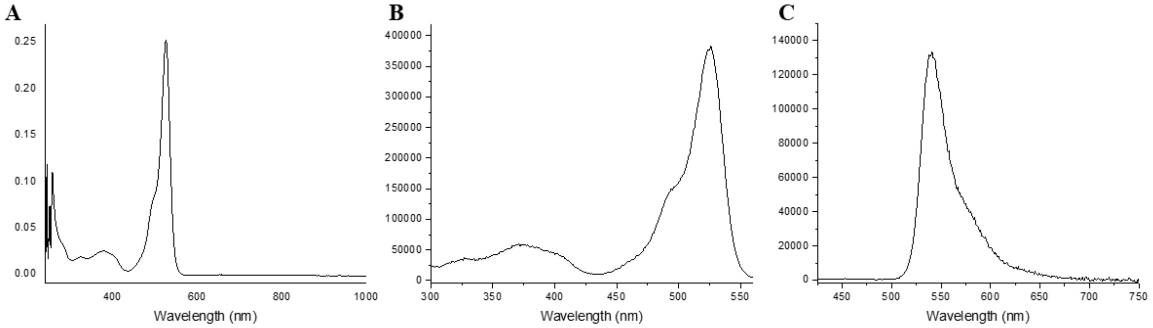

Fig. S4. Absorption (A), excitation (B) and emission (C) spectra of **BODIPY$_3$-Pep$_1$.**

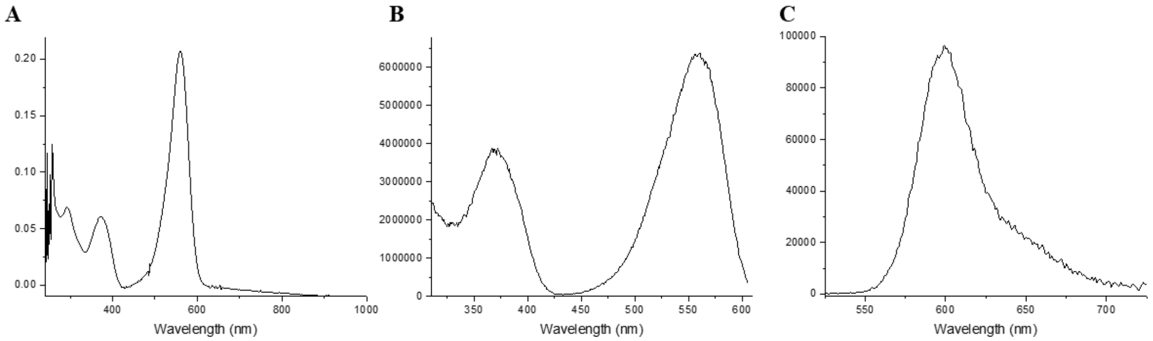

Fig. S5. Absorption (A), excitation (B) and emission (C) spectra of **BODIPY$_9$-Pep$_1$.**

Distribution of signal of BODIPY$_1$-Pep$_4$ and nuclear blue in the cell lines

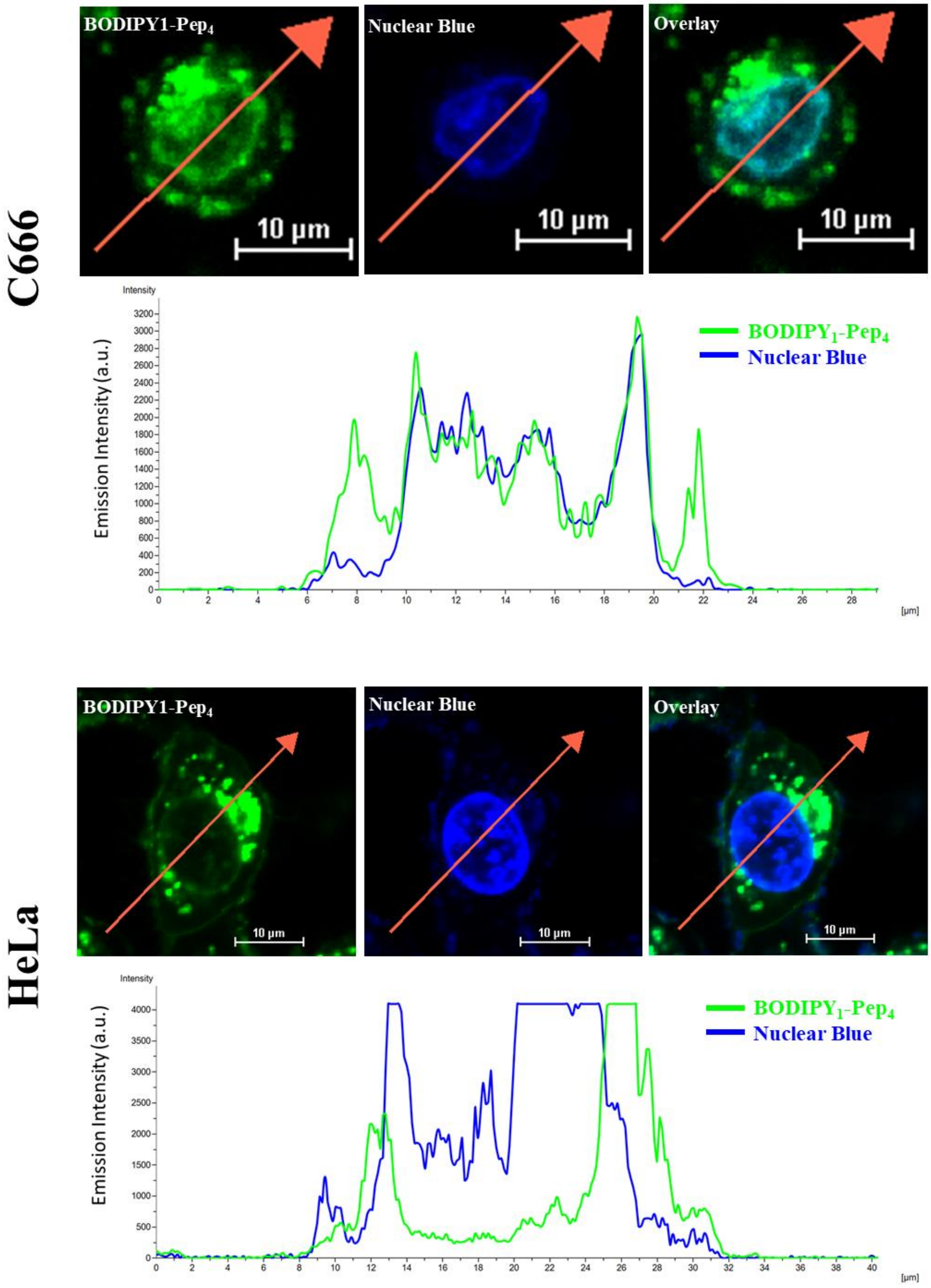

Fig. S6. Profiles of the emission intensity of the **BODIPY$_1$-Pep$_4$** and nuclear blue were plotted along the red arrow A. C666, B. HeLa. The signal of **BODIPY$_1$-Pep$_4$** and nuclear blue overlapped well in C666 cell line (EBNA+) but not in HeLa cell line (EBNA-).

Chemical Experiments

Reagents: All amino acid building blocks for Fmoc-strategy SPPS were purchased from Bidepharm. For synthesizing **Pep_1~Pep_{10}**, Fmoc-Ala-OH, Fmoc-Val-OH, Fmoc-Ile-OH, Fmoc-Leu-OH, Fmoc-Met-OH, Fmoc-Phe-OH, Fmoc-Tyr(tBu)-OH, Fmoc-Tyr(PO_3($MDPSE)_2$)-OH, Fmoc-Trp(Boc)-OH, Fmoc-Ser(tBu)-OH, Fmoc-Thr(tBu)-OH, Fmoc-Asn(Trt)-OH, Fmoc-Gln(Trt)-OH, Fmoc-Cys(Trt)-OH, Fmoc-Gly-OH, Fmoc-Pro-OH, Fmoc-Arg(Pdf)-OH, Fmoc-His(Boc)-OH, Fmoc-Lys(Boc)-OH, Fmoc-Asp(tBu)-OH, Fmoc-Glu(tBu)-OH, Fmoc-6-Ahx-OH, Fmoc-2-Nal-OH, Fmoc-4-Cpa-OH, Fmoc-3-Pal-OH, Fmoc-Cit-OH. For **Pep_{11}**, Fmoc-Lys(Alloc)-OH was used to take place Fmoc-Lys(Boc)-OH as building block of lysine. The Rink Amide resin (100-200 mesh) and Wang resin (100-200 mesh) were purchased from Sigma-Aldrich for synthesizing peptides with amide and acid C-terminal respectively. All aldehyde and pyrrole building blocks are commercially available except the pyrrole for synthesizing **DP_8** and **DP_{11}** are synthesized according literature.[1] Solvents and other reagent were purchased and used without further purification.

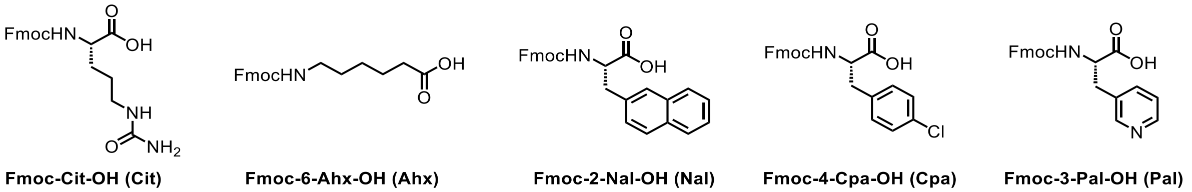

Fig. S7. Structure and abbreviate of unnatural amino acids.

Analytical HPLC: Analytical HPLC was performed on an Agilent 1100 series HPLC system (Agilent Technologies, Stockport, UK) equipped with a diode-array detection (DAD) detector and Agilent C18 column (250 mm x 4.6 mm) at the following gradients:

Gradient A:

**Time (min)**	**A % (H_2O + 0.1 % TFA)**	**B % (MeCN + 0.1 % TFA)**	**Flow (mL/min)**
0	80	20	0.5
60	0	100	0.5
70	0	100	0.5

Gradient B:

**Time (min)**	**A % (H_2O + 0.1 % TFA)**	**B % (MeCN + 0.1 % TFA)**	**Flow (mL/min)**
0	80	20	0.5
40	20	80	0.5
41	0	100	0.5
55	0	100	0.5

Preparative HPLC: The purifications of products were carried out on Waters semi-preparative system with Waters 2707 Autosampler, Water 1525 Binary HPLC Pump, Waters 2998 Photodiode Array Detector and Waters Fraction Collector III and Atlantis® T3 Prep OBD™ column (C18, 5 μm, 19×250 mm). The Gradient usually refer to the analytical HPLC but flow rate is 5 mL/min. The fractions were collected and verified by ESI-MS.

Mass spectrometry: High-resolution mass spectra, reported as m/z, were obtained from Bruker Autoflex MALDI-TOF mass spectrometer. Low-resolution mass spectra were conducted by SCIEX 3200Q ESI mass spectrometer was also used for monitoring reaction and determining correct fraction during purification of product.

Nuclear magnetic resonance: NMR spectra were recorded on a Bruker Ultrashield 400 Plus NMR spectrometer (^{1}H NMR on 400 MHz, ^{13}C NMR on 101 MHz and ^{19}F NMR on 376 mHz. The ^{1}H NMR chemical shifts were referenced to corresponding solvent peak (2.50 for DMSO-*d6* and 3.31 for methanol-*d4*). The following abbreviations were used to explain the multiplicities: s = singlet, d = doublet, t = triplet, q = quartet, dd = doublet of doublets, m = multiplet, br = broad.

General procedure of solid phase peptide synthesis: Standard Fmoc-based SPPS procedure was carried out manually. The 5 mL SPE filtration tube with Frits were used. The Rink Amide resin and Wang resin were used for synthesizing peptides with amide and acid C-terminal respectively. To load first amino acid onto Rink Amide resin, Fmoc-deprotected resin was shaken with amino acid building block (3 eq.), PyBOP (3 eq.) and DIPEA (6 eq.) in DMF (4 mL/ 0.1 mmol) overnight. To load first amino acid onto Wang resin, resin was shaken with amino acid building block (3 eq.), PyBOP (3 eq.) and DMAP (1 eq.) in DCM/DMF, v/v, 8/2 (4 mL/ 0.1 mmol) overnight. After loading first amino acid, the resin was washed with DMF (4 mL ×3) and DCM (4 mL ×3). Then, the shaken with Ac_2O/Pyridine, v/v, 3/2 (4 mL/ 0.1 mmol) for 30 min to cap all remining reacting site on resin. During peptide elongation, coupling was carried out with amino acid building block (3 eq.), PyBOP (3 eq.) and DIPEA (6 eq.) in DMF (4 mL/ 0.1 mmol) for 2~12 h, and Fmoc deprotection was carried out with 4 mL 20% piperidine in DMF (4 mL/ 0.1 mmol) for 25 min. After all amino acid building blocks were coupled, the substitute value of resin bound peptide was calculated by the method from literature[2]. Global cleavage was carried out with different cocktail (4 mL/ 0.1 mmol) and reaction time according different amino acid composition of each peptide. Cocktail A: TFA/TIPS/H_2O, v/v/v, 95/2.5/2.5; Cocktail B: TFA/EDT/p-Cresol/H_2O, v/v/v/v, 90/5/2.5/2.5.

H_2N-YFMVF-$CONH_2$ (Pep$_1$). Global cleavage condition: cocktail A, 2 h. Yield: 60 %. White powder. Analytic HPLC: Gradient A, retention time: 23.9 min, purity: 96.6 %. HRMS(MALDI-TOF): calc. for $C_{37}H_{49}N_6O_6S^+$ $[M+H]^+$ 705.3429, found 705.3489; calc. for $C_{37}H_{48}N_6NaO_6S^+$ $[M+Na]^+$ 727.3248, found 727.3251; calc. for $C_{37}H_{48}KN_6O_6S^+$ $[M+K]^+$ 743.2988, found 743.3027. ^{1}H NMR (400 MHz, Methanol-d_4) δ 7.26 – 7.14 (m, 10H), 7.11 – 7.04 (m, 2H), 6.81 – 6.72 (m, 2H), 4.89 (s, 1H), 4.70 (tt, *J* = 8.3, 5.9 Hz, 2H), 4.40 – 4.31 (m, 1H), 4.07 (dd, *J* = 8.6, 4.8 Hz, 1H), 3.20 – 3.05 (m, 3H), 2.98 – 2.79 (m, 3H), 2.56 – 2.38 (m, 2H), 2.01 (s, 6H), 0.90 (dd, *J* = 6.8, 5.1 Hz, 6H); ^{13}C NMR (101 MHz, Methanol-d_4) δ 129.58, 128.28, 128.22, 127.43, 127.34, 125.74, 114.80, 53.39, 47.52, 47.31, 47.10, 46.89, 46.67, 46.46, 46.25, 37.23, 37.05, 35.71, 31.38, 30.27, 28.97, 17.65, 16.99, 13.27; ^{1}H NMR (400 MHz, DMSO-d_6) δ 9.38 (s, 1H), 8.77 (d, *J* = 8.1 Hz, 1H), 8.44 (d, *J* = 7.9 Hz, 1H), 8.02 (d, *J* = 8.2 Hz, 1H), 7.99 – 7.92 (m, 2H), 7.83 (d, *J* = 8.8 Hz, 1H), 7.38 (d, *J* = 2.2 Hz, 1H), 7.30 – 7.15 (m, 10H), 7.06 (s, 1H), 7.04 (d, *J* = 8.5 Hz, 2H), 6.72 – 6.67 (m, 2H), 4.65 (td, *J* = 8.7, 4.2 Hz, 1H), 4.50 – 4.42 (m, 2H), 4.18 – 4.13 (m, 1H), 3.89 (s, 1H), 3.08 – 2.93 (m, 3H), 2.82 (ddd, *J* = 14.0, 9.3, 6.6 Hz, 3H), 2.42 (ddp, *J* = 13.1, 9.3, 6.9, 6.2 Hz, 2H), 2.03 (s, 3H), 1.97 – 1.87 (m, 2H), 1.79 (dtd, *J* = 14.2, 9.4, 6.1 Hz, 1H), 0.79 (dd, *J* = 6.8, 2.2 Hz, 6H); ^{13}C NMR (101 MHz, Methanol-d_4) δ 129.58, 128.28, 128.22, 127.43, 127.34, 125.74, 114.80, 53.39, 47.52, 47.31, 47.10, 46.89, 46.67, 46.46, 46.25, 37.23, 37.05, 35.71, 31.38, 30.27, 28.97, 17.65, 16.99, 13.27; ^{13}C NMR (101 MHz, DMSO-d_6) δ 172.61, 170.66, 170.58, 170.40, 156.52, 137.70, 137.37, 130.53, 129.19, 129.00,

128.06, 127.91, 126.32, 126.14, 124.42, 115.25, 57.57, 53.96, 53.45, 53.23, 51.93, 37.57, 37.39, 36.08, 31.92, 30.59, 29.47, 19.08, 17.90, 14.58.

H_2N-C-Ahx-RrRKGGYFMVF-COOH (Pep$_2$). Global cleavage condition: cocktail B, 4 h. Yield: 54 %. White powder. Analytic HPLC: Gradient B, retention time: 16.8 min, purity: 98.4 %. HRMS(MALDI-TOF): calc. for $C_{71}H_{110}N_{19}O_{14}^+$ $[M+H]^+$ 1452.8474, found 1452.8474; calc. for $C_{71}H_{109}N_{19}NaO_{14}^+$ $[M+Na]^+$ 1474.8294, found 1474.8294.

H_2N-C-Ahx-YFMVFGGRrRK-COOH (Pep$_3$). Global cleavage condition: cocktail B, 4 h. Yield: 60 %. White powder. Analytic HPLC: Gradient B, retention time: 18.9 min, purity: 97.1 %. HRMS(MALDI-TOF): calc. for $C_{74}H_{118}N_{23}O_{15}S_2^+$ $[M+H]^+$ 1632.8614, found 1632.8623; calc. for $C_{74}H_{117}N_{23}NaO_{15}S_2^+$ $[M+Na]^+$ 1654.8433, found 1654.8431.

H_2N-C-Ahx-YFMVFGGRrRK-COOH (Pep$_4$). Global cleavage condition: cocktail B, 4 h. Yield: 53 %. White powder. Analytic HPLC: Gradient B, retention time: 17.6 min, purity: 95.9 %. HRMS(MALDI-TOF): calc. for $C_{74}H_{118}N_{23}O_{15}S_2^+$ $[M+H]^+$ 1632.8614, found 1632.8604; calc. for $C_{74}H_{117}N_{23}NaO_{15}S_2^+$ $[M+Na]^+$ 1654.8433, found 1654.8440.

H_2N-NWYFIVF-COOH (Pep$_5$). Global cleavage condition: cocktail A, 2 h. Yield: 57 %. White powder. Analytic HPLC: Gradient A, retention time: 28.7 min, purity: 94.9 %. HRMS(MALDI-TOF): calc. for $C_{58}H_{73}N_{11}NaO_{12}^+$ $[M+Na]^+$ 1138.5332, found 1138.5323.

H_2N-DEHYFIVF-COOH (Pep$_6$). Global cleavage condition: cocktail A, 2 h. Yield: 60 %. White powder. Analytic HPLC: Gradient A, retention time: 22.8 min, purity: 96.8 %. HRMS(MALDI-TOF): calc. for $C_{53}H_{69}N_{10}O_{14}^+$ $[M+H]^+$ 1069.4989, found 1069.4988; calc. for $C_{53}H_{68}KN_{10}O_{14}^+$ $[M+K]^+$ 1107.4548, found 1107.4424.

H_2N-Ahx-P-(pTyr)-LKTK-COOH (Pep$_7$). Global cleavage condition: cocktail B, 3 h. Yield: 59 %. White powder. Analytic HPLC: Gradient B, retention time: 8.4 min, purity: 93.7 %. HRMS(MALDI-TOF): calc. for $C_{42}H_{73}N_9O_{13}P^+$ $[M+H]^+$ 942.5060, found 942.5063; calc. for $C_{42}H_{72}N_9NaO_{13}P^+$ $[M+Na]^+$ 964.4879, found 964.4882; calc. for $C_{42}H_{72}KN_9O_{13}P^+$ $[M+K]^+$ 980.4619, found 980.4627.

H_2N-Ahx-P-(pTyr)-LKTKRrRK-COOH (Pep$_8$). Global cleavage condition: cocktail B, 3 h. Yield: 62 %. White powder. Analytic HPLC: Gradient B, retention time: 33.5 min, purity: 98.4 %. HRMS(MALDI-TOF): calc. for $C_{66}H_{121}N_{23}O_{17}P^+$ $[M+H]^+$ 1538.9043, found 1538.9043; calc. for $C_{66}H_{120}N_{23}NaO_{17}P^+$ $[M+Na]^+$ 1560.8862, found 1560.8860.

H_2N-Nal-Cpa-Pal-SY-Cit-LRPA-CONH$_2$ (Pep$_9$). Global cleavage condition: cocktail A, 3 h. Yield: 64 %. White powder. Analytic HPLC: Gradient B, retention time: 16.3 min, purity: 99.0 %. HRMS(MALDI-TOF): calc. for $C_{68}ClH_{91}N_{17}O_{13}^+$ $[M+H]^+$ 1388.6665, found 1388.6555; calc. for $C_{68}ClH_{90}N_{17}NaO_{13}^+$ $[M+Na]^+$ 1410.6485, found 1410.6362.

H_2N-DRVYIHPF-CONH$_2$ (Pep$_{10}$). Global cleavage condition: cocktail A, 2 h. Yield: 58 %. White powder. Analytic HPLC: Gradient B, retention time: 13.3 min, purity: 96.2 %. HRMS(MALDI-TOF): calc. for $C_{50}H_{73}N_{14}O_{11}^+$ $[M+H]^+$ 1045.5578, found 1045.5859; calc. for $C_{50}H_{72}N_{14}NaO_{11}^+$ $[M+Na]^+$ 1067.5397, found 1067.5725.

H_2N- GHK-CONH$_2$ (Pep$_{11}$). Before global cleavage, Alloc deprotection was required. Soak resin in 4 mL DCM, the $Pd(PPh_3)_4$ (0.5 eq) and $PhSiH_3$ (10 eq.) was added. The SPE tube was sealed tightly, and shaken overnight. After reaction, little resin was taken for cleaving, and resulting solution was verified by ESI-MS to check if Alloc was deprotected completely. Global cleavage condition: cocktail A, 2 h. Yield: 70 %. White powder. Analytic HPLC: Gradient B, retention time: 6.0 min, purity: 92.8 %. HRMS(MALDI-TOF): calc. for $C_{14}H_{26}N_7O_3^+$ $[M+H]^+$ 340.2092, found 340.2039; calc. for $C_{14}H_{25}N_7NaO_3^+$ $[M+Na]^+$ 362.1911, found 362.1856. ^{1}H NMR (400 MHz, DMSO-d_6) δ 14.51 (s, 2H), 8.97

(t, J = 1.1 Hz, 1H), 8.68 (d, J = 8.0 Hz, 1H), 8.27 (d, J = 7.5 Hz, 1H), 8.07 (t, J = 6.0 Hz, 3H), 7.82 (s, 3H), 7.55 (s, 1H), 7.37 (s, 1H), 7.19 (s, 1H), 4.71 (q, J = 6.8 Hz, 1H), 4.14 (td, J = 8.3, 5.0 Hz, 1H), 3.59 (d, J = 5.6 Hz, 2H), 3.04 (qd, J = 15.3, 6.2 Hz, 2H), 2.75 (q, J = 6.5 Hz, 2H), 1.74 – 1.41 (m, 4H), 1.30 (q, J = 9.5, 8.1 Hz, 2H); ^{13}C NMR (101 MHz, DMSO-d_6) δ 173.65, 169.17, 165.91, 133.76, 128.64, 117.10, 52.47, 51.48, 39.97 (read from HSQC), 38.53, 31.09, 27.44, 26.57, 22.30.

Synthetic procedure of symmetric dipyrrin peptide conjugates: Resin-bound peptide (0.1 mmol) from SPPS was placed in SPE tube, then the aldehyde building block was coupled as routine coupling procedure of SPPS. Upon completion, the liquid phase was removed, and the resin was washed with DMF (4 mL ×3) and DCM (4 mL ×3), little part of resin could be taken and be cleaved as a reference. The resin was soared in 4 mL DMF, then pyrrole building block (1.0 mmol) and BF_3.OEt_2 (0.1 mmol) was added into SPE tube directly by pipette. The SPE tube was shaken for 3-12 h (usually overnight) in dark. After that, the liquid phase was removed by filtration and resin was washed with DMF (4 mL ×3) and DCM (4 mL ×3). Resin was soared in DCM, and DDQ (0.5 mmol) solid was added directly and shaking for 1 h. The resin was washed thoroughly with DMF and DCM until liquid phase was colorless. Finally, 4 mL cleaving cocktail was use for global cleavage. The crude product was purified by preparative HPLC, dry crude product may used for boron complexation directly.

DP_1-HN-YFMVF-$CONH_2$ (DP_1-Pep_1). Global cleavage condition: cocktail A, 2 h. Yield: 51 %. Red powder. Analytic HPLC: Gradient A, retention time: 33.3 min, purity: 96.3 %. HRMS(MALDI-TOF): calc. for $C_{57}H_{67}N_8O_7S^+$ $[M+H]^+$ 1007.4848, found 1007.4843.

DP_2-HN-YFMVF-$CONH_2$ (DP_2-Pep_1). Global cleavage condition: cocktail A, 2 h. Yield: 41 %. Orange powder. Analytic HPLC: Gradient A, retention time: 31.3 min, purity: 94.0 %. HRMS(MALDI-TOF): calc. for $C_{55}H_{63}N_8O_7S^+$ $[M+H]^+$ 979.4535, found 979.4522; calc. for $C_{55}H_{62}N_8NaO_7S^+$ $[M+Na]^+$ 1001.4354, found 1001.4316.

DP_3-HN-YFMVF-$CONH_2$ (DP_3-Pep_1). Global cleavage condition: cocktail A, 2 h. Yield: 53 %. Red powder. Analytic HPLC: Gradient A, retention time: 39.3 min, purity: 96.6 %. HRMS(MALDI-TOF): calc. for $C_{61}H_{75}N_8O_7S^+$ $[M+H]^+$ 1063.5474, found 1063.5477; calc. for $C_{61}H_{74}N_8NaO_7S^+$ $[M+Na]^+$ 1085.5293, found 1085.5297.

DP_4-HN-YFMVF-$CONH_2$ (DP_4-Pep_1). Global cleavage condition: cocktail A, 2 h. Yield: 10 %. Yellow powder. Analytic HPLC: Gradient A, retention time: 28.9 min, purity: 96.0 %. HRMS(MALDI-TOF): calc. for $C_{53}H_{59}N_8O_7S^+$ $[M+H]^+$ 951.4222, found 951.4234; calc. for $C_{53}H_{58}N_8NaO_7S^+$ $[M+Na]^+$ 973.4041, found 973.4025.

DP_5-HN-YFMVF-$CONH_2$ (DP_5-Pep_1). Global cleavage condition: cocktail A, 2 h. Yield: 51 %. Red powder. Analytic HPLC: Gradient A, retention time: 32.6 min, purity: 97.6 %. HRMS(MALDI-TOF): calc. for $C_{61}H_{63}N_8O_7S^+$ $[M+H]^+$ 1051.4535, found 1051.4539; calc. for $C_{61}H_{62}N_8NaO_7S^+$ $[M+Na]^+$ 1073.4354, found 1073.4337.

DP_6-HN-YFMVF-$CONH_2$ (DP_6-Pep_1). Global cleavage condition: cocktail A, 2 h. Yield: 50 %. Red powder. Analytic HPLC: Gradient A, retention time: 33.2 min, purity: 94.8 %. HRMS(MALDI-TOF): calc. for $C_{63}H_{67}N_8O_9S^+$ $[M+H]^+$ 1111.4746, found 1111.4750.

DP_9-HN-YFMVF-$CONH_2$ (DP_9-Pep_1). Global cleavage condition: cocktail A, 2 h. Yield: 35 %. Purple powder. Analytic HPLC: Gradient A, retention time: 40.2 min, purity: 95.2 %. HRMS(MALDI-TOF): calc. for $C_{65}H_{67}N_8O_7S^+$ $[M+H]^+$ 1103.4848, found 1103.5282.

DP_{10}-HN-YFMVF-$CONH_2$ (DP_{10}-Pep_1). Global cleavage condition: cocktail A, 2 h. Yield: 47 %. Red powder. Analytic HPLC: Gradient A, retention time: 32.4 min, purity: 95.2 %. HRMS(MALDI-TOF): calc. for $C_{57}H_{67}N_8O_7S^+$ $[M+H]^+$ 1007.4848, found 1007.4864; calc. for $C_{57}H_{66}N_8NaO_7S^+$ $[M+Na]^+$ 1029.4667, found 1029.4450.

DP_{12}-HN-YFMVF-$CONH_2$ (DP_{12}-Pep_1). Global cleavage condition: cocktail A, 2 h. Yield: 40 %. Red powder. Analytic HPLC: Gradient A, retention time: 33.5 min, purity: 96.0 %. HRMS(MALDI-TOF): calc. for $C_{58}H_{69}N_8O_8S^+$ $[M+H]^+$ 1037.4954, found 1037.4929.

DP_{13}-HN-YFMVF-$CONH_2$ (DP_{13}-Pep_1). Global cleavage condition: cocktail A, 2 h. Yield: 40 %. Red powder. Analytic HPLC: Gradient A, retention time: 32.8 min, purity: 97.6 %. HRMS(MALDI-TOF): calc. for $C_{58}H_{69}N_8O_8S^+$ $[M+H]^+$ 1037.4954, found 1037.4922; calc. for $C_{58}H_{68}N_8NaO_8S^+$ $[M+Na]^+$ 1059.4773, found 1059.4777.

DP_{14}-HN-YFMVF-$CONH_2$ (DP_{14}-Pep_1). Global cleavage condition: cocktail A, 2 h. Yield: 34 %. Orange powder. Analytic HPLC: Gradient A, retention time: 33.1 min, purity: 96.4 %. HRMS(MALDI-TOF): calc. for $C_{57}H_{70}N_9O_7S^+$ $[M+H]^+$ 1024.5113, found 1024.5127.

DP_1-HN-C-Ahx-RrRKGGYFMVF-COOH (DP_1-Pep_2). Global cleavage condition: cocktail B, 4 h. Yield: 44 %. Red powder. Analytic HPLC: Gradient B, retention time: 23.1 min, purity: 95.8 %. HRMS(MALDI-TOF): calc. for $C_{91}H_{128}N_{21}O_{15}^+$ $[M+H]^+$ 1755.9927, found 1755.9933.

DP_1-HN-C-Ahx-YFMVFGGRrRK-COOH (DP_1-Pep_3). Global cleavage condition: cocktail B, 4 h. Yield: 49 %. Red powder. Analytic HPLC: Gradient B, retention time: 21.8 min, purity: 97.0 %. HRMS(MALDI-TOF): calc. for $C_{94}H_{136}N_{25}O_{16}S_2^+$ $[M+H]^+$ 1936.0066, found 1936.0118.

DP_1-HN-C-Ahx-YFMVFGGRrRK-COOH (DP_1-Pep_4). Global cleavage condition: cocktail B, 4 h. Yield: 42 %. Red powder. Analytic HPLC: Gradient B, retention time: 22.2 min, purity: 96.6 %. HRMS(MALDI-TOF): calc. for $C_{94}H_{136}N_{25}O_{16}S_2^+$ $[M+H]^+$ 1936.0066, found 1936.0017.

DP_1-HN-NWYFIVF-COOH (DP_1-Pep_5). Global cleavage condition: cocktail A, 2 h. Yield: 47 %. Red powder. Analytic HPLC: Gradient A, retention time: 34.2 min, purity: 99.0 %. HRMS(MALDI-TOF): calc. for $C_{78}H_{92}N_{13}O_{13}^+$ $[M+H]^+$ 1418.6932, found 1418.6955.

DP_1-HN-DEHYFIVF-COOH (DP_1-Pep_6). Global cleavage condition: cocktail A, 2 h. Yield: 50 %. Red powder. Analytic HPLC: Gradient A, retention time: 27.3 min, purity: 96.7 %. HRMS(MALDI-TOF): calc. for $C_{73}H_{87}N_{12}O_{15}^+$ $[M+H]^+$ 1371.6408, found 1371.6353; calc. for $C_{73}H_{86}N_{12}NaO_{15}^+$ $[M+Na]^+$ 1393.6228, found 1393.6185.

DP_1-HN-Ahx-P-(pTyr)-LKTK-COOH (DP_1-Pep_7). Global cleavage condition: cocktail B, 3 h. Yield: 47 %. Red powder. Analytic HPLC: Gradient B, retention time: 17.6 min, purity: 98.2 %. HRMS(MALDI-TOF): calc. for $C_{62}H_{91}N_{11}O_{14}P^+$ $[M+H]^+$ 1244.6479, found 1244.6422.

DP_1-HN-Ahx-P-(pTyr)-LKTKRrRK-COOH (DP_1-Pep_8). Global cleavage condition: cocktail B, 3 h. Yield: 48 %. Red powder. Analytic HPLC: Gradient B, retention time: 25.0 min, purity: 96.8 %. HRMS(MALDI-TOF): calc. for $C_{86}H_{139}N_{25}O_{18}P^+$ $[M+H]^+$ 1842.0496, found 1842.0535.

DP_1-HN-DRVYIHPF-$CONH_2$ (DP_1-Pep_{10}). Global cleavage condition: cocktail A, 2 h. Yield: 53 %. Red powder. Analytic HPLC: Gradient B, retention time: 15.8 min, purity: 98.9 %. HRMS(MALDI-TOF): calc. for $C_{70}H_{91}N_{16}O_{12}^+$ $[M+H]^+$ 1347.6997, found 1347.7072.

DP_1-HN-GHK-$CONH_2$ (DP_1-Pep_{11}). Before global cleavage, Alloc deprotection was required (the procedure described on **Pep_{11}**). Global cleavage condition: cocktail A, 2 h. Yield: 59 %. Red powder. Analytic HPLC: Gradient B, retention time: 12.1 min, purity: 95.9 %. HRMS(MALDI-TOF): calc. for $C_{34}H_{44}N_9O_4^+$ $[M+H]^+$ 642.3511, found 642.3905; calc. for $C_{34}H_{43}N_9NaO_4^+$ $[M+Na]^+$ 664.3330, found

664.3734. ^{1}H NMR (400 MHz, DMSO-d_6) δ 14.44 (s, 2H), 12.20 (s, 2H), 9.10 (t, *J* = 5.8 Hz, 1H), 9.00 (d, *J* = 1.5 Hz, 1H), 8.38 (d, *J* = 8.0 Hz, 1H), 8.13 (d, *J* = 7.5 Hz, 1H), 8.11 – 8.02 (m, 2H), 7.81 (s, 3H), 7.53 (d, *J* = 8.1 Hz, 2H), 7.50 (d, *J* = 2.0 Hz, 1H), 7.41 (d, *J* = 1.3 Hz, 1H), 7.22 – 7.15 (m, 1H), 6.53 (s, 2H), 4.65 (td, *J* = 7.8, 5.4 Hz, 1H), 4.16 (td, *J* = 8.5, 4.9 Hz, 1H), 3.94 (d, *J* = 5.7 Hz, 2H), 3.20 – 2.98 (m, 2H), 2.78 (h, *J* = 6.0 Hz, 2H), 2.43 (s, 6H), 1.83 – 1.38 (m, 10H), 1.33 (q, *J* = 9.2, 8.3 Hz, 2H); ^{13}C NMR (101 MHz, DMSO-d_6) δ 173.67, 169.67, 168.94, 165.75, 152.41, 145.34, 142.32, 138.40, 136.91, 133.69, 129.09, 127.93, 120.48, 116.98, 52.48, 51.49, 42.70, 38.59, 31.07, 27.07, 26.60, 22.31, 13.79, 13.73.

DP_1-HN-GHK-$CONH_2$ (DP_1-Pep_{11})*. Fmoc and Alloc dual-protected peptide on resin was used. Alloc deprotection was conducted as procedure described on **Pep_{11}**. Then, the dipyrrin was constructed as general procedure above. Fmoc was deprotected before global cleavage. Global cleavage condition: cocktail A, 2 h. Yield: 51 %. Red powder. Analytic HPLC: Gradient B, retention time: 13.1 min, purity: 98.9 %. HRMS(MALDI-TOF): calc. for $C_{34}H_{44}N_9O_4^+$ [M+H]$^+$ 642.3511, found 642.3593; calc. for $C_{34}H_{43}N_9NaO_4^+$ [M+Na]$^+$ 664.3330, found 664.3391. ^{1}H NMR (400 MHz, DMSO-d_6) δ 14.38 (s, 2H), 12.16 (s, 2H), 8.99 (s, 1H), 8.78 (t, *J* = 5.7 Hz, 1H), 8.68 (d, *J* = 8.0 Hz, 1H), 8.29 (d, *J* = 7.2 Hz, 1H), 8.04 (s, 3H), 8.02 (s, 2H), 7.58 (s, 1H), 7.50 (d, *J* = 8.0 Hz, 2H), 7.40 (s, 1H), 7.21 (s, 1H), 6.53 (s, 2H), 4.74 (q, *J* = 6.7 Hz, 1H), 4.19 – 4.15 (m, 1H), 3.63 (s, 2H), 3.27 (t, *J* = 6.8 Hz, 2H), 3.09 – 3.01 (m, 2H), 2.43 (s, 6H), 1.60 (ddp, *J* = 37.7, 15.0, 7.7, 6.9 Hz, 10H), 1.39 – 1.30 (m, 2H).

Synthetic procedure of asymmetric dipyrrin peptide conjugates: Resin-bound peptide was loaded in SPE tube, then the 5-formyl-2,4-dimethyl-3-pyrrolecarboxylic acid was coupled as general coupling procedure of SPPS. Upon completion, the liquid phase was removed by filtration, and the resin was washed with DMF (4 mL ×3) and DCM (4 mL ×3). The resin was soaked in DCM, and the pyrrole building block (0.5 mmol) and $POCl_3$ (0.5 mmol) was added by pipette into SPE tube directly. The mixture was shaken overnight in dark. Then, the resin was washed thoroughly with DMF and DCM until liquid phase was colorless, suitable cleavage cocktail was use for global cleavage. The crude product was purified by preparative HPLC.

DP_{15}-HN-YFMVF-$CONH_2$ (DP_{15}-Pep_1). Global cleavage condition: cocktail A, 2 h. Yield: 45 %. Yellow powder. Analytic HPLC: Gradient A, retention time: 30.3 min, purity: 95.2 %. HRMS(MALDI-TOF): calc. for $C_{51}H_{62}N_8NaO_7S^+$ [M+Na]$^+$ 953.4354, found 953.4309; calc. for $C_{51}H_{62}KN_8O_7S^+$ [M+K]$^+$ 969.4094, found 969.4078.

DP_{16}-HN-YFMVF-$CONH_2$ (DP_{16}-Pep_1). Global cleavage condition: cocktail A, 2 h. Yield: 42 %. Red powder. Analytic HPLC: Gradient A, retention time: 31.4 min, purity: 95.1 %. HRMS(MALDI-TOF): calc. for $C_{54}H_{63}N_8O_8S^+$ [M+H]$^+$ 983.4484, found 983.5748; calc. for $C_{54}H_{62}N_8NaO_8S^+$ [M+Na]$^+$ 1005.4304, found 1005.5553. ^{1}H NMR (400 MHz, DMSO-d_6) δ 13.78 (s, 1H), 12.55 (s, 1H), 9.03 (s, 1H), 8.48 (s, 1H), 8.28 (d, *J* = 7.8 Hz, 2H), 8.21 (dd, *J* = 8.3, 3.3 Hz, 1H), 8.00 (d, *J* = 8.3 Hz, 1H), 7.87 (d, *J* = 2.4 Hz, 1H), 7.82 (d, *J* = 8.7 Hz, 1H), 7.54 (d, *J* = 8.8 Hz, 1H), 7.36 (d, *J* = 2.3 Hz, 1H), 7.25 – 7.18 (m, 10H), 7.08 (d, *J* = 8.4 Hz, 2H), 7.05 (s, 1H), 7.01 (dd, *J* = 8.8, 2.4 Hz, 1H), 6.66 (d, *J* = 8.4 Hz, 2H), 4.67 – 4.61 (m, 2H), 4.48 (m, 1H), 4.41 (m, 1H), 4.17 – 4.14 (m, 1H), 3.88 (s, 3H), 3.00 (td, *J* = 8.5, 7.9, 3.7 Hz, 3H), 2.81 (d, *J* = 9.1 Hz, 3H), 2.41 (td, *J* = 7.3, 6.7, 3.1 Hz, 2H), 2.35 (s, 3H), 2.30 (s, 3H), 2.02 (s, 3H), 1.94 – 1.88 (m, 2H), 1.80 – 1.76 (m, 1H), 0.79 (dd, *J* = 6.8, 4.0 Hz, 6H).

General procedure of boron complexation of dipyrrin conjugates: To a suspension of dipyrrin peptide conjugate (0.02 mmol) in acetonitrile (1.0 mL), DIPEA (170 uL) was added by pipette. The mixture was shaken or sonicated for 5 min. $BF_3.OEt_2$ (280 uL) was added by pipette in one portion. The mixture was

further shaken for 10 min. The crude product was concentrated under vacuum and was purified by preparative HPLC.

BODIPY$_1$-HN-YFMVF-CONH$_2$ (BODIPY$_1$-Pep$_1$). Yield: 73 %. Red powder. Analytic HPLC: Gradient A, retention time: 47.5 min, purity: 97.0 %. HRMS(MALDI-TOF): calc. for $BC_{57}H_{68}N_8O_9S^+$ [M-2F+2OH+H]$^+$ 1051.4987, found 1051.4934; calc. for $BC_{57}F_2H_{65}KN_8O_7S^+$ [M+K]$^+$ 1093.4460, found 1093.4703. ^{1}H NMR (400 MHz, DMSO-d_6) δ 9.20 (s, 1H), 8.74 (d, J = 8.2 Hz, 1H), 8.27 (dd, J = 28.2, 7.9 Hz, 2H), 8.05 (s, 1H), 8.02 (d, J = 8.4 Hz, 2H), 7.85 (d, J = 8.7 Hz, 1H), 7.55 (d, J = 8.1 Hz, 2H), 7.47 – 7.37 (m, 1H), 7.35 – 7.19 (m, 10H), 7.18 – 7.15 (m, 2H), 7.14 – 7.05 (m, 1H), 6.71 – 6.64 (m, 2H), 6.25 (s, 2H), 4.64 (dtd, J = 24.6, 8.6, 4.1 Hz, 2H), 4.54 (td, J = 8.6, 5.2 Hz, 1H), 4.47 (td, J = 8.3, 4.9 Hz, 1H), 4.20 (dd, J = 8.7, 6.7 Hz, 1H), 3.18 – 2.97 (m, 3H), 2.95 – 2.85 (m, 3H), 2.52 (s, 6H), 2.46 (tt, J = 6.6, 2.9 Hz, 2H), 2.07 (s, 3H), 2.01 – 1.92 (m, 2H), 1.84 (dt, J = 13.3, 4.7 Hz, 1H), 1.39 (s, 6H), 0.84 (dd, J = 6.8, 2.6 Hz, 6H); ^{13}C NMR (101 MHz, DMSO-d_6) δ 172.65, 171.48, 170.88, 170.75, 170.45, 165.61, 155.68, 155.13, 142.63, 141.07, 137.76, 136.91, 134.52, 130.39, 130.06, 129.26, 129.06, 128.37, 127.98, 127.91, 126.20, 121.51, 114.83, 57.66, 55.29, 53.90, 53.49, 51.91, 37.61, 37.23, 35.98, 32.01, 30.60, 29.49, 29.01, 19.13, 17.99, 14.65, 14.22, 14.15; ^{19}F NMR (376 MHz, DMSO-d_6) δ -145.48 (dd, J = 65.6, 30.6 Hz).

BODIPY$_1$-HN-YFMVF-CONH$_2$ (BODIPY$_1$-Pep$_1$). Yield: 73 % (from purified **DP$_1$-Pep$_1$**) or 45 % (from dry crude product of **DP$_1$-Pep$_1$** from SPPS) Red powder. Analytic HPLC: Gradient A, retention time: 47.5 min, purity: 97.0 %. HRMS(MALDI-TOF): calc. for $C_{57}H_{68}BN_8O_9S^+$ [M-2F+2OH+H]$^+$ 1051.4987, found 1051.4934; calc. for $C_{57}H_{65}BF_2KN_8O_7S^+$ [M+K]$^+$ 1093.4460, found 1093.4703.

BODIPY$_2$-HN-YFMVF-CONH$_2$ (BODIPY$_2$-Pep$_1$). Yield: 76 %. Orange powder. Analytic HPLC: Gradient A, retention time: 46.2 min, purity: 95.0 %. HRMS(MALDI-TOF): calc. for $BC_{55}FH_{61}N_8O_7S^+$ [M-F]$^+$ 1007.4525, found 1007.4451; calc. for $C_{55}H_{61}BF_2N_8NaO_7S^+$ [M+Na]$^+$ 1049.4407, found 1049.3821; calc. for $C_{55}H_{61}BF_2KN_8O_7S^+$ [M+K]$^+$ 1065.4147, found 1065.4182.

BODIPY$_3$-HN-YFMVF-CONH$_2$ (BODIPY$_3$-Pep$_1$). Yield: 70 %. Red powder. Analytic HPLC: Gradient A, retention time: 54.7 min, purity: 96.0 %. HRMS(MALDI-TOF): calc. for $C_{61}H_{75}N_8O_7S^+$ [M-BF2+2H]$^+$ 1063.5474, found 1063.5389; calc. for $C_{61}H_{73}BF_2N_8NaO_7S^+$ [M+Na]$^+$ 1133.5346, found 1133.5359; calc. for $C_{61}H_{73}BF_2KN_8O_7S^+$ [M+K]$^+$ 1149.5086, found 1149.5101.

BODIPY$_9$-HN-YFMVF-CONH$_2$ (BODIPY$_9$-Pep$_1$). Yield: 55 %. Purple powder. Analytic HPLC: Gradient A, retention time: 53.0 min, purity: 94.6 %. HRMS(MALDI-TOF): calc. for $BC_{65}H_{68}N_8O_9S^+$ [M-2F+2OH+H]$^+$ 1147.4987, found 1147.5021; calc. for $C_{65}H_{65}BF_2N_8NaO_7S^+$ [M+Na]$^+$ 1173.4720, found 1173.4887; calc. for $C_{65}H_{65}BF_2KN_8O_7S^+$ [M+K]$^+$ 1189.4460, found 1189.4789.

BODIPY$_{15}$-HN-YFMVF-CONH$_2$ (BODIPY$_{15}$-Pep$_1$). Yield: 71 %. Orange powder. Analytic HPLC: Gradient A, retention time: 42.8 min, purity: 94.1 %. HRMS(MALDI-TOF): calc. for $BC_{51}FH_{61}N_8O_7S^+$ [M-F]$^+$ 959.4525, found 959.5180; calc. for $C_{51}H_{61}BF_2N_8NaO_7S^+$ [M+Na]$^+$ 1001.4407, found 1001.5133; calc. for $BC_{51}F_2H_{61}KN_8O_7S^+$ [M+K]$^+$ 1017.4147, found 1017.5093.

BODIPY$_1$-HN-C-Ahx-YFMVFGGRrRK-COOH (BODIPY$_1$-Pep$_4$). Yield: 49 %. EDT was used as reductant during reaction to prevent the oxidation of Cys. Red powder. Analytic HPLC: Gradient B, retention time: 29.5 min, purity: 98.7 %. HRMS(MALDI-TOF): calc. for $C_{94}H_{135}BF_2N_{25}O_{16}S_2^+$ [M+H]$^+$ 1984.0119, found 1983.9865.

BODIPY$_1$-HN-NWYFIVF-COOH (BODIPY$_1$-Pep$_5$). Yield: 63 %. Red powder. Analytic HPLC: Gradient A, retention time: 46.4 min, purity: 97.7 %. HRMS(MALDI-TOF): calc. for $C_{78}H_{90}BF_2N_{13}NaO_{13}^+$ [M+Na]$^+$ 1488.6804, found 1488.6771; calc. for $C_{78}H_{90}BF_2KN_{13}O_{13}^+$ [M+K]$^+$ 1504.6544, found 1504.6543.

BODIPY$_1$-HN-DEHYFIVF-COOH (BODIPY$_1$-Pep$_6$). Yield: 31 %. Red powder. Analytic HPLC: Gradient A, retention time: 35.8 min, purity: 95.2 %. HRMS(MALDI-TOF): calc. for $C_{73}H_{85}BF_2N_{12}NaO_{15}^+$ $[M+Na]^+$ 1441.6281, found 1441.5949; calc. for $C_{73}H_{85}BF_2KN_{12}O_{15}^+$ $[M+K]^+$ 1457.6020, found 1457.5786.

DP$_1$-HN-Ahx-P-(pTyr)-LKTK-COOH (BODIPY$_1$-Pep$_7$). Yield: 50 %. Red powder. Analytic HPLC: Gradient B, retention time: 19.3 min, purity: 95.9 %. HRMS(MALDI-TOF): calc. for $C_{62}H_{89}BFN_{11}O_{14}P^+$ $[M-F]^+$ 1272.6470, found 1272.7580.

BODIPY$_1$-HN-DRVYIHPF-CONH$_2$ (BODIPY$_1$-Pep$_{10}$). Yield: 37 %. Red powder. Analytic HPLC: Gradient B, retention time: 19.3 min, purity: 95.9 %. HRMS(MALDI-TOF): calc. for $C_{70}H_{91}BF_2N_{16}O_{12}^+$ $[M+H]^+$ 1396.7128, found 1396.6222.

Biological experiments

Cell Culture Conditions. Nasopharyngeal carcinoma (NPC) cell line C666 and Cervical carcinoma cell line HeLa were used in this study. C666 and HeLa were cultivated in Roswell Park Memorial Institute Medium (RPMI 1640) and Dulbecco's Modified Eagle Medium (DMEM) containing 10% v/v Fetal Bovine Serum (FBS) and 1% v/v Penicillin Streptomycin respectively. Both C666 and HeLa were maintained at 37 ^{o}C and 5% CO_2.

Dark Cytotoxicity Assay. The dark cytotoxicity and were assessed by MTT viability assay. Cells (6 x 10^3 per well) were seeded onto 96-well plates and then incubated at 37 ^{o}C with 5% CO_2 in dark for 24 h prior to the addition of samples. The cells were then incubated with samples for another 24 h. Medium were then removed, and the cell monolayers were washed with 1X PBS and then incubated with 100 mL medium with 5% v/v MTT solution (5 mg/mL) at 37 ^{o}C for 2.5 h. 80 mL of solution were removed and 100 mL Dimethyl sulfoxide (DMSO) were added to dissolve the formazan crystals. The absorbance of the formazan crystal was measured at 540 nm and 650 nm by dual-wavelength Azure microplate reader after 1 h of shaking.

Light Cytotoxicity Assay. The light cytotoxicity of samples was assessed with MTT viability assay. The procedures of the MTT viability assay were previously described. Cells were irradiated with optical dose of 5J/cm^2 after 24 h incubation with sample and the medium were replaced with fresh medium prior to irradiation.

***In vitro* imaging and co-staining.** Cell were incubated with **BODIPY$_1$-Pep$_4$** (10mM) for 24h and then co-stained with Hoechst 33342 nuclear dye for 15 minutes. Imaging was performed by a Nikon Eclipse Ti2 confocal laser-scanning microscope.

Expression and purification of EBNA1 (a.a. 468-607). The gene construct for N-terminally hexahistidine tagged SMT3-EBNA1 DNA binding domain (residues 468-607) fusion protein was chemically synthesized and cloned into pET28a (+) vector (Genscript). The plasmid was transformed into *Escherichia coli* BL21 (DE3) competent cells and grown in 2YT media supplemented with 50 μg/ml kanamycin at 37 ˚C. When the OD_{600} of the cells reached 0.8, expression was induced with 0.5 mM isopropyl β-D-1-thiogalactopyranoside (IPTG) and the cells were grown at 25 ˚C overnight. The cells were harvested by centrifugation at 5000 rpm (6,238 x *g*) for 10 mins at 4 ˚C. The following protein purification protocol was adapted from a previously published report[3]. The cell pellet was resuspended in lysis buffer (20 mM Tris, pH 8.0, 500 mM NaCl, 10 mM imidazole, 5% (v/v) glycerol, 5 mM β-mercaptoethanol (BME), 17.4 μg/ml phenylmethylsulfonyl fluoride (PMSF) and 1 mM $MgCl_2$). The resuspended cells were lysed by sonication and the lysate was separated from the insoluble fractions by centrifugation at 24,000 rpm (69,673 x *g*) for 30 min at 4 ˚C. The protein was purified by nickel affinity using a His-trap column (GE Healthcare) equilibrated with lysis buffer. The lysate was first loaded onto the His-trap column, then washed with 10 column volumes of wash buffer (20 mM Tris pH 8.0, 500 mM NaCl ,30 mM imidazole, 5% glycerol and 5 mM BME) and the protein was eluted with 3 - 4 column volumes of elution buffer (20 mM Tris pH 8.0, 500 mM NaCl, 300 mM imidazole, 5% glycerol and 5 mM BME). The eluted protein was treated with ULP1 to cleave the hexahistidine-SMT3 protein tag. Any residual uncleaved fusion protein and the histidine-tagged ULP1 protease were removed by nickel affinity while the tag-free EBNA1 (468-607) protein was recovered by flowing wash buffer through the His-trap column (GE Healthcare). The fractions were pooled, concentrated and further purified by size exclusion chromatography using a Superdex 200 Increase 10/300 column (GE Healthcare), equilibrated with a buffer containing 1 mM HEPES, pH 7.2, 500

mM NaCl and 10 mM dithiothreitol (DTT). The relevant protein fractions were analysed using SDS-PAGE, pooled, concentrated to 6 mg/ml and stored at -80 ˚C.

Fluorescent titration. The EBNA1 protein was prepared as the procedure above, while Bovine serum albumin (BSA) protein was purchased from Thermo Fisher Scientific. Both BSA and EBNA1 were further prepared as solution (120 µM) in buffer (1 mM HEPES, pH 7.2, 500 mM NaCl and 10 mM dithiothreitol). The protein solution was gradually added into **BODIPY$_1$-Pep$_4$** (2000 µL 1.2 µM in same buffer) by pipette. Each time after addition of protein, the resulting solution was shaken slowly for around 2 min, then the fluorescent spectrum was recorded. The experiment was stopped when the influence on fluorescence was saturated. The increased of volume is less than 4% in total.

Reference:

[1] M. Kondo, S. Furukawa, K. Hirai, S. Kitagawa, *Angew. Chemie - Int. Ed.* **2010**, *49*, 5327–5330.

[2] S. Eissler, M. Kley, D. Bächle, G. Loidl, T. Meier, D. Samson, *J. Pept. Sci.* **2017**, *23*, 757–762.

[3] T. E. Messick, G. R. Smith, S. S. Soldan, M. E. McDonnell, J. S. Deakyne, K. A. Malecka, L. Tolvinski, P. A. J. van den Heuvel, B.-W. Gu, J. A. Cassel, D. H. Tran, B. R. Wassermann, Y. Zhang, V. Velvadapu, E. R. Zartler, P. Busson, A. B. Reitz, P. M. Lieberman, *Sci. Transl. Med.* **2019**, *11*, eaau5612.

NMR spectra of products

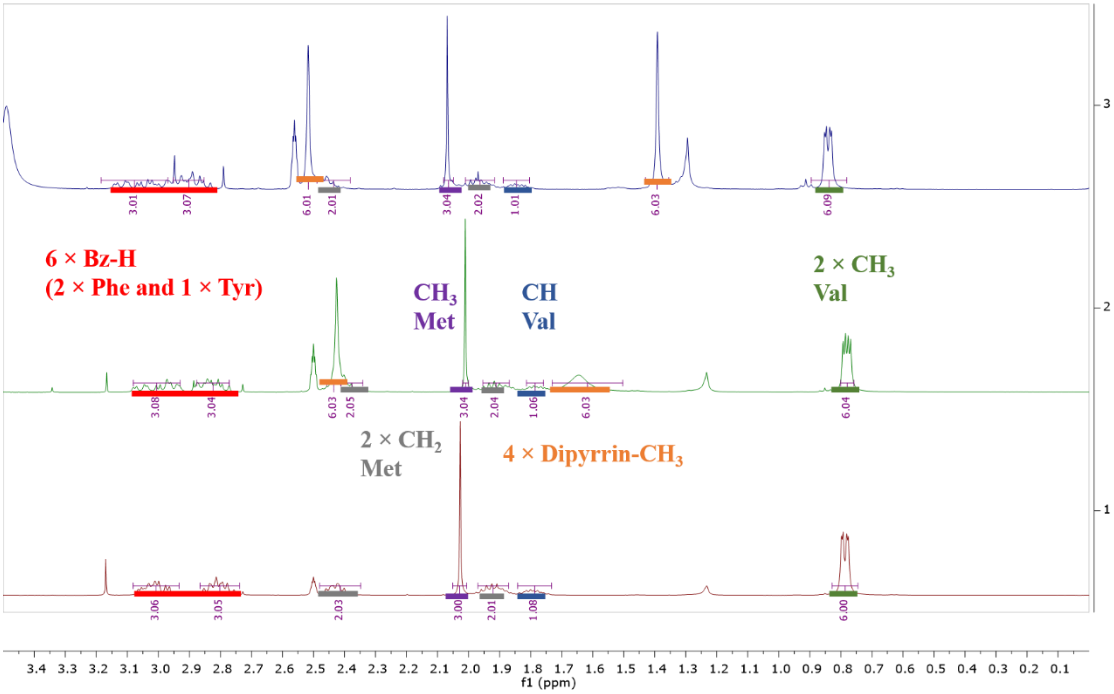

Fig. S8. Comparison and assignment of ^{1}H-NMR for **Pep$_1$** (bottom), **DP$_1$-Pep$_1$** (middle) and **BODIPY$_1$-Pep$_1$** (top) in range 3.5~0.0 ppm. Signal of side chain sp^3 C–H and methyl groups on dipyrrin/BODIPY.

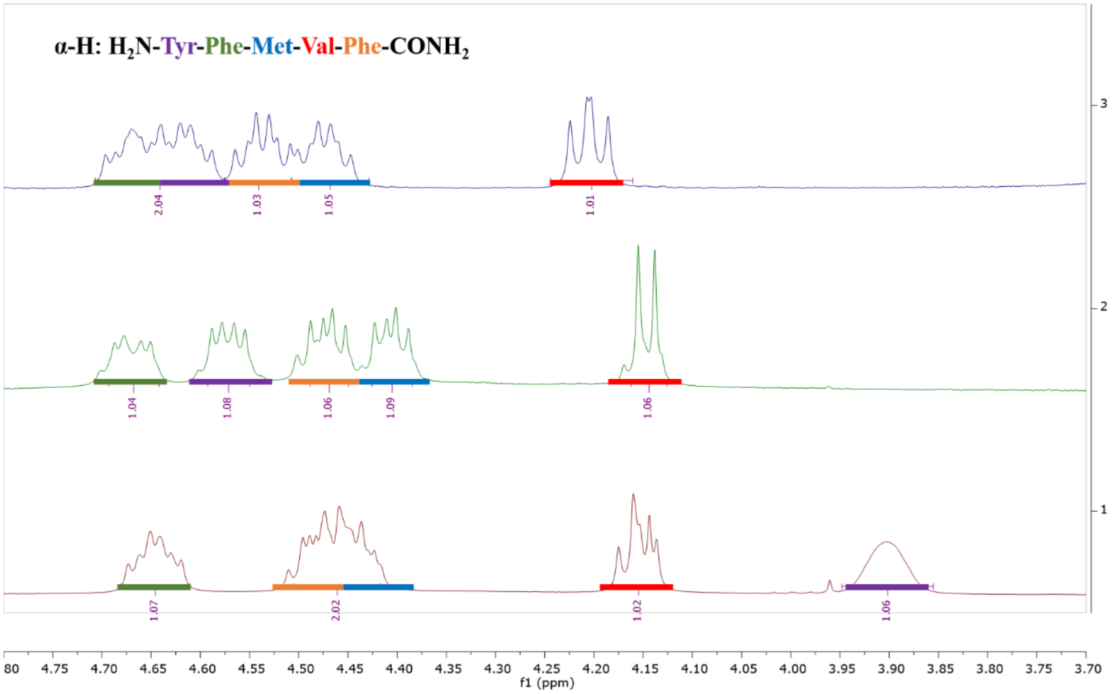

Fig. S9. Comparison and assignment of ^{1}H-NMR for **Pep$_1$** (bottom), **DP$_1$-Pep$_1$** (middle) and **BODIPY$_1$-Pep$_1$** (top) in range 4.8~3.7 ppm. Signal of α-H on the backbone of peptide.

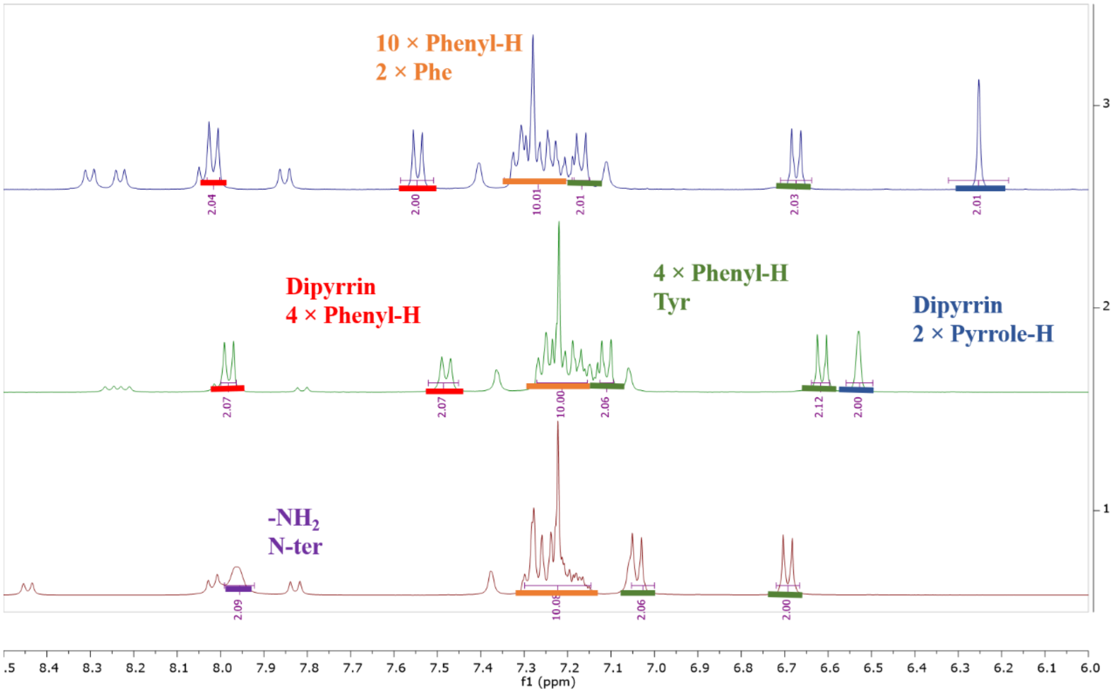

Fig. S10. Comparison and assignment of ^{1}H-NMR for **Pep$_1$** (bottom), **DP$_1$-Pep$_1$** (middle) and **BODIPY$_1$-Pep$_1$** (top) in range 9.0~6.0 ppm. Signal of aryl proton from Phe, Tyr and dipyrrin/BODIPY, as well as amide proton from backbone of peptide.

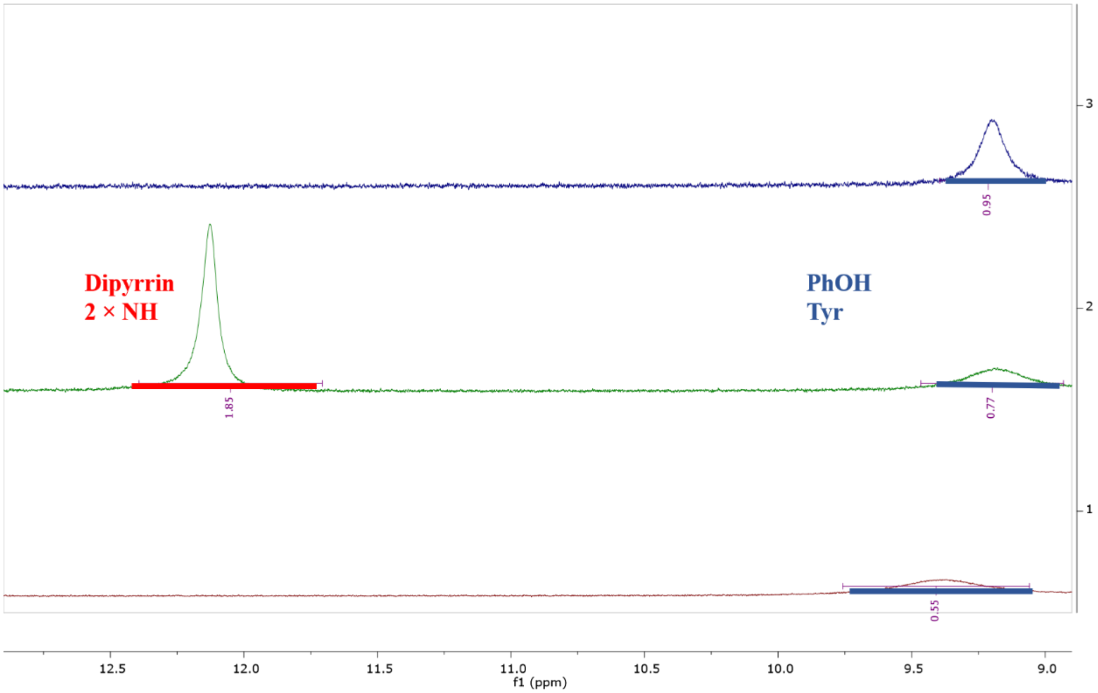

Fig. S11. Comparison and assignment of ^{1}H-NMR for **Pep$_1$** (bottom), **DP$_1$-Pep$_1$** (middle) and **BODIPY$_1$-Pep$_1$** (top) in range 12.9~8.9 ppm. Signal of acidic proton from Tyr (phenol-H) and dipyrrin (NH).

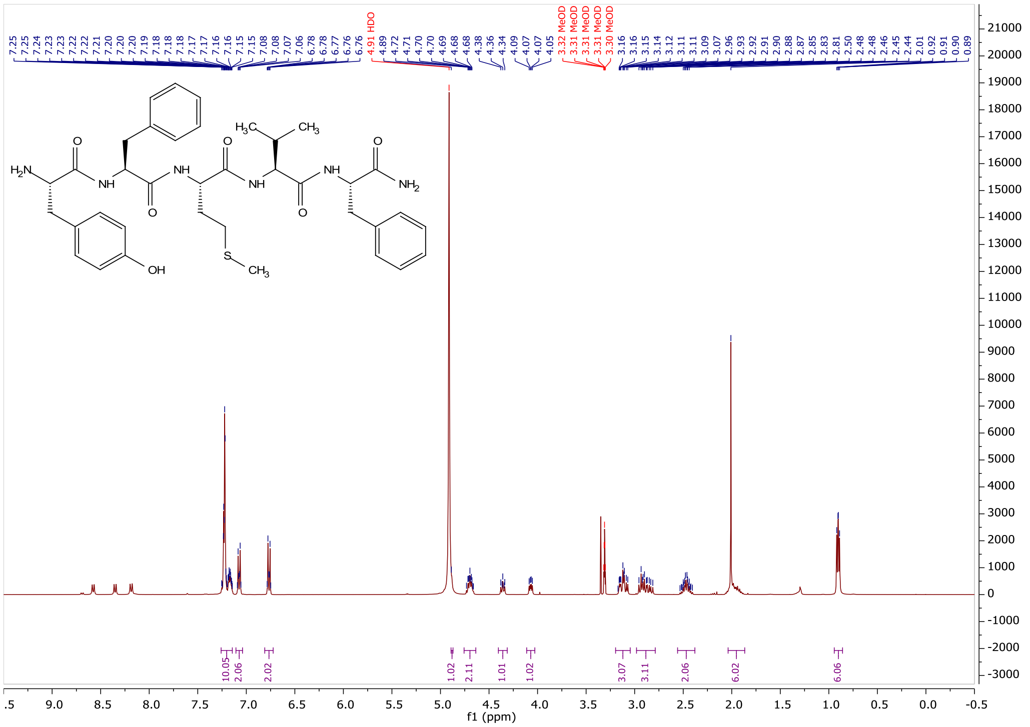

^{1}H-NMR spectrum of **Pep$_1$** in methanol-d_4.

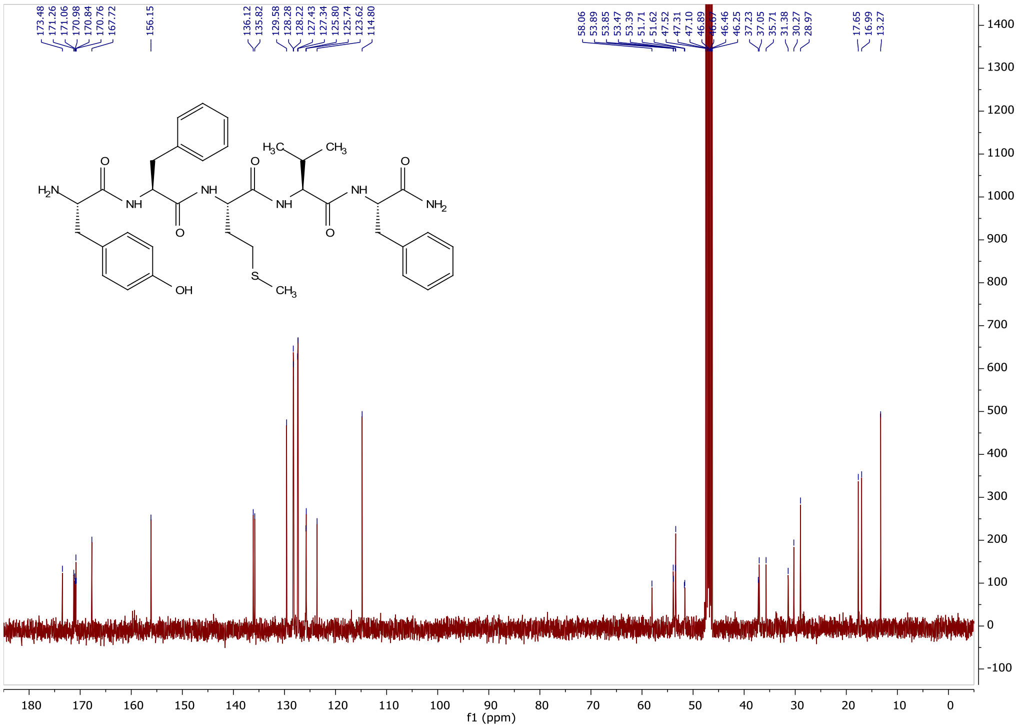

^{13}C-NMR spectrum of **Pep$_1$** in methanol-d_4.

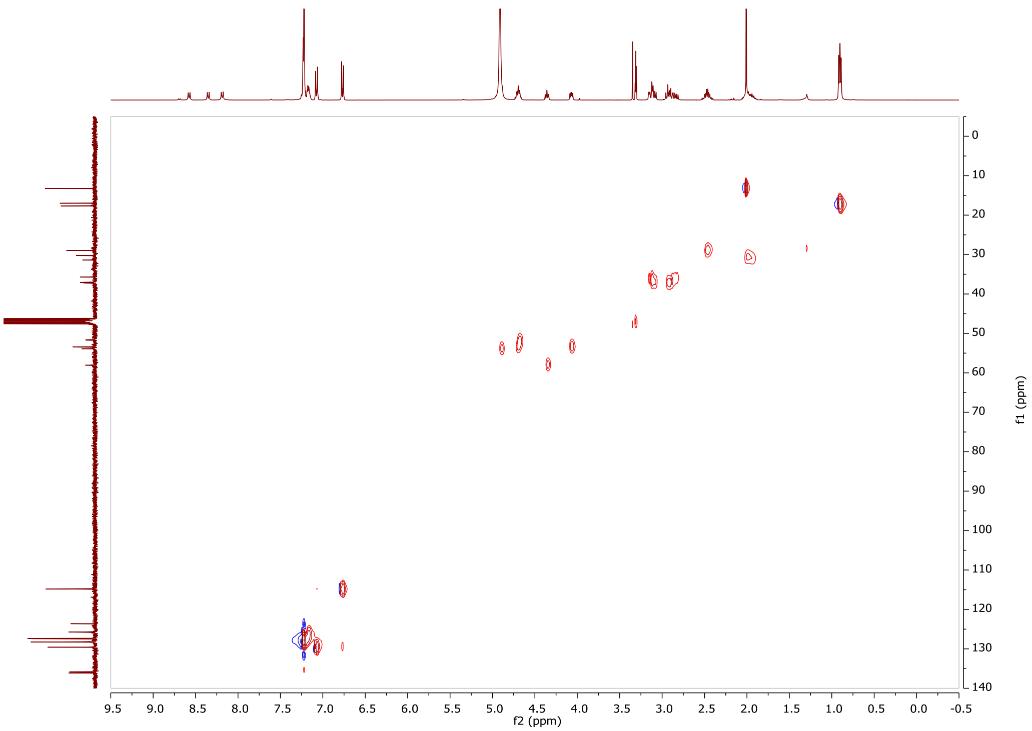

HSQC spectrum of **Pep₁** in methanol-*d*₄.

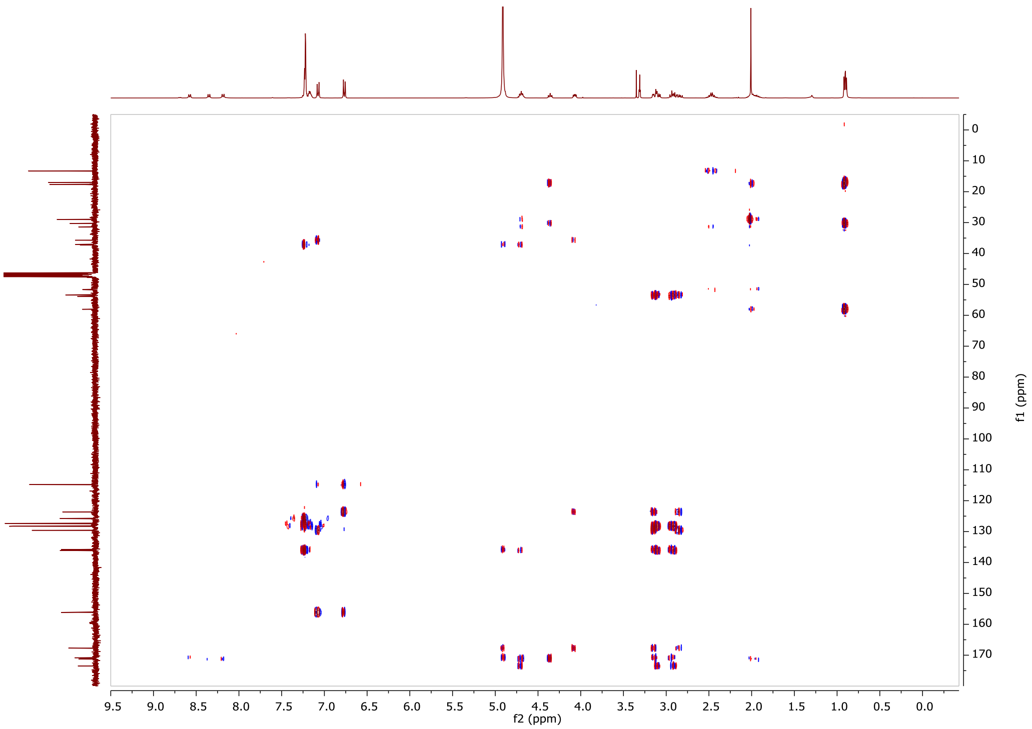

HMBC spectrum of **Pep₁** in methanol-*d*₄.

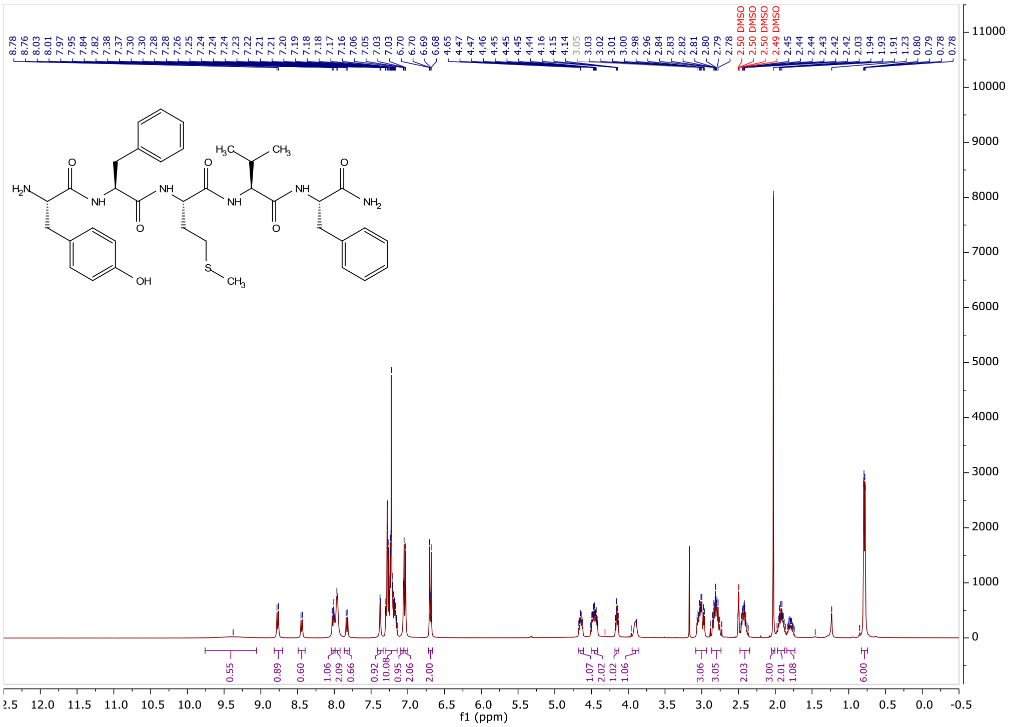

^{1}H-NMR spectrum of **Pep₁** in DMSO-*d*₆**.**

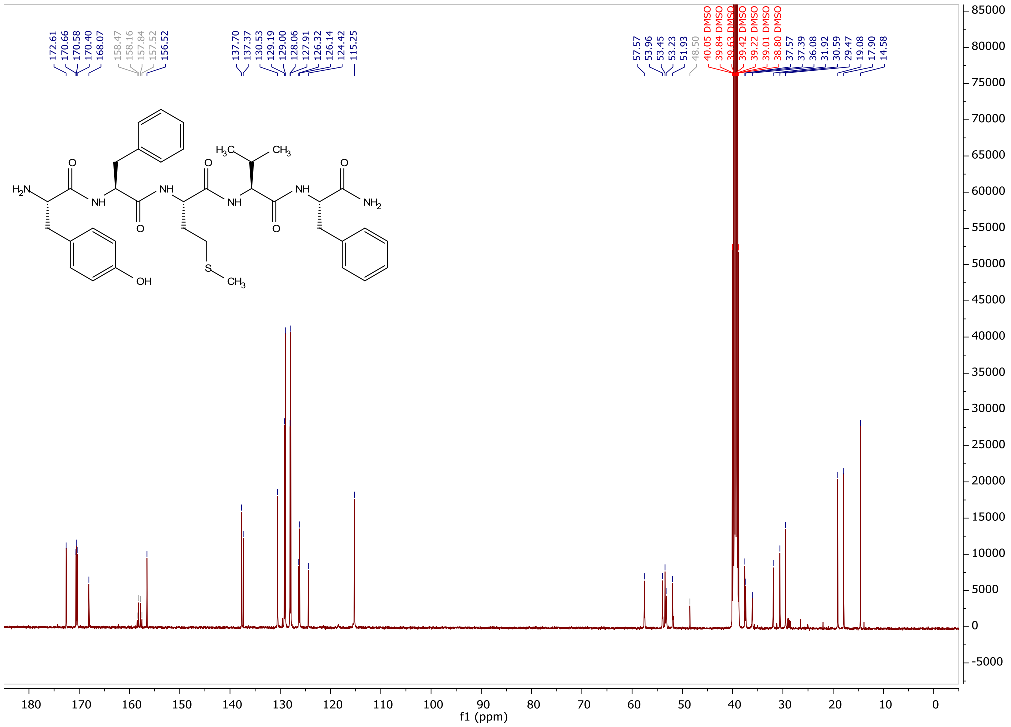

^{13}C-NMR spectrum of **Pep₁** in DMSO-*d*₆**.**

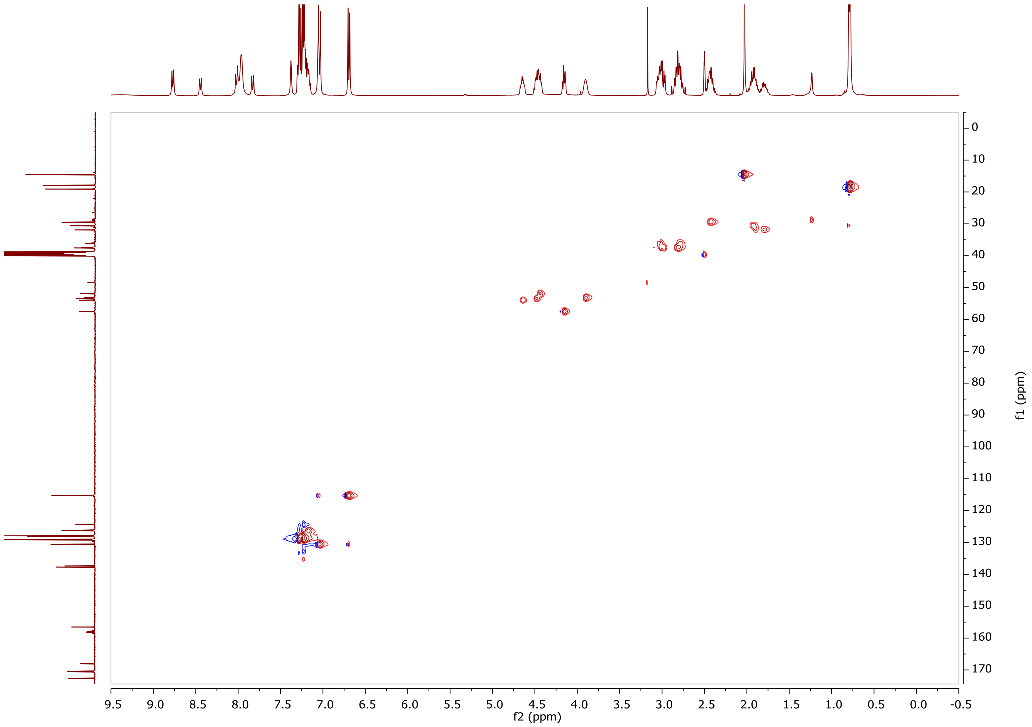

HSQC spectrum of **Pep₁** in DMSO-d_6.

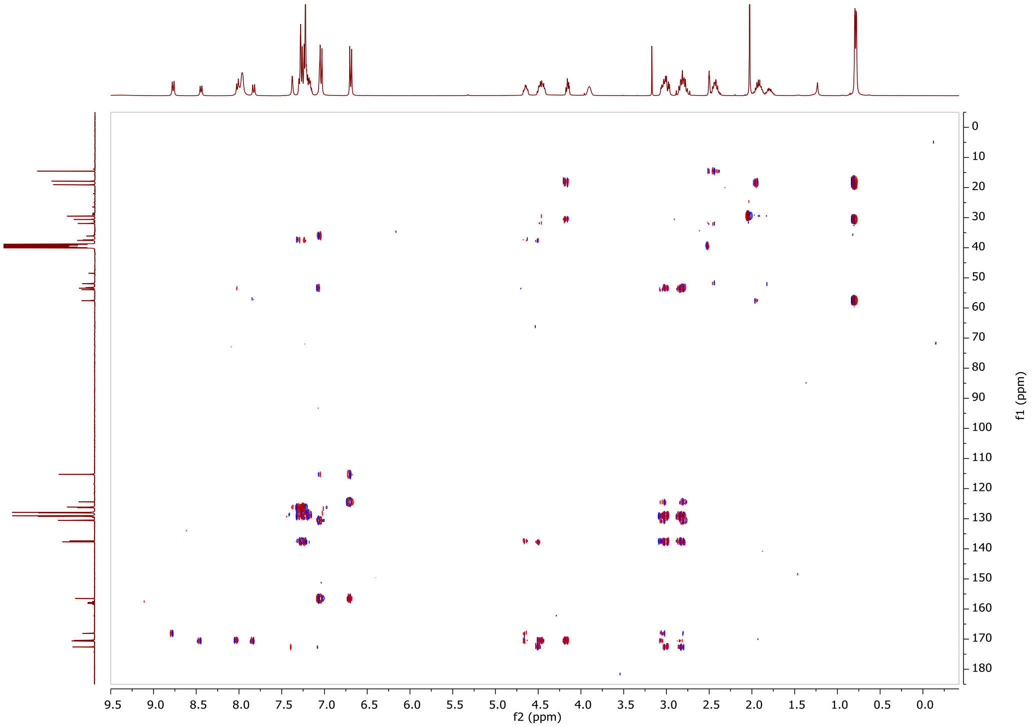

HMBC spectrum of **Pep₁** in DMSO-d_6.

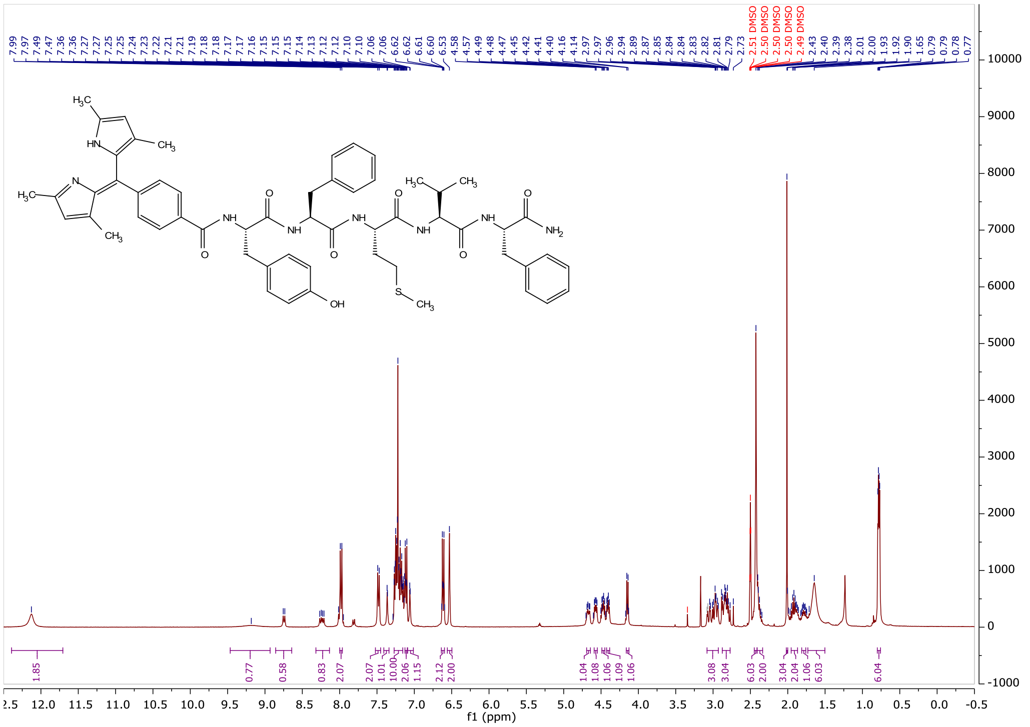

^{1}H-NMR spectrum of **DP₁-Pep₁** in DMSO-*d₆*.

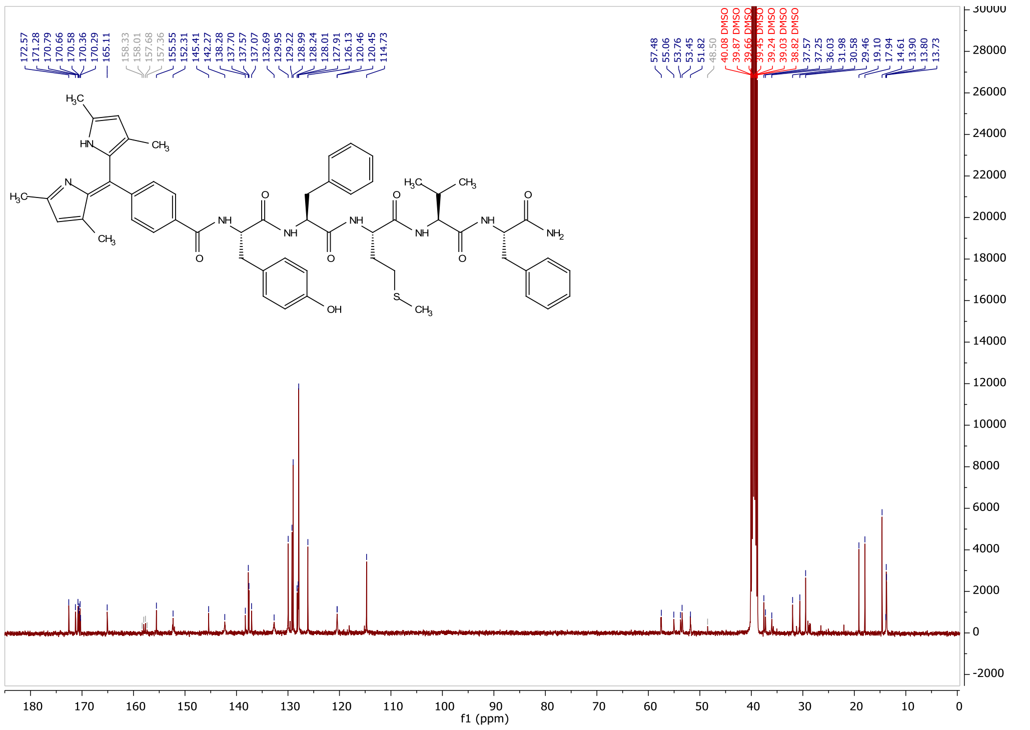

^{13}C-NMR spectrum of **DP₁-Pep₁** in DMSO-*d₆*.

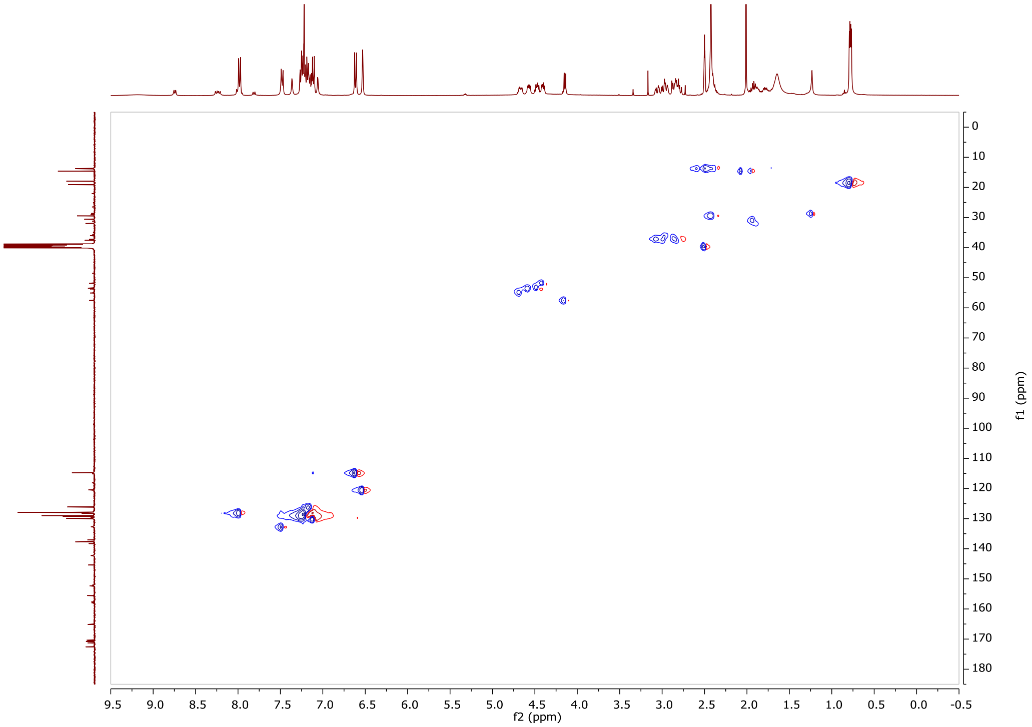

HSQC spectrum of **DP$_1$-Pep$_1$** in DMSO-d_6**.**

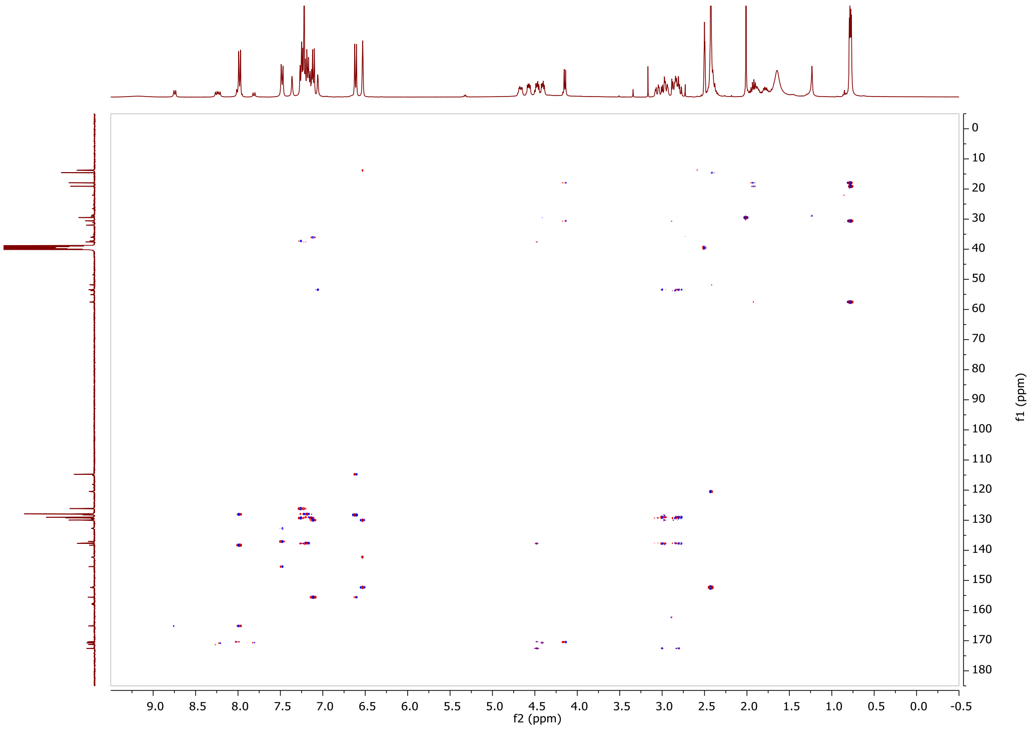

HMBC spectrum of **DP$_1$-Pep$_1$** in DMSO-d_6**.**

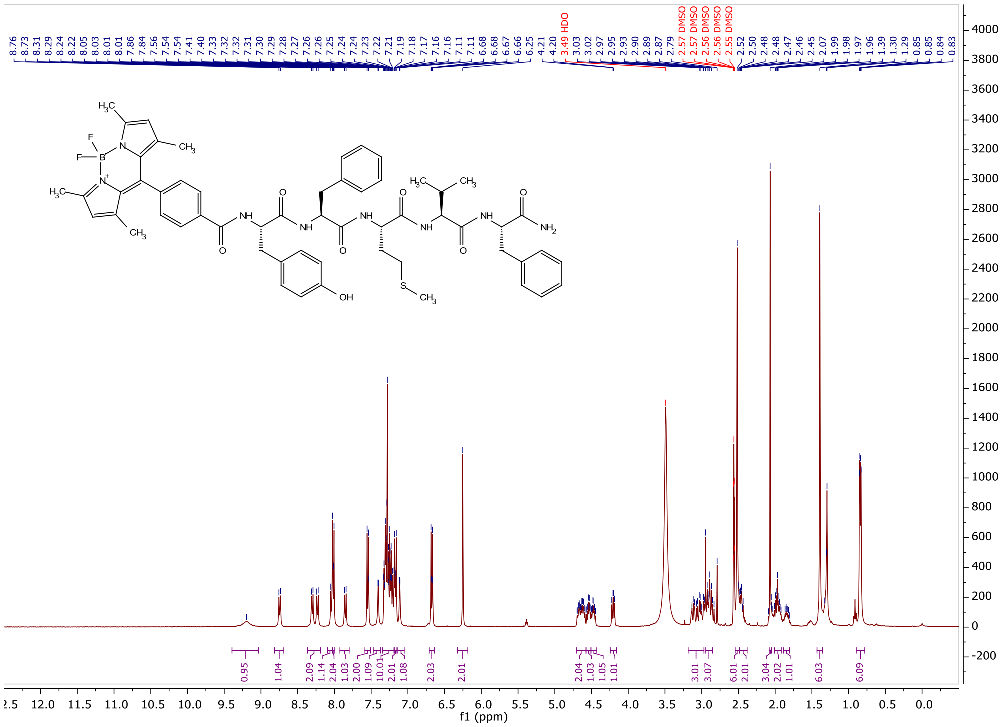

^{1}H-NMR spectrum of **BODIPY$_1$-Pep$_1$** in DMSO-d_6.

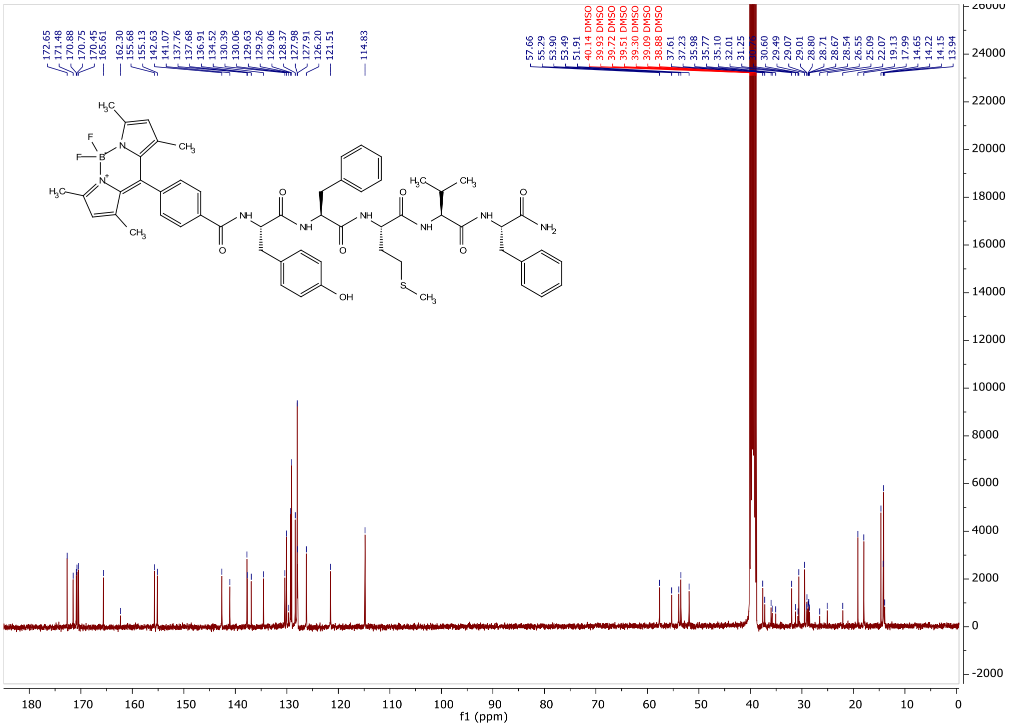

^{13}C-NMR spectrum of **BODIPY$_1$-Pep$_1$** in DMSO-d_6.

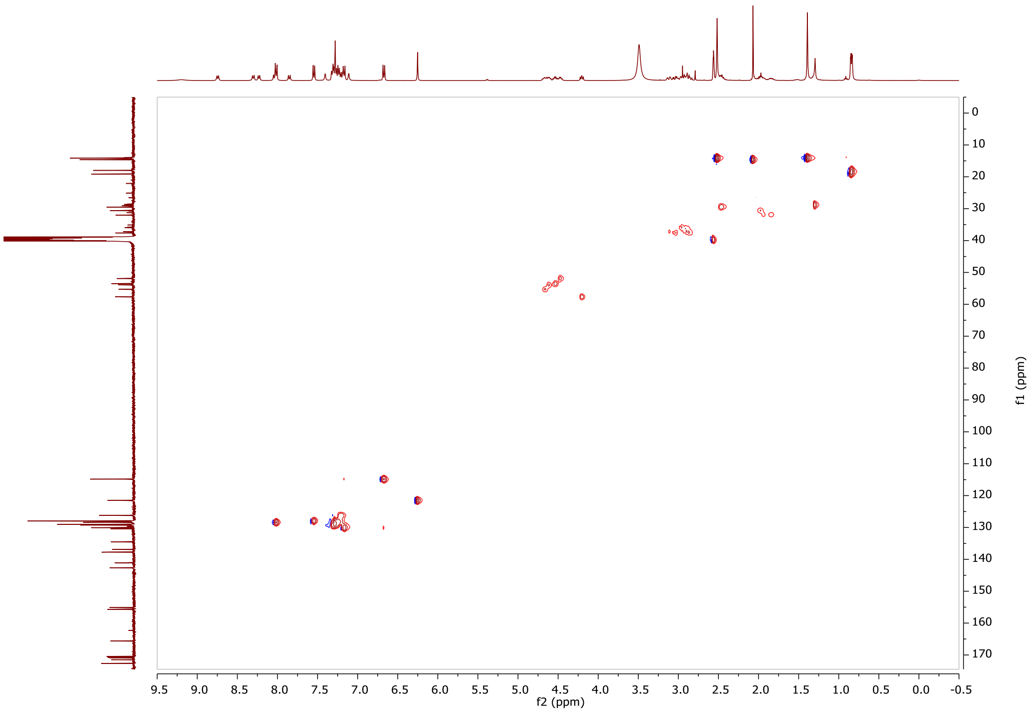

HSQC spectrum of **BODIPY$_1$-Pep$_1$** in DMSO-d_6.

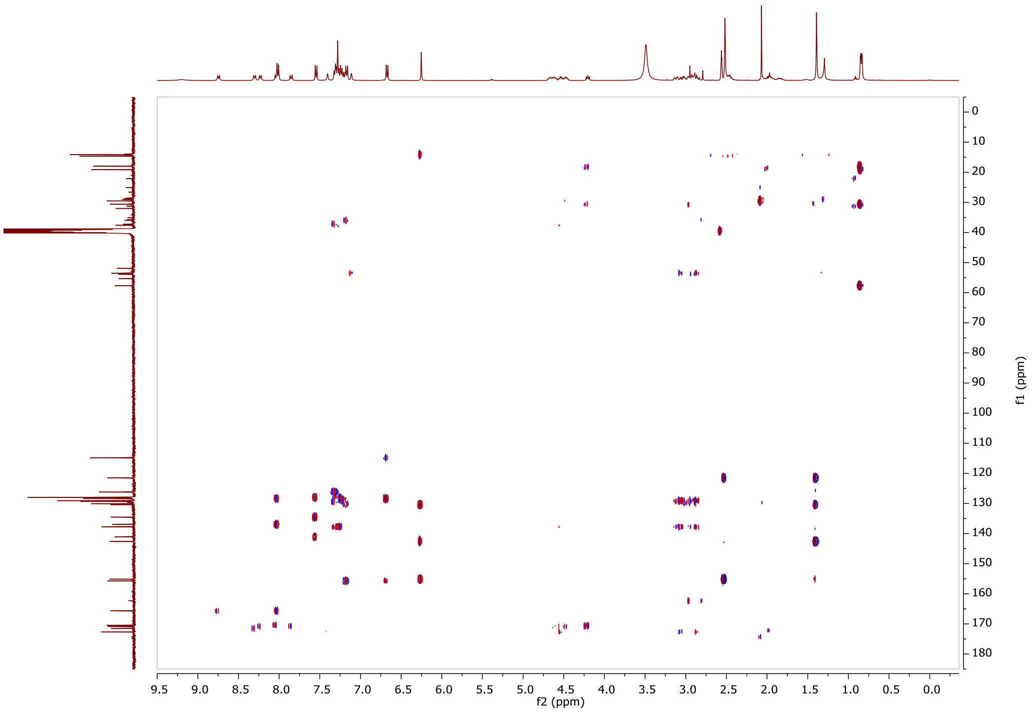

HMBC spectrum of **BODIPY$_1$-Pep$_1$** in DMSO-d_6.

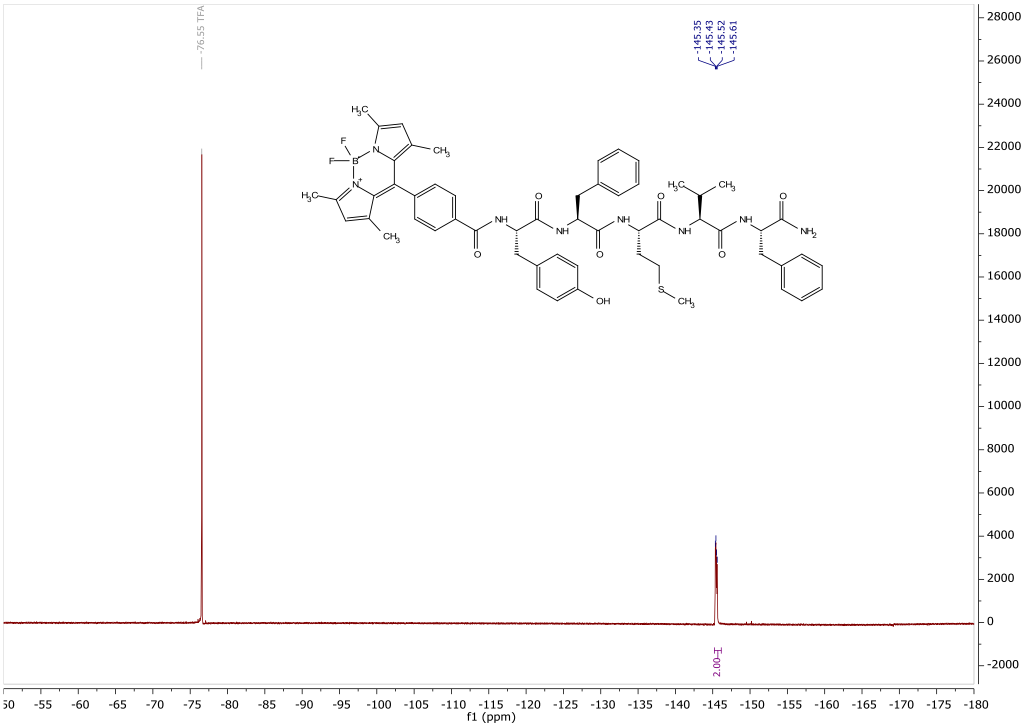

^{19}F-NMR spectrum of **BODIPY$_1$-Pep$_1$** in DMSO-d_6.

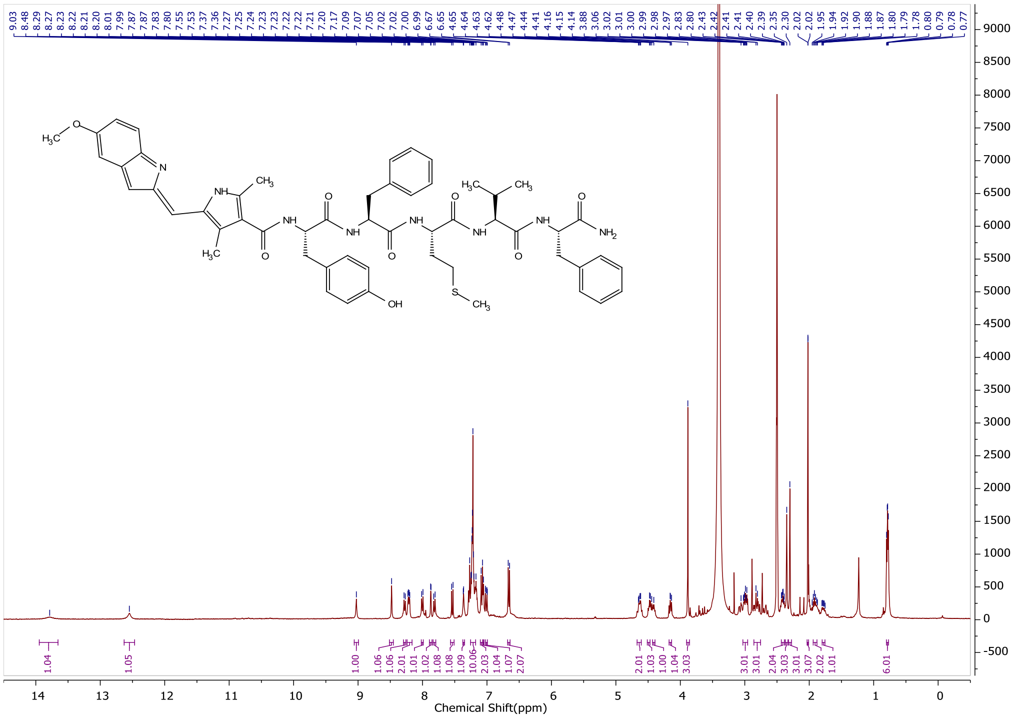

^{1}H-NMR spectrum of **DP$_{16}$-Pep$_1$** in DMSO-d_6.

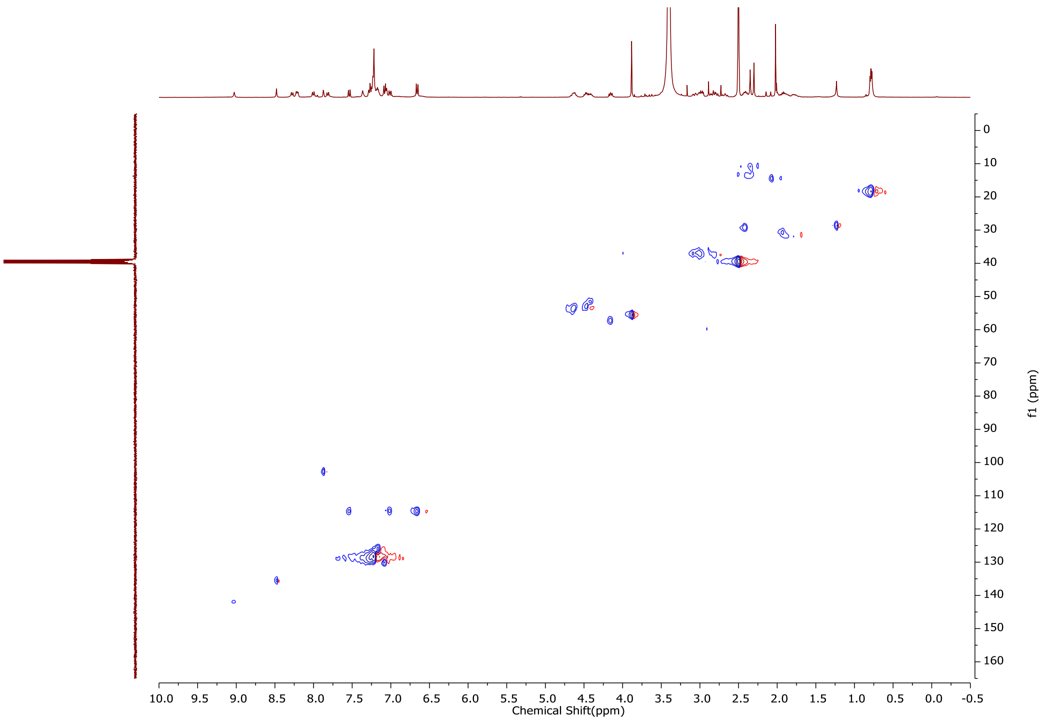

HSQC spectrum of **DP$_{16}$-Pep$_1$** in DMSO-d_6.

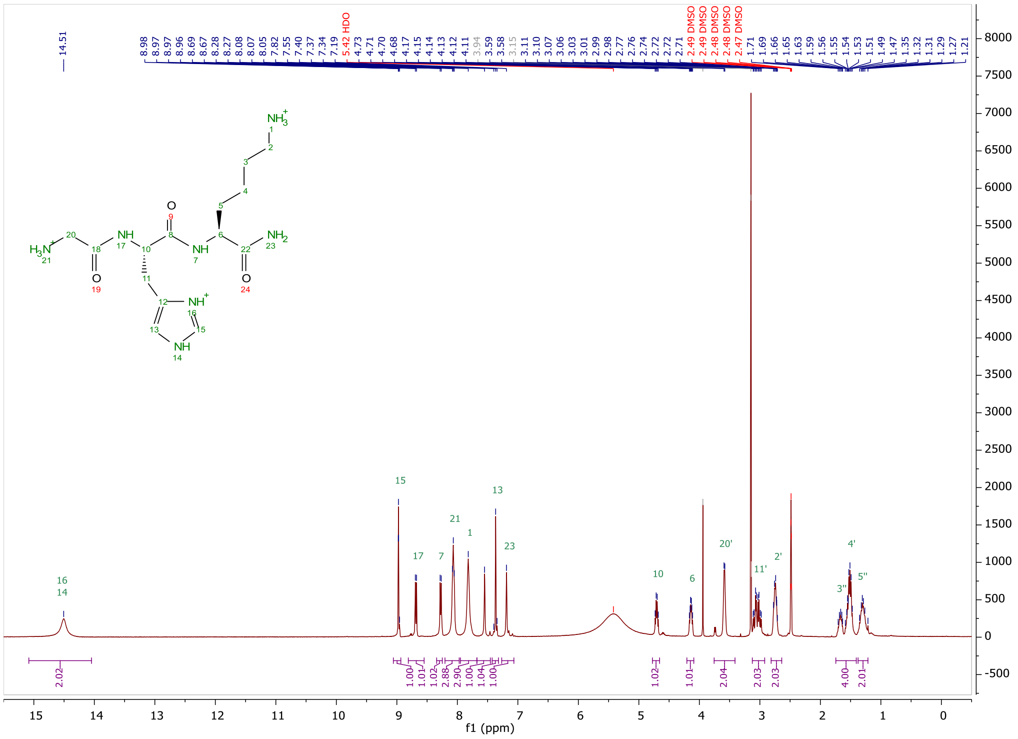

^{1}H-NMR spectrum of **Pep$_{11}$** in DMSO-d_6.

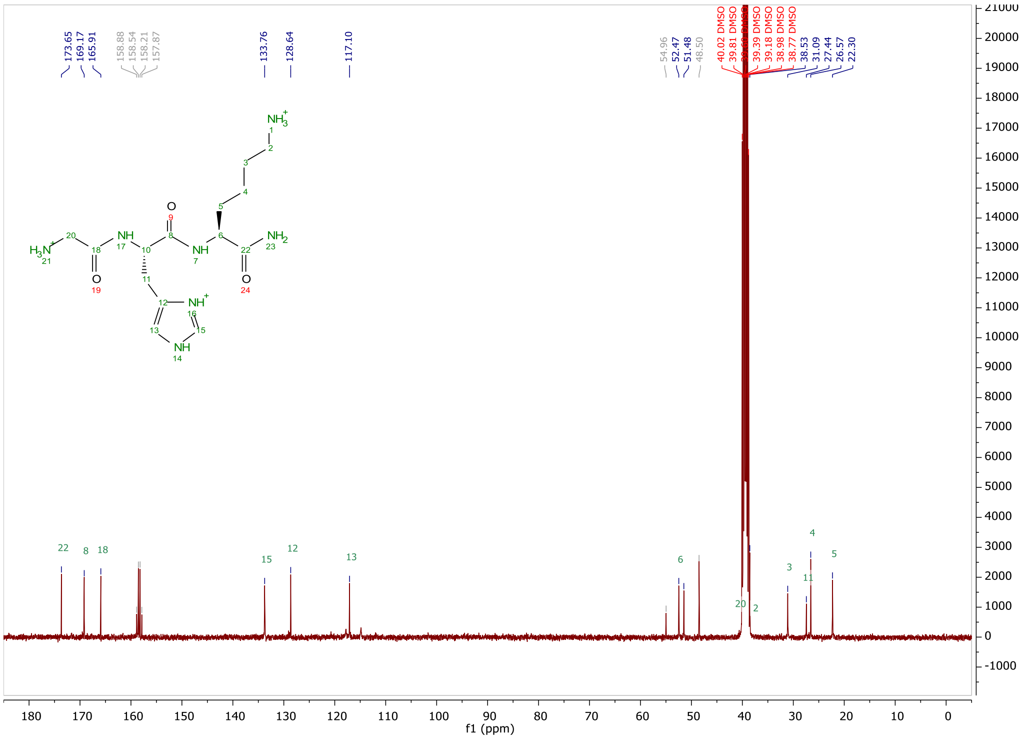

^{13}C-NMR spectrum of **Pep$_{11}$** in DMSO-d_6.

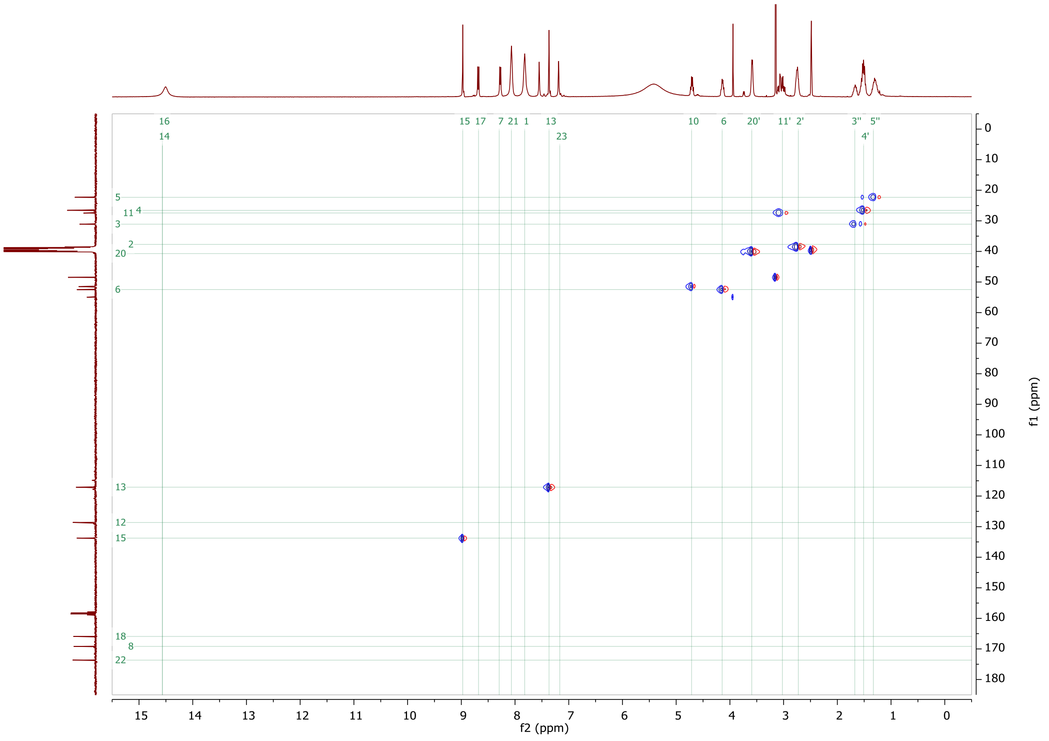

HSQC spectrum of **Pep$_{11}$** in DMSO-d_6.

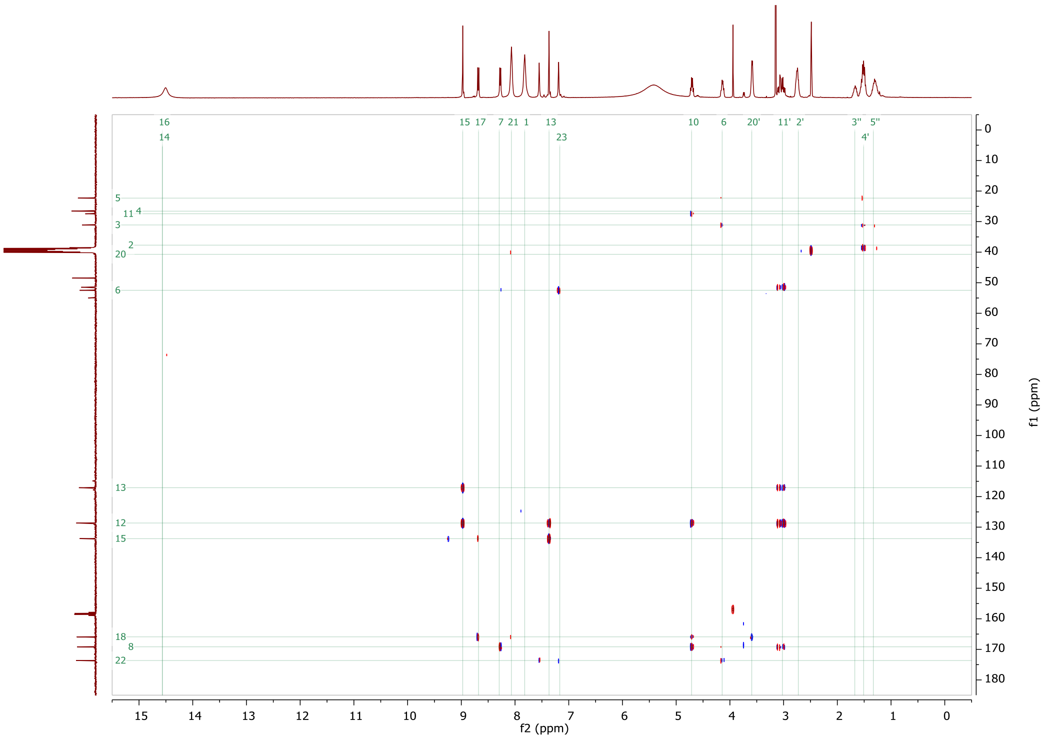

HMBC spectrum of **Pep$_{11}$** in DMSO-d_6.

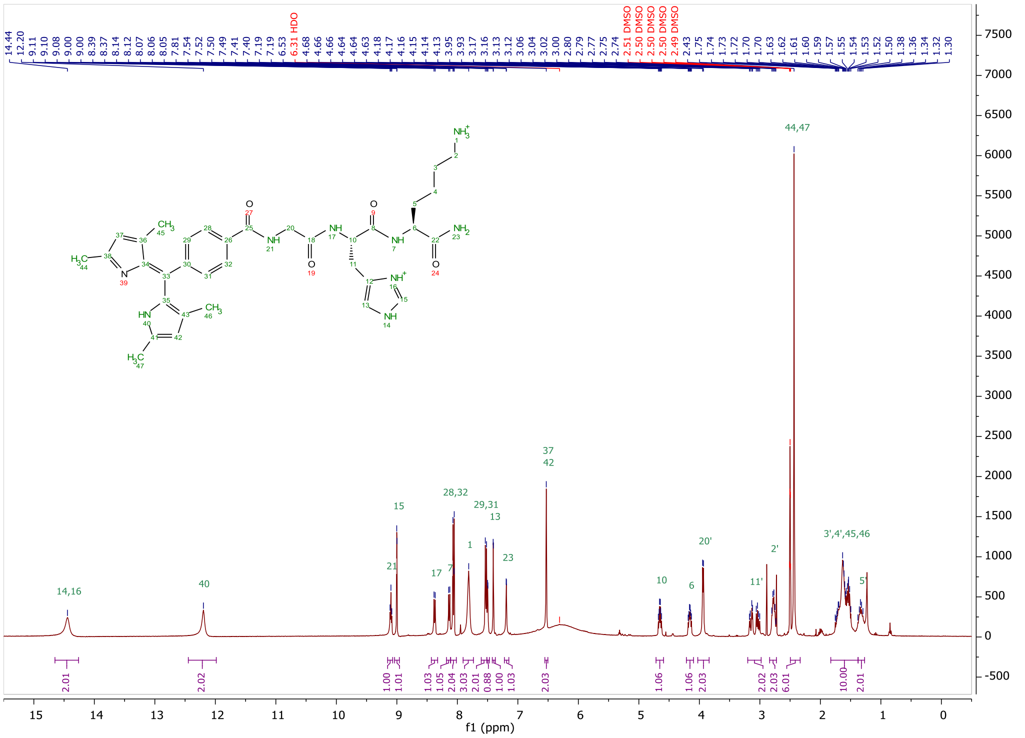

^{1}H-NMR spectrum of **DP$_1$-Pep$_{11}$** in DMSO-d_6.

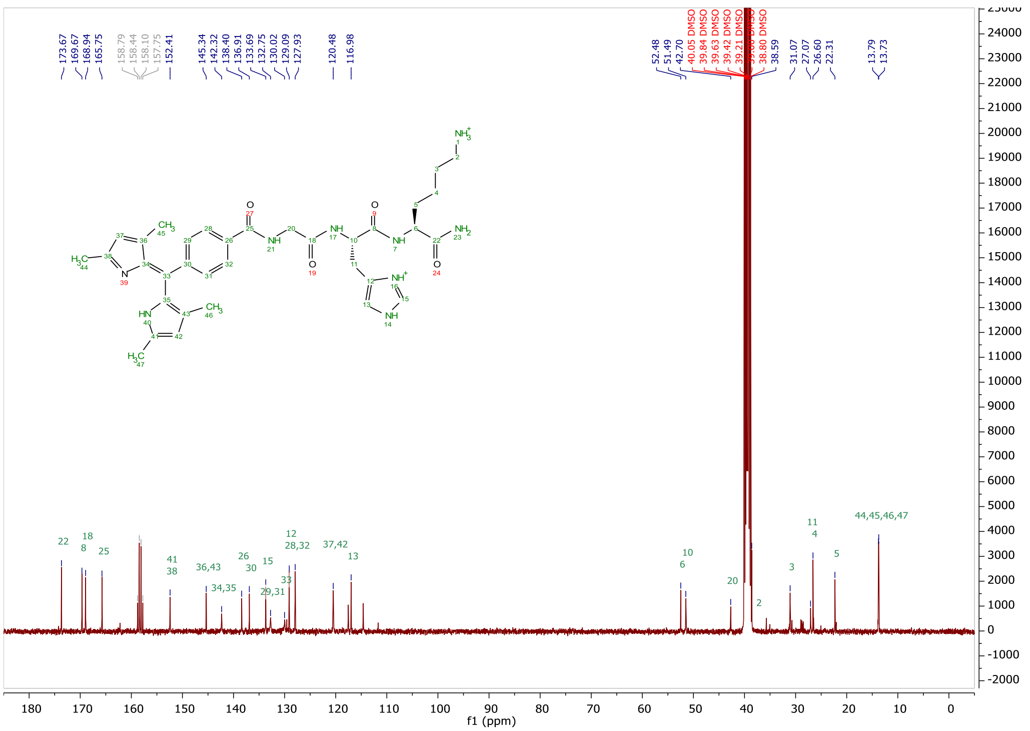

^{13}C-NMR spectrum of **DP$_1$-Pep$_{11}$** in DMSO-d_6.

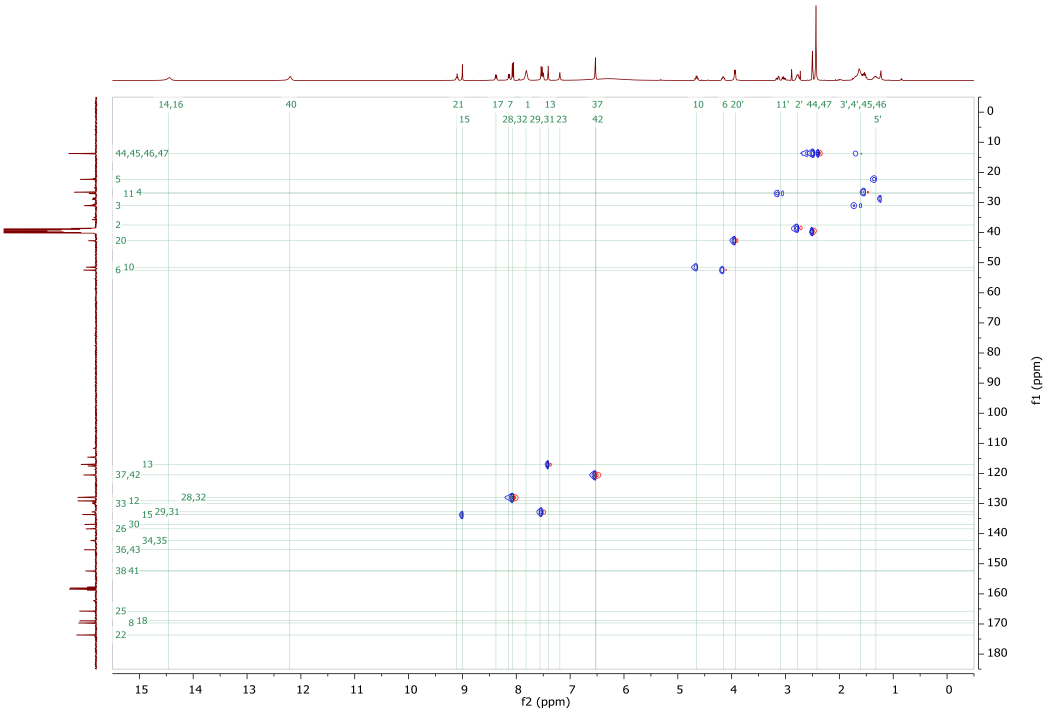

HSQC spectrum of **DP₁-Pep₁₁** in DMSO-d_6.

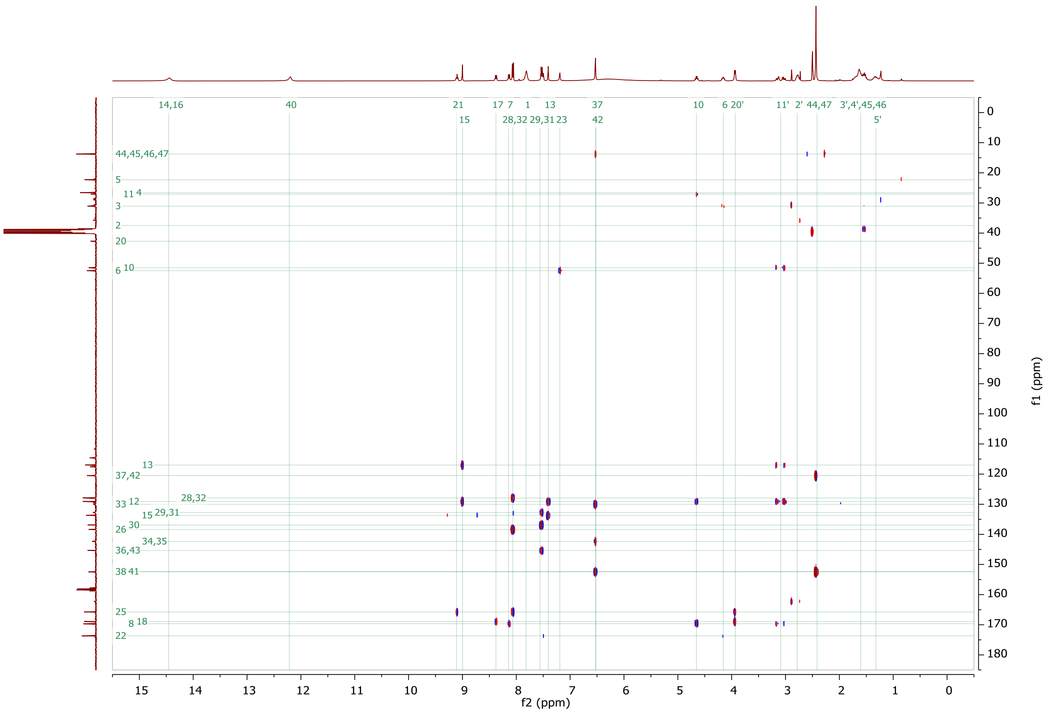

HMBC spectrum of **DP₁-Pep₁₁** in DMSO-d_6.

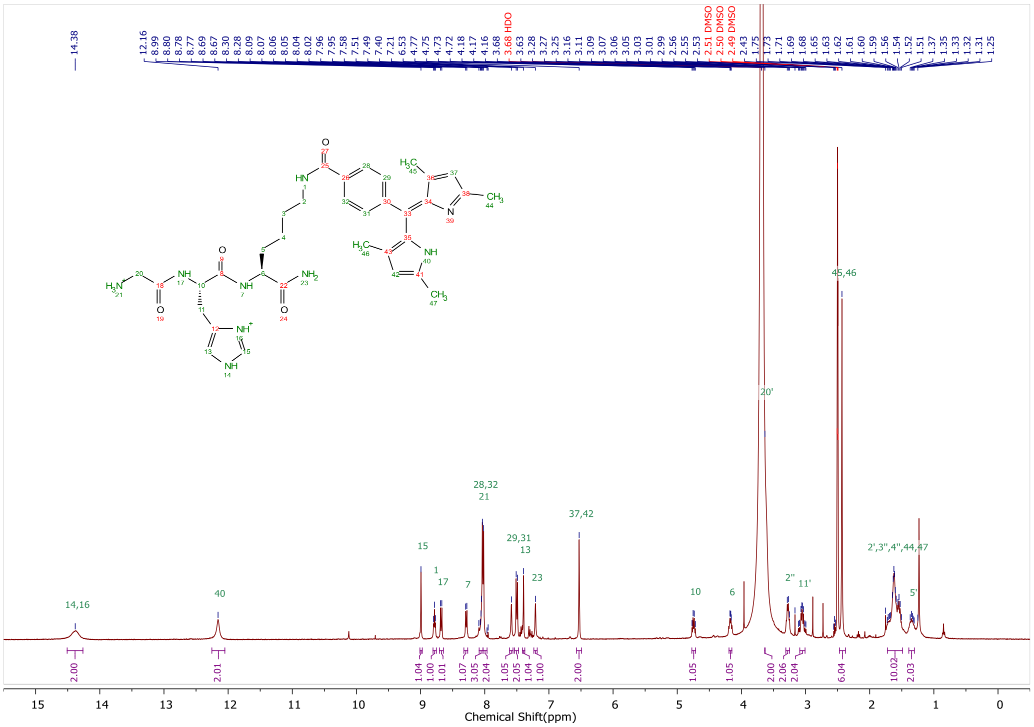

^{1}H-NMR spectrum of **DP₁-Pep₁₁*** in DMSO-d_6**.**

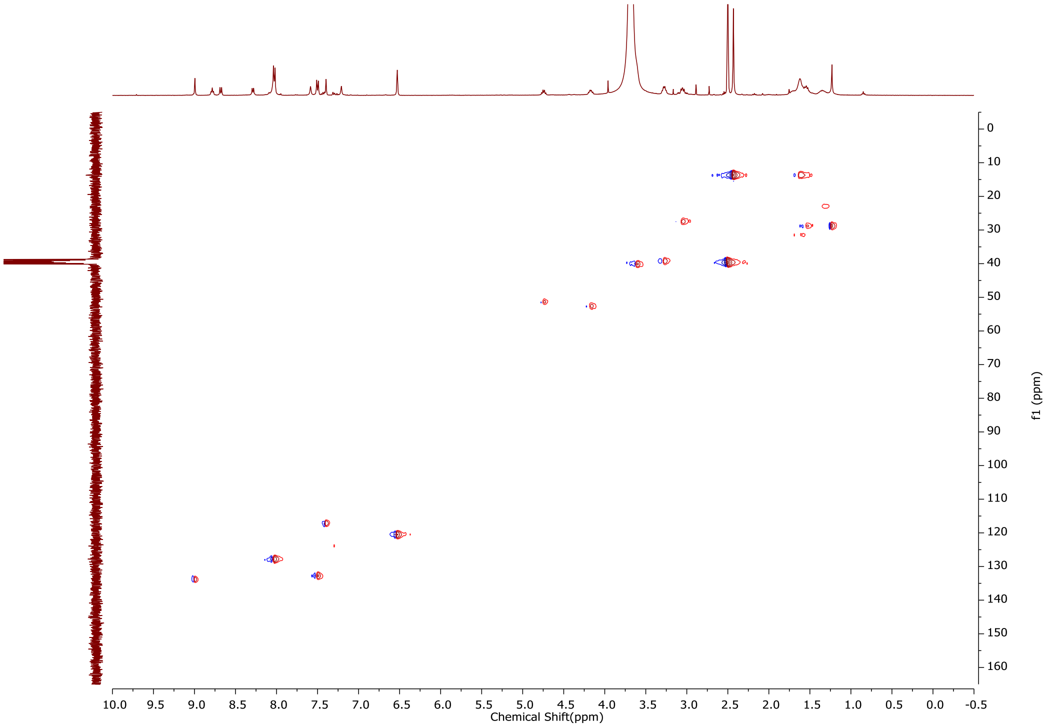

HSQC spectrum of **DP₁-Pep₁₁*** in DMSO-d_6**.**

HPLC chromatographs and MALID-TOF HRMS spectra of products

Pep$_1$: H$_2$N-YFMVF-CONH$_2$

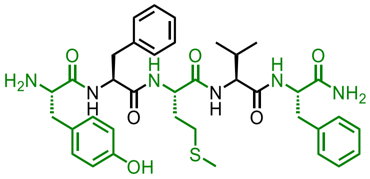

Chemical Formula: $C_{37}H_{48}N_6O_6S$
Exact Mass: 704.3356

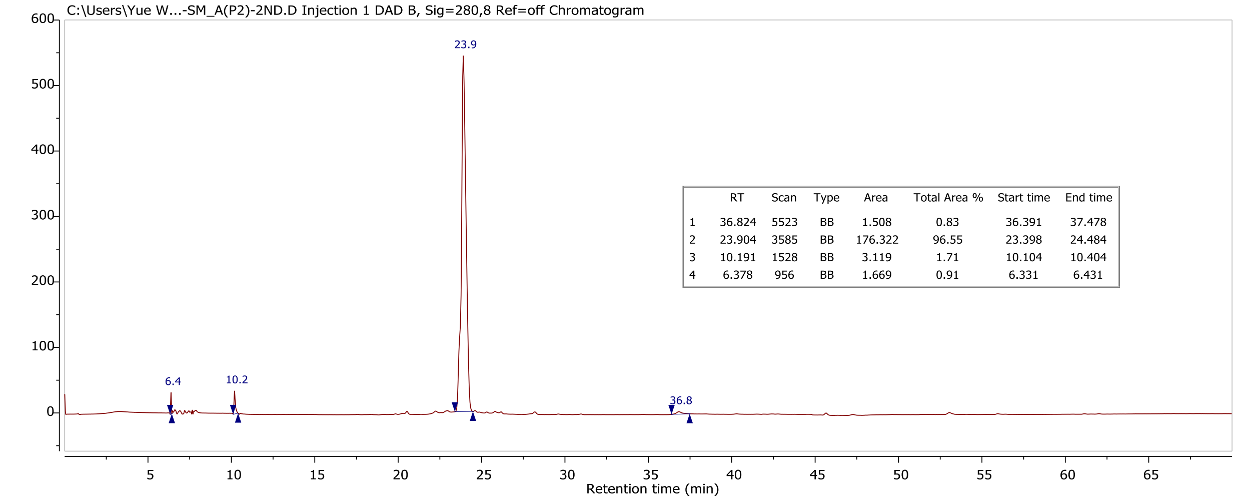

	RT	Scan	Type	Area	Total Area %	Start time	End time
1	36.824	5523	BB	1.508	0.83	36.391	37.478
2	23.904	3585	BB	176.322	96.55	23.398	24.484
3	10.191	1528	BB	3.119	1.71	10.104	10.404
4	6.378	956	BB	1.669	0.91	6.331	6.431

Analytic HPLC of **Pep$_1$**

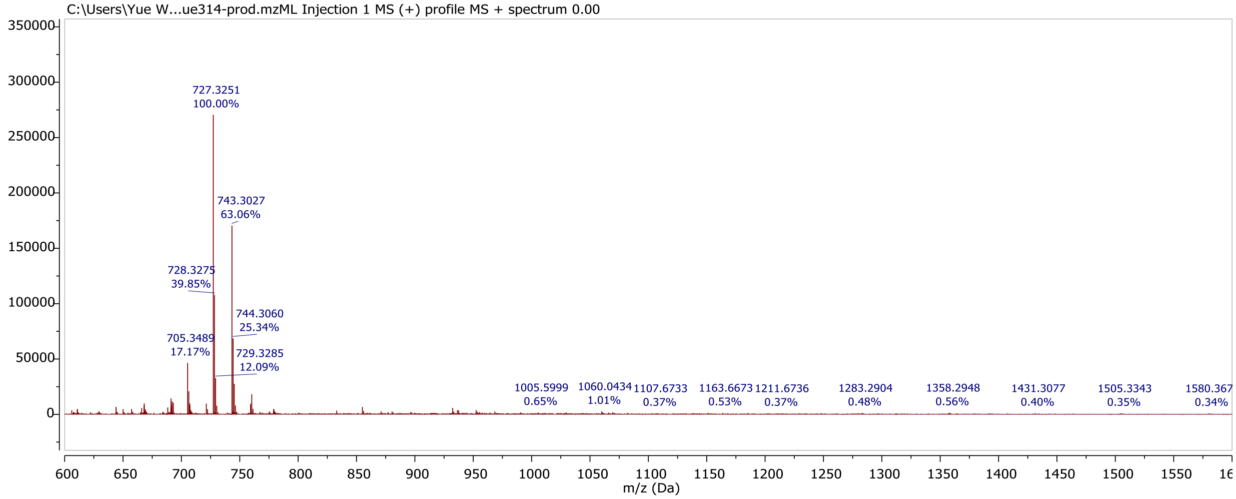

HRMS(MAIDL-TOF) of **Pep$_1$**

Pep$_2$: H$_2$N-Ahx-YFIVFGGKRPR-COOH

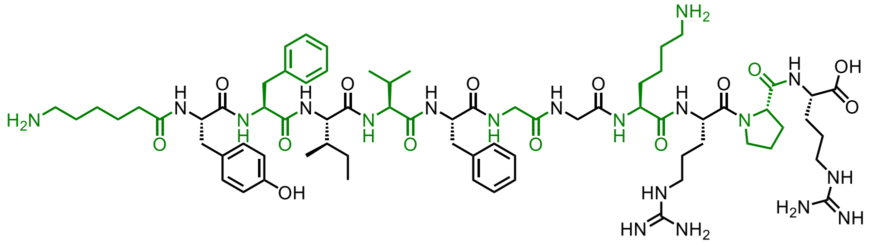

Chemical Formula: $C_{71}H_{109}N_{19}O_{14}$
Exact Mass: 1451.8401

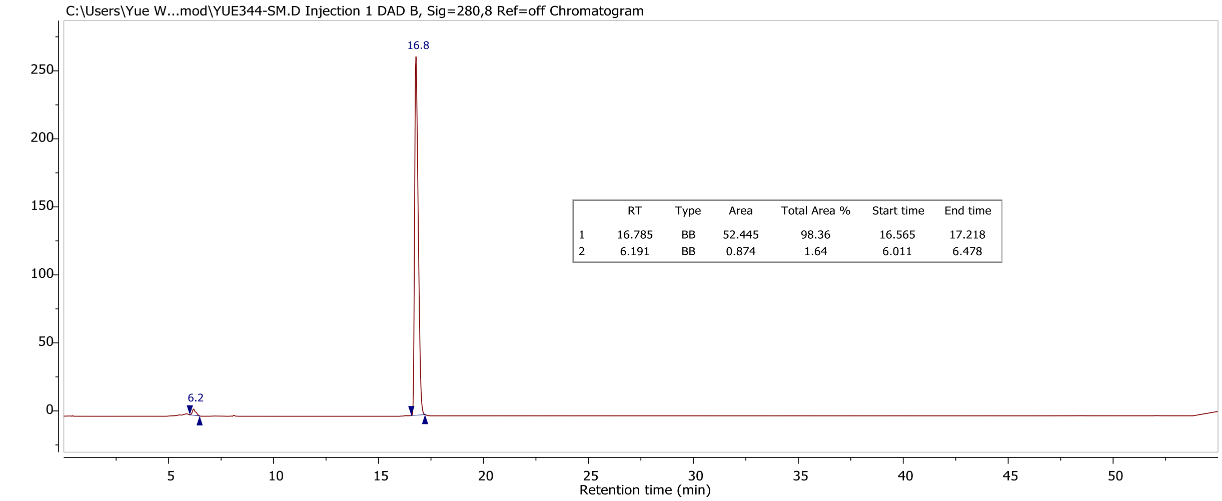

	RT	Type	Area	Total Area %	Start time	End time
1	16.785	BB	52.445	98.36	16.565	17.218
2	6.191	BB	0.874	1.64	6.011	6.478

Analytic HPLC of **Pep$_2$**

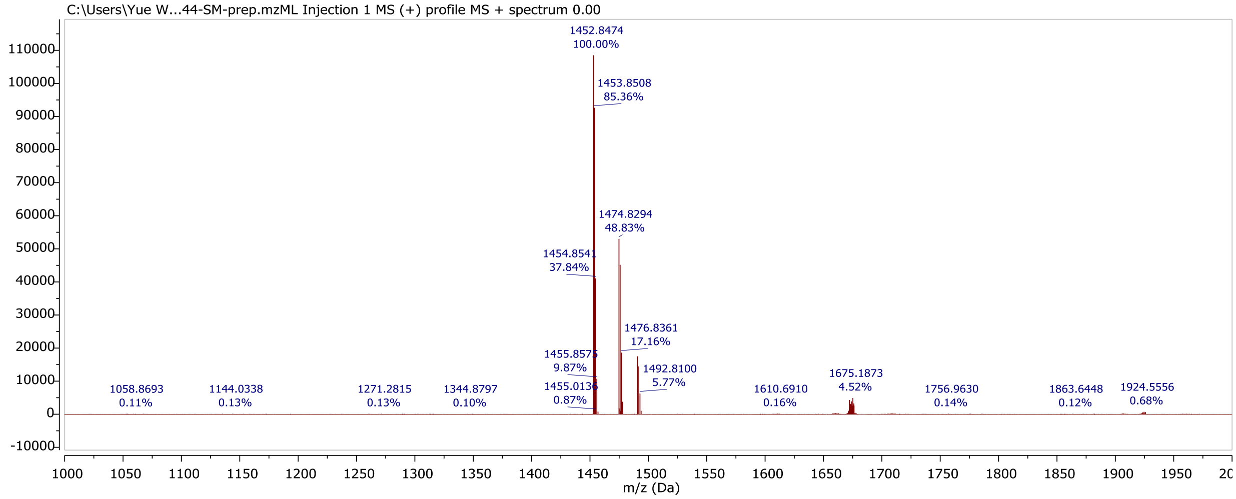

HRMS(MAIDL-TOF) of **Pep$_2$**

Pep$_3$: H_2N-C-Ahx- RrRK-GG-YFMVF-COOH

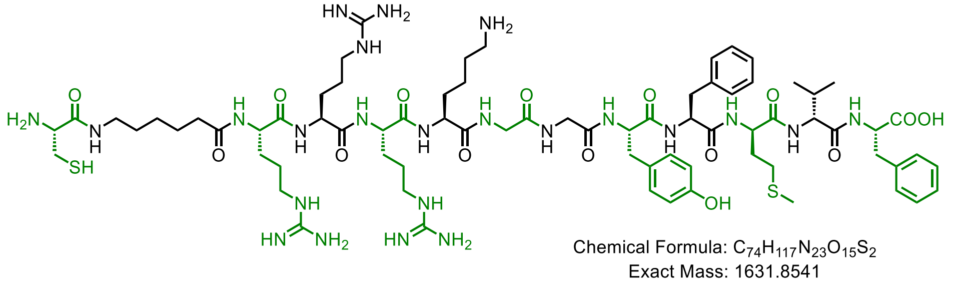

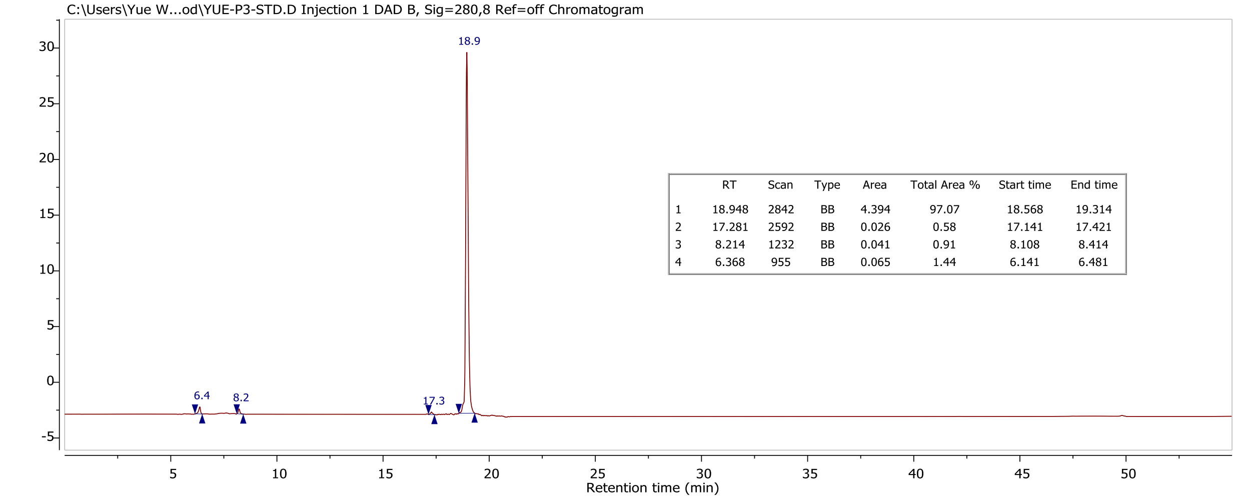

Analytic HPLC of **Pep$_3$**

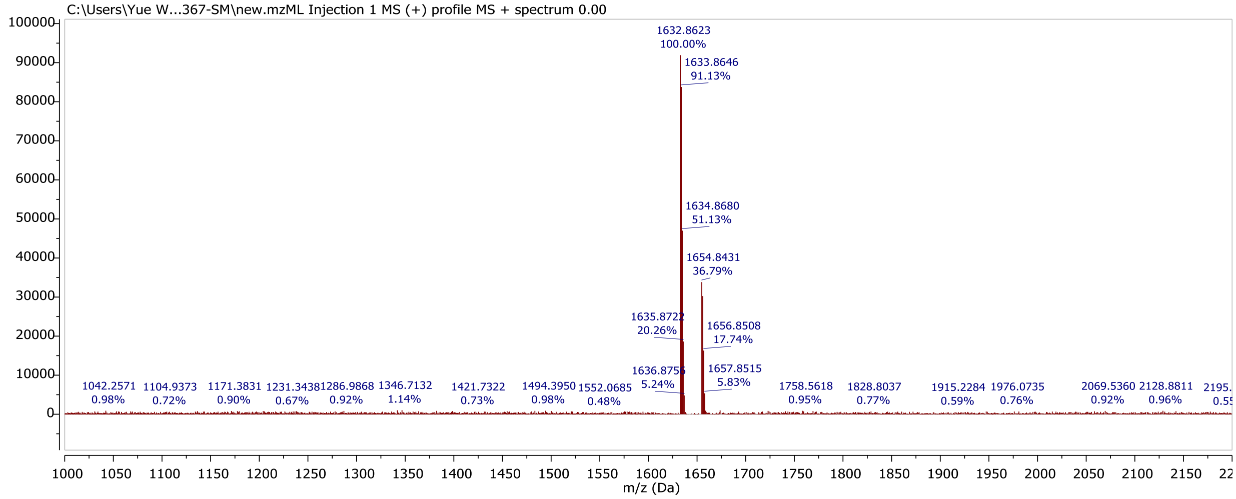

HRMS(MAIDL-TOF) of **Pep$_3$**

Pep$_4$: H_2N-C-Ahx-YFMVF-GG-RrRK-COOH

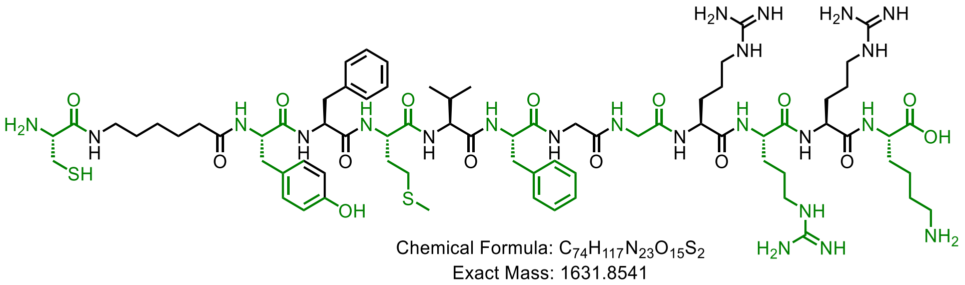

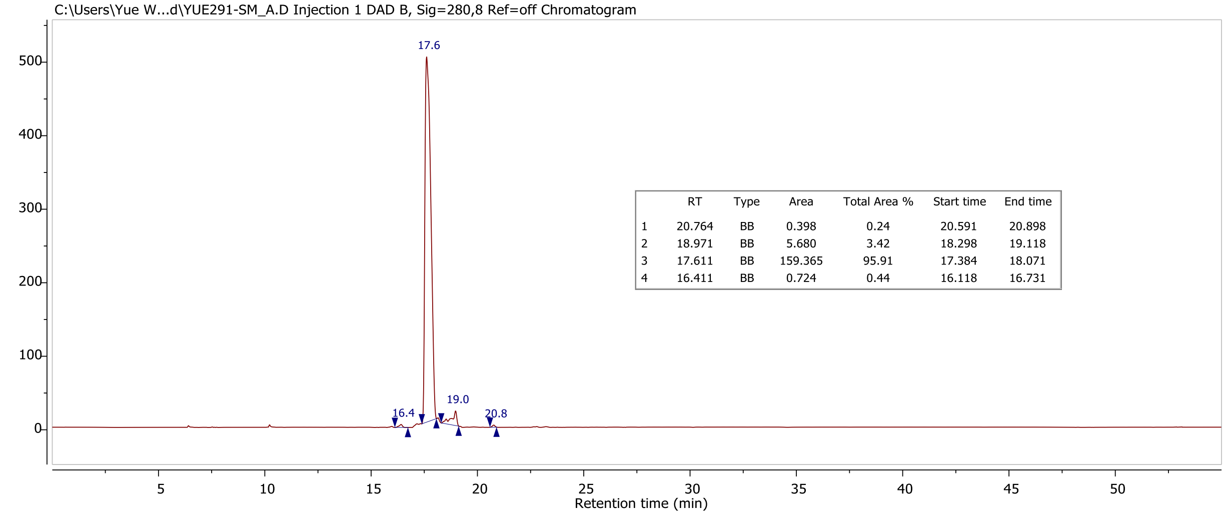

	RT	Type	Area	Total Area %	Start time	End time
1	20.764	BB	0.398	0.24	20.591	20.898
2	18.971	BB	5.680	3.42	18.298	19.118
3	17.611	BB	159.365	95.91	17.384	18.071
4	16.411	BB	0.724	0.44	16.118	16.731

Analytic HPLC of **Pep$_4$**

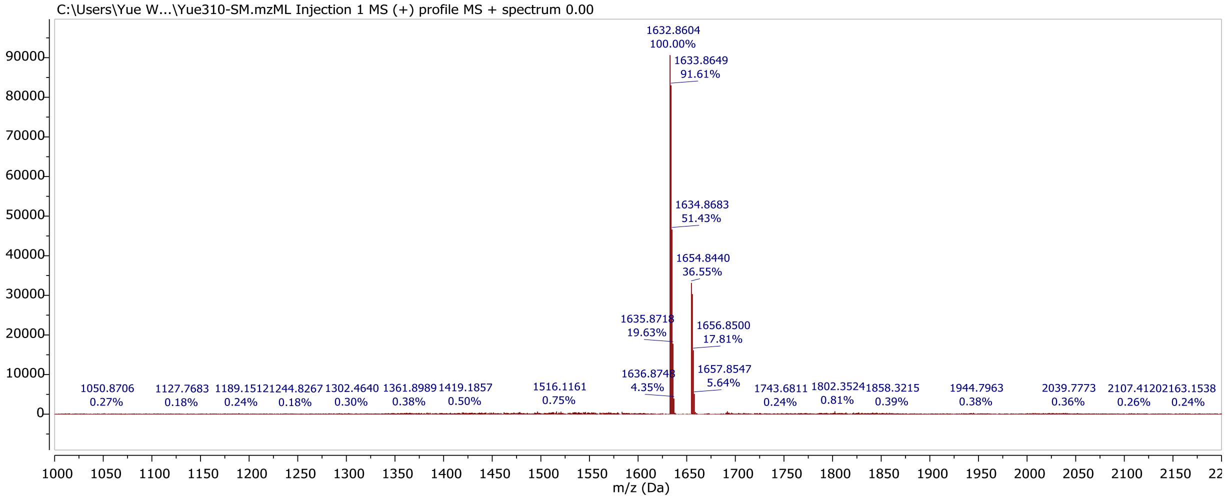

HRMS(MAIDL-TOF) of **Pep$_4$**

Pep$_5$: H$_2$N-NWQYFIVF-COOH

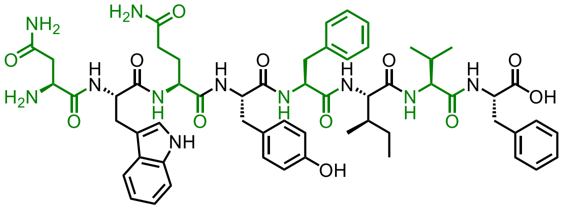

Chemical Formula: $C_{58}H_{73}N_{11}O_{12}$

Exact Mass: 1115.5440

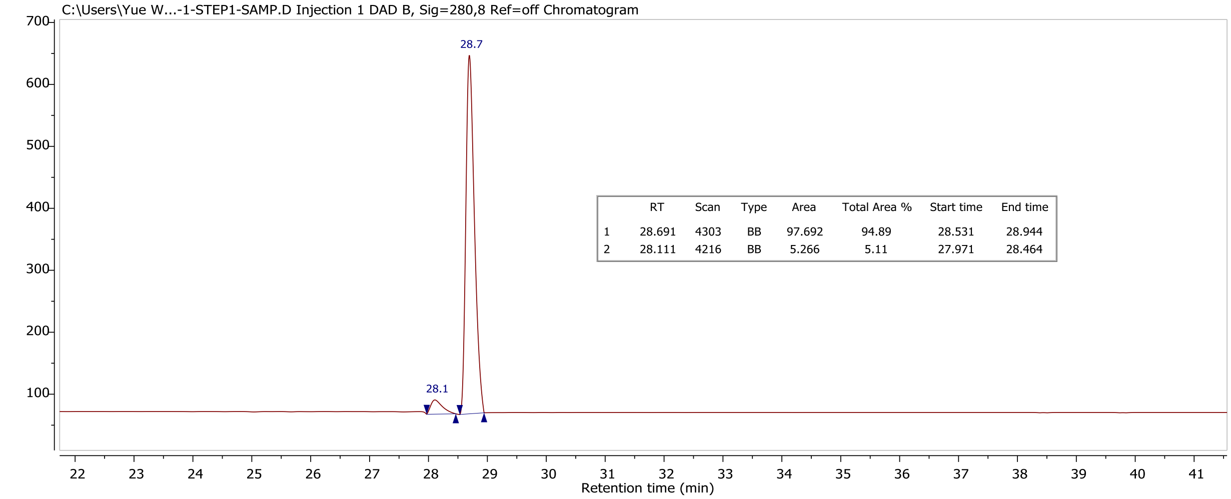

Analytic HPLC of **Pep$_5$**

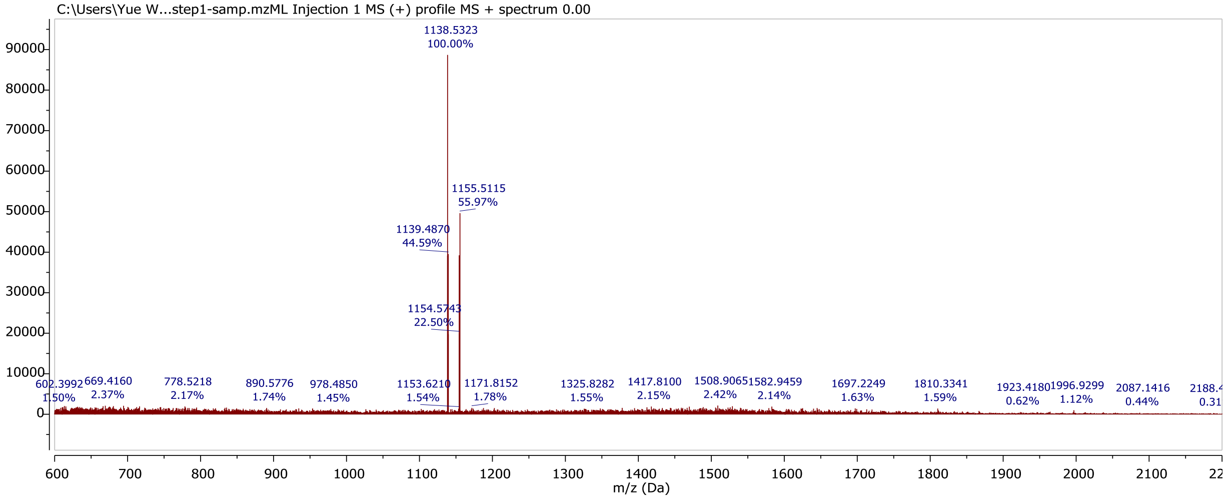

HRMS(MAIDL-TOF) of **Pep$_5$**

Pep$_6$: H$_2$N-EHDYFIVF-COOH

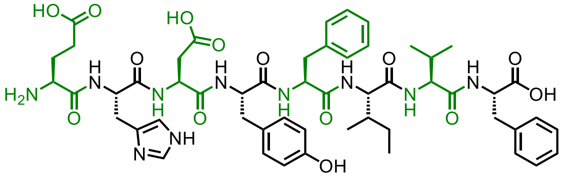

Chemical Formula: $C_{53}H_{68}N_{10}O_{14}$

Exact Mass: 1068.4916

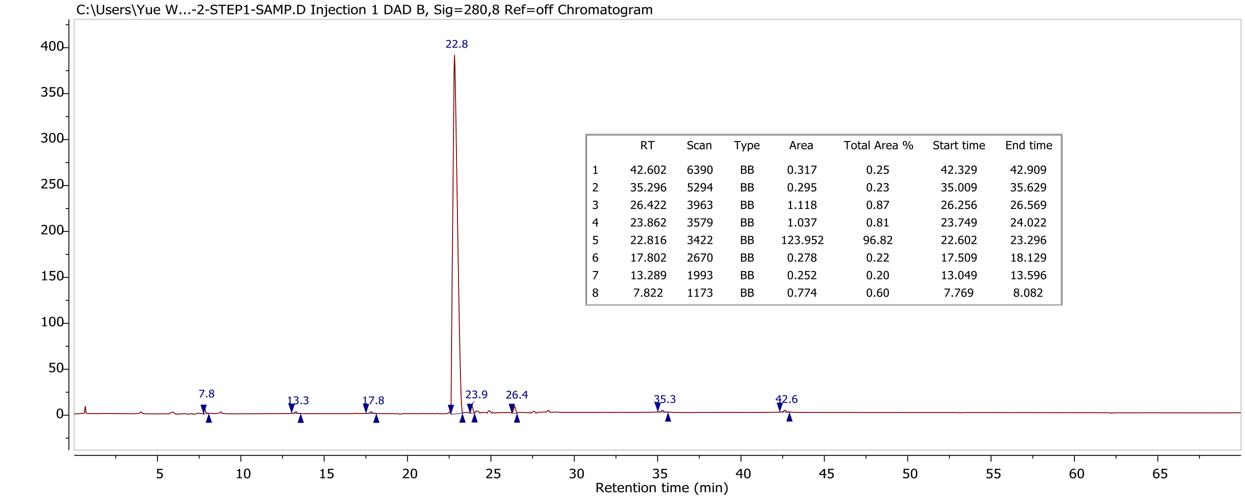

	RT	Scan	Type	Area	Total Area %	Start time	End time
1	42.602	6390	BB	0.317	0.25	42.329	42.909
2	35.296	5294	BB	0.295	0.23	35.009	35.629
3	26.422	3963	BB	1.118	0.87	26.256	26.569
4	23.862	3579	BB	1.037	0.81	23.749	24.022
5	22.816	3422	BB	123.952	96.82	22.602	23.296
6	17.802	2670	BB	0.278	0.22	17.509	18.129
7	13.289	1993	BB	0.252	0.20	13.049	13.596
8	7.822	1173	BB	0.774	0.60	7.769	8.082

Analytic HPLC of **Pep$_6$**

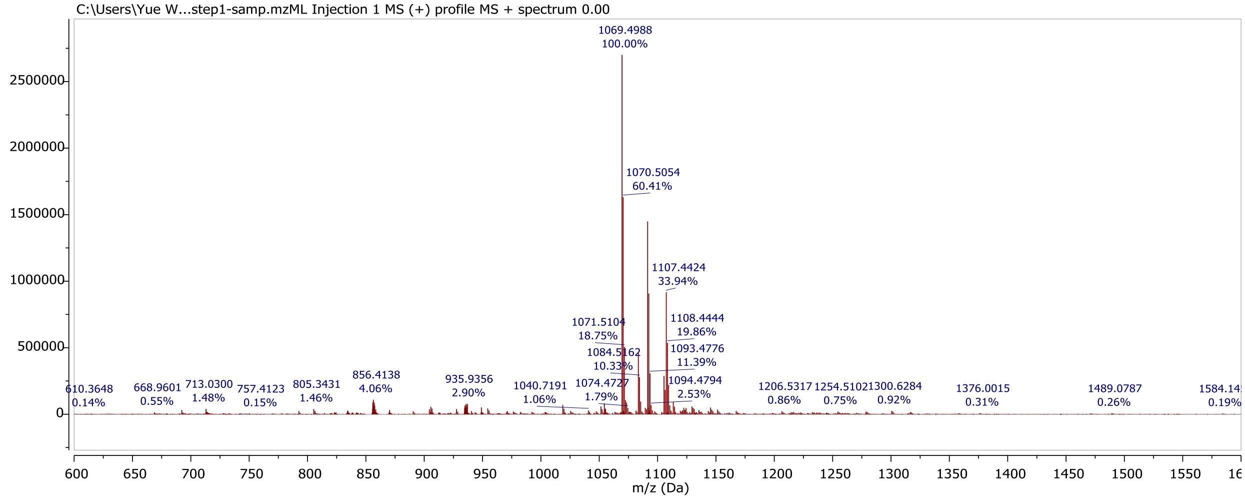

HRMS(MAIDL-TOF) of **Pep$_6$**

Pep$_7$: H_2N-Ahx-P-(pTyr)-LKTK-COOH

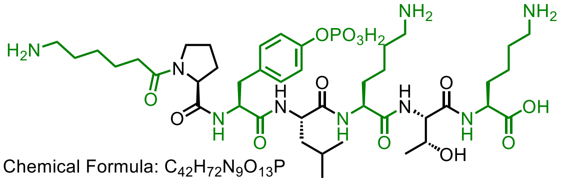

Chemical Formula: $C_{42}H_{72}N_9O_{13}P$

Exact Mass: 941.4987

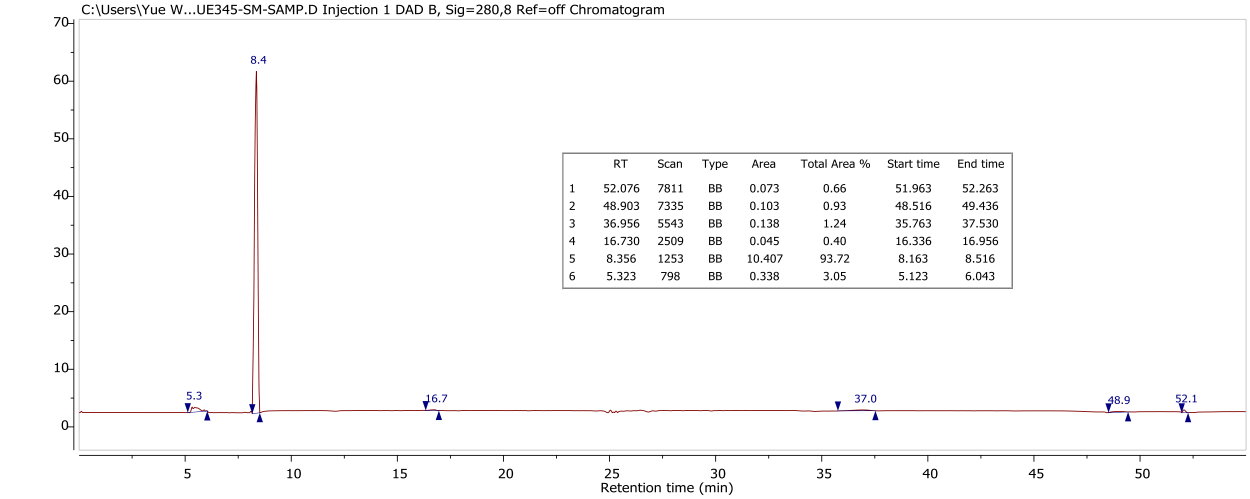

	RT	Scan	Type	Area	Total Area %	Start time	End time
1	52.076	7811	BB	0.073	0.66	51.963	52.263
2	48.903	7335	BB	0.103	0.93	48.516	49.436
3	36.956	5543	BB	0.138	1.24	35.763	37.530
4	16.730	2509	BB	0.045	0.40	16.336	16.956
5	8.356	1253	BB	10.407	93.72	8.163	8.516
6	5.323	798	BB	0.338	3.05	5.123	6.043

Analytic HPLC of **Pep$_7$**

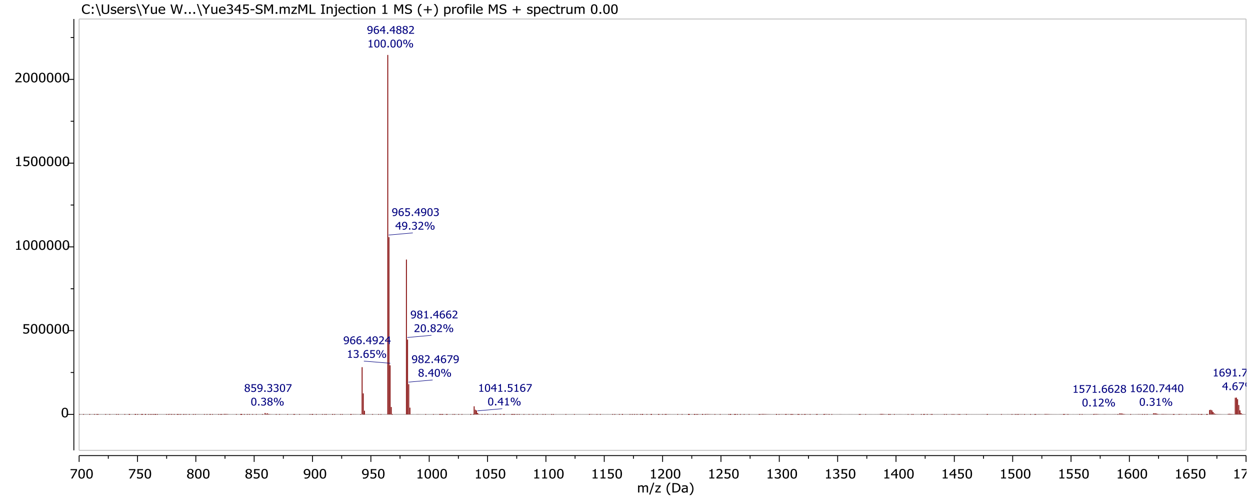

HRMS(MAIDL-TOF) of **Pep$_7$**

Pep₈: H₂N-Ahx-P-(pTyr)-LKTKRrRK-COOH

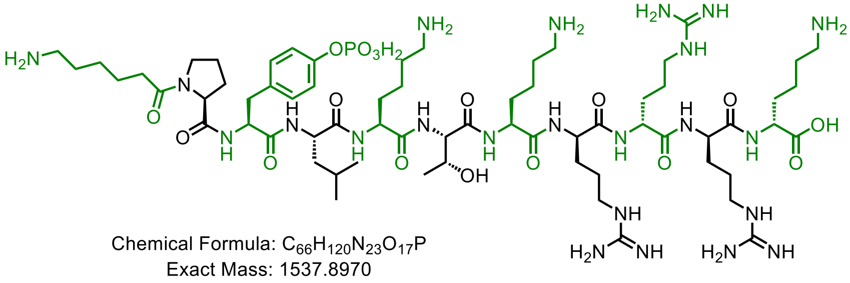

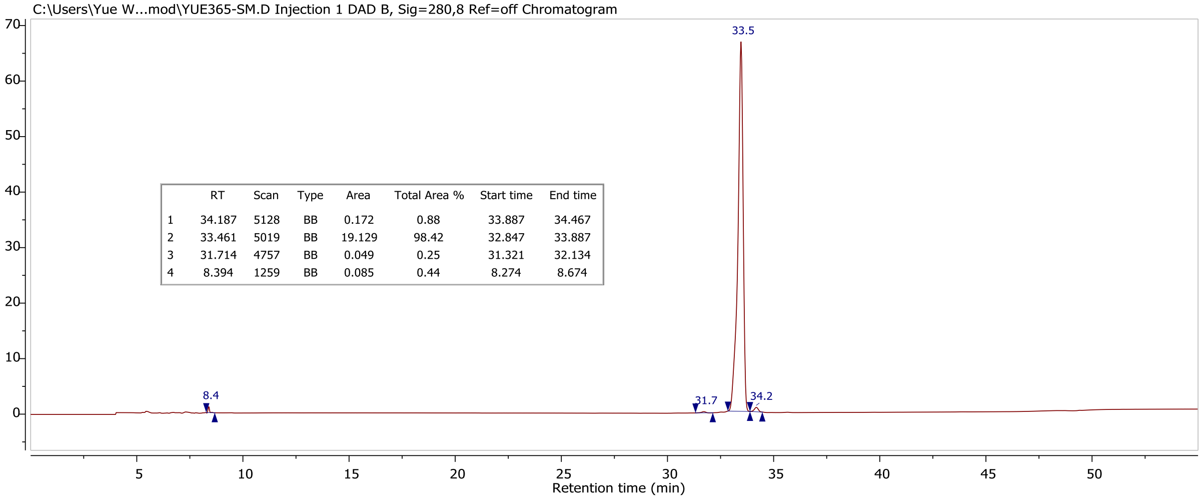

	RT	Scan	Type	Area	Total Area %	Start time	End time
1	34.187	5128	BB	0.172	0.88	33.887	34.467
2	33.461	5019	BB	19.129	98.42	32.847	33.887
3	31.714	4757	BB	0.049	0.25	31.321	32.134
4	8.394	1259	BB	0.085	0.44	8.274	8.674

Analytic HPLC of **Pep₈**

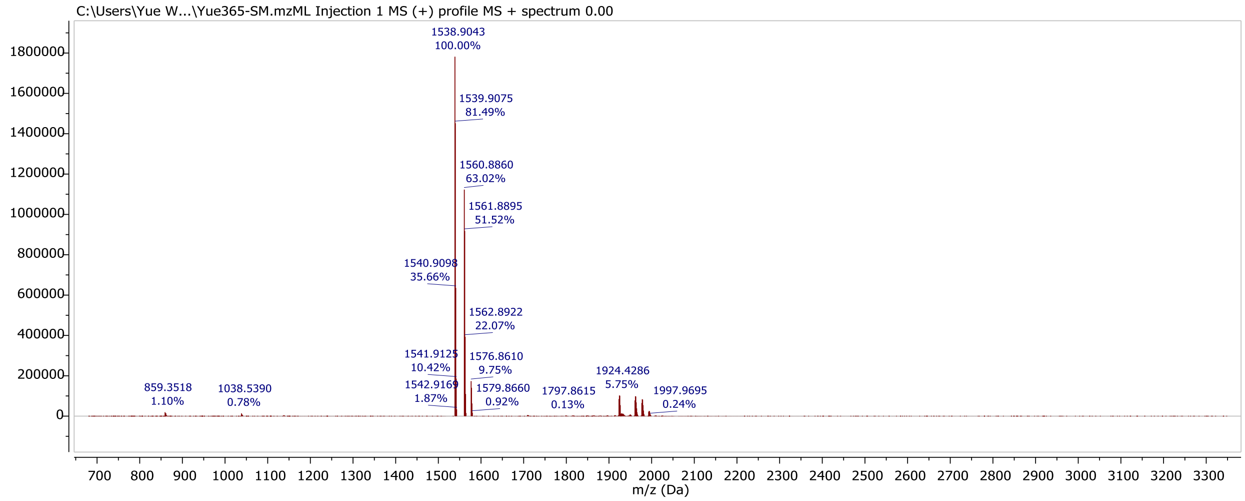

HRMS(MAIDL-TOF) of **Pep₈**

Pep$_9$: H$_2$N-Nal-Cpa-Pal-SY-Cit-LRPG-CONH$_2$

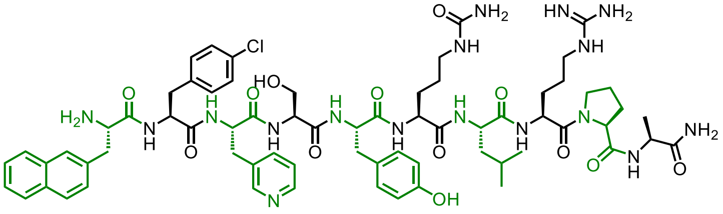

Chemical Formula: $C_{68}H_{90}ClN_{17}O_{13}$
Exact Mass: 1387.6593

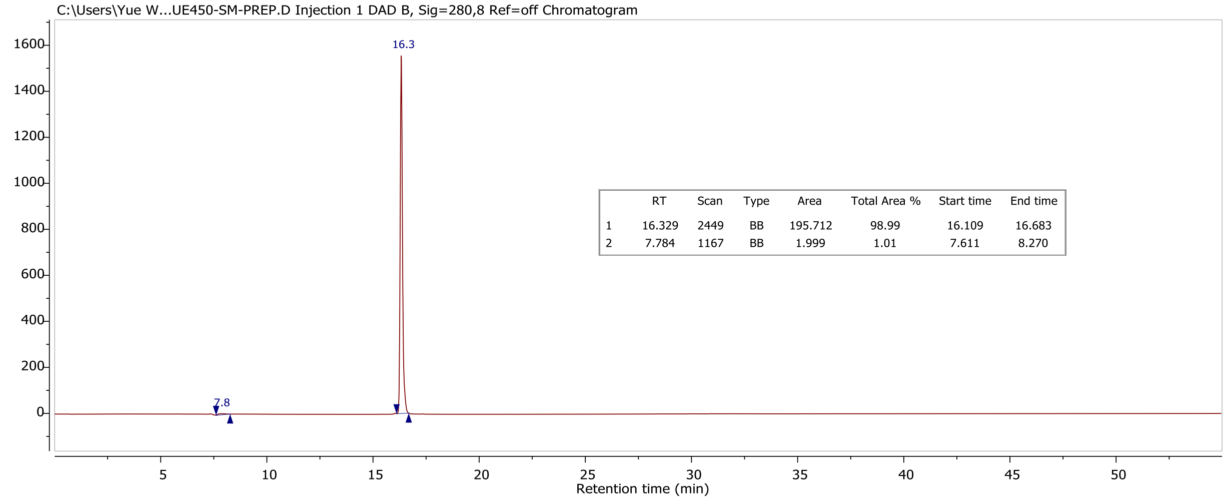

	RT	Scan	Type	Area	Total Area %	Start time	End time
1	16.329	2449	BB	195.712	98.99	16.109	16.683
2	7.784	1167	BB	1.999	1.01	7.611	8.270

Analytic HPLC of **Pep$_9$**

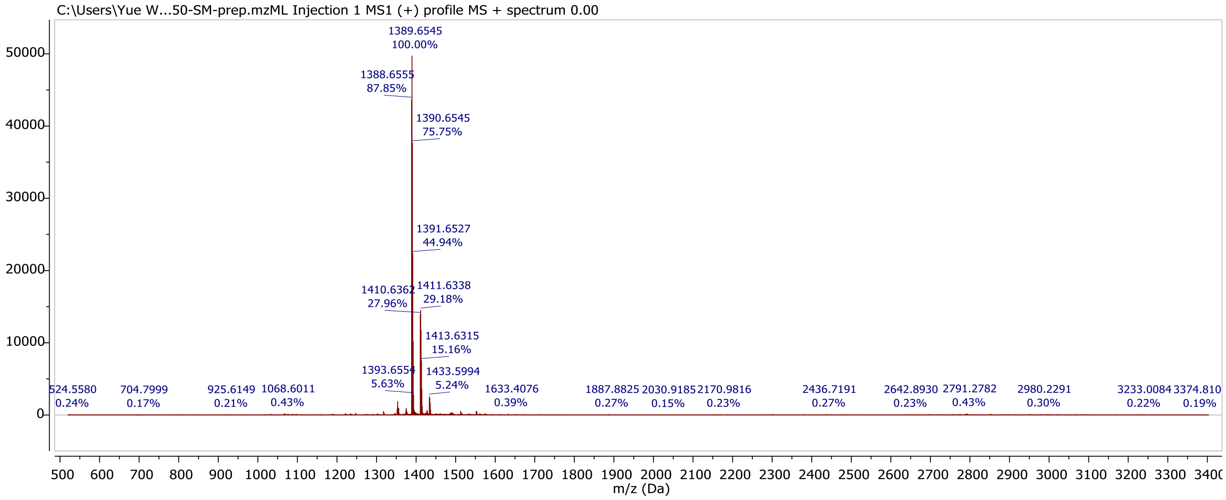

HRMS(MAIDL-TOF) of **Pep$_9$**

Pep$_{10}$: H$_2$N-DRVYIHPF-CONH$_2$

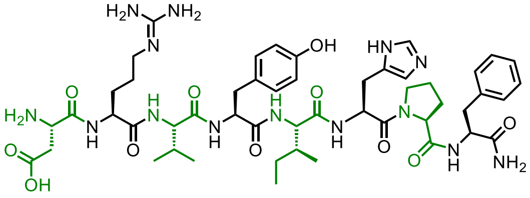

Chemical Formula: $C_{50}H_{72}N_{14}O_{11}$
Exact Mass: 1044.5505

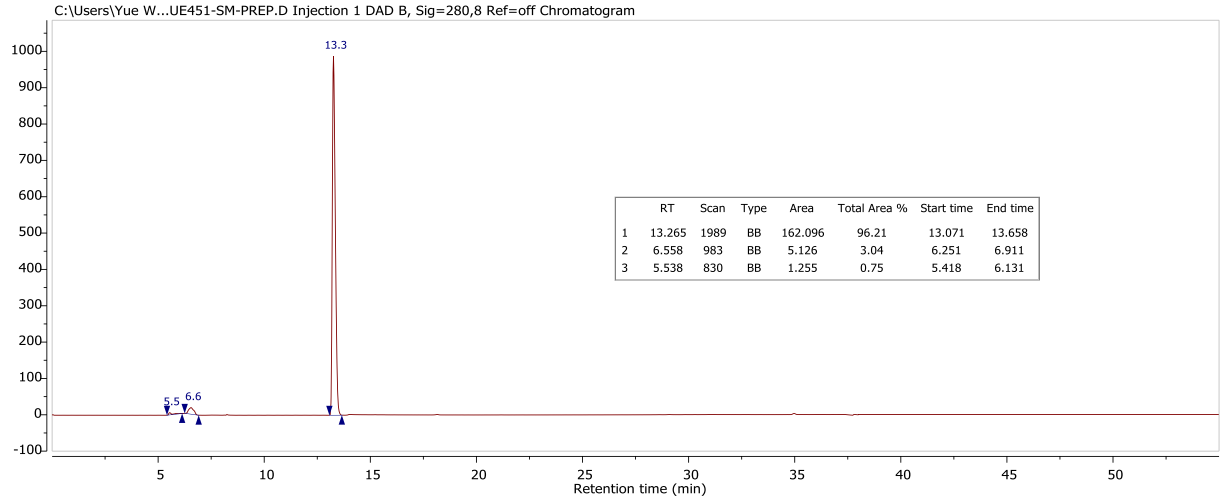

	RT	Scan	Type	Area	Total Area %	Start time	End time
1	13.265	1989	BB	162.096	96.21	13.071	13.658
2	6.558	983	BB	5.126	3.04	6.251	6.911
3	5.538	830	BB	1.255	0.75	5.418	6.131

Analytic HPLC of **Pep$_{10}$**

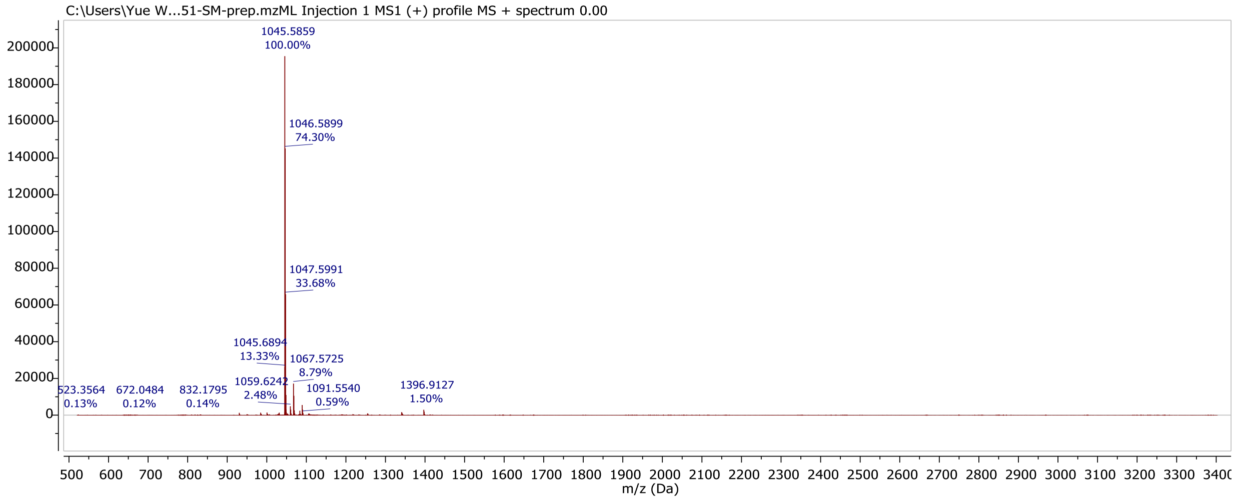

HRMS(MAIDL-TOF) of **Pep$_{10}$**

Pep$_{11}$: H$_2$N-GHL-CONH$_2$

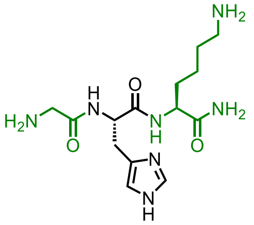

Chemical Formula: $C_{14}H_{25}N_7O_3$
Exact Mass: 339.2019

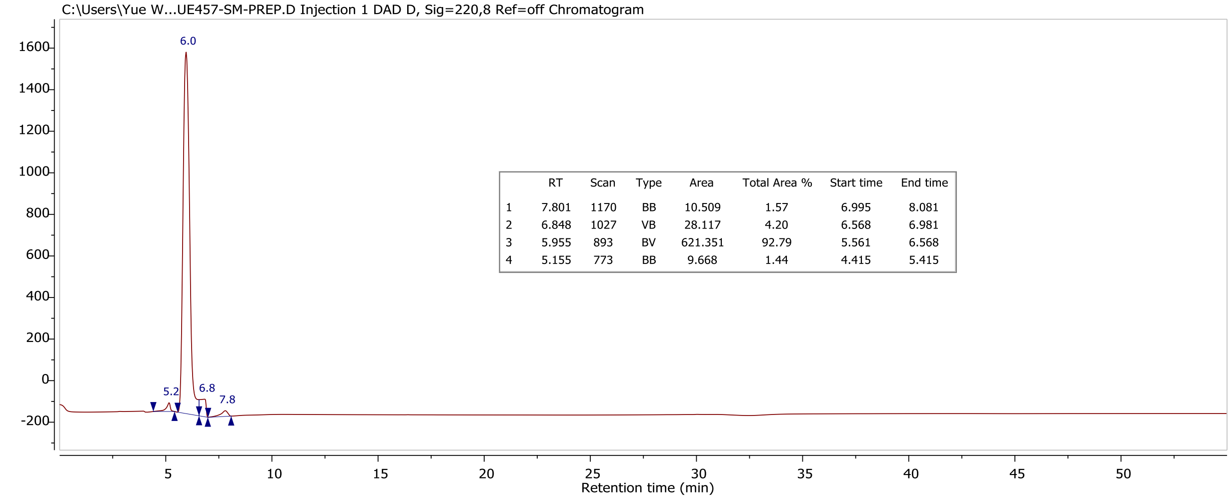

	RT	Scan	Type	Area	Total Area %	Start time	End time
1	7.801	1170	BB	10.509	1.57	6.995	8.081
2	6.848	1027	VB	28.117	4.20	6.568	6.981
3	5.955	893	BV	621.351	92.79	5.561	6.568
4	5.155	773	BB	9.668	1.44	4.415	5.415

Analytic HPLC of **Pep$_{11}$**

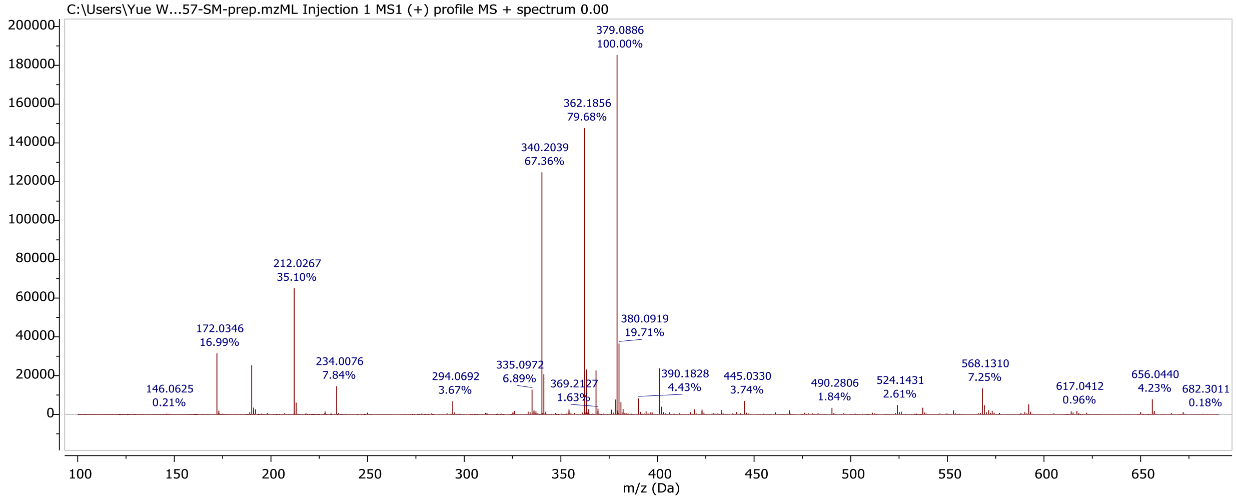

HRMS(MAIDL-TOF) of **Pep$_{11}$**

DP$_1$-Pep$_1$: DP$_1$-NH-YFMVF-CONH$_2$

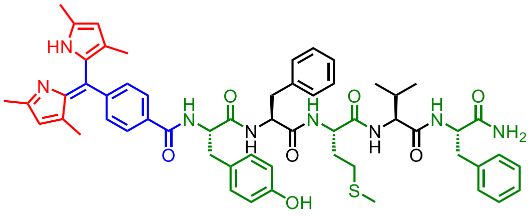

Chemical Formula: $C_{57}H_{66}N_8O_7S$
Exact Mass: 1006.4775

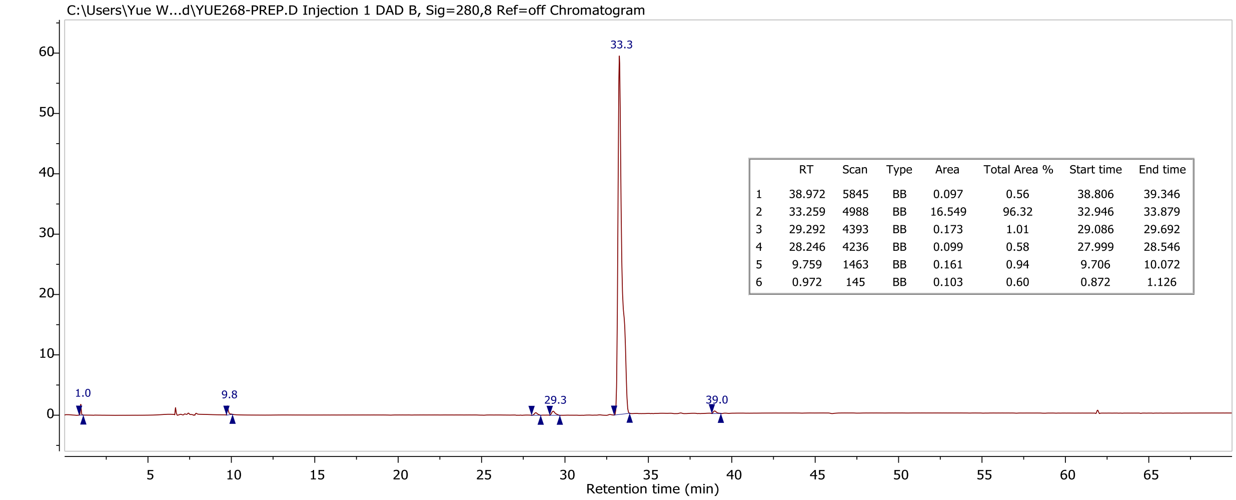

	RT	Scan	Type	Area	Total Area %	Start time	End time
1	38.972	5845	BB	0.097	0.56	38.806	39.346
2	33.259	4988	BB	16.549	96.32	32.946	33.879
3	29.292	4393	BB	0.173	1.01	29.086	29.692
4	28.246	4236	BB	0.099	0.58	27.999	28.546
5	9.759	1463	BB	0.161	0.94	9.706	10.072
6	0.972	145	BB	0.103	0.60	0.872	1.126

Analytic HPLC of **DP$_1$-Pep$_1$**

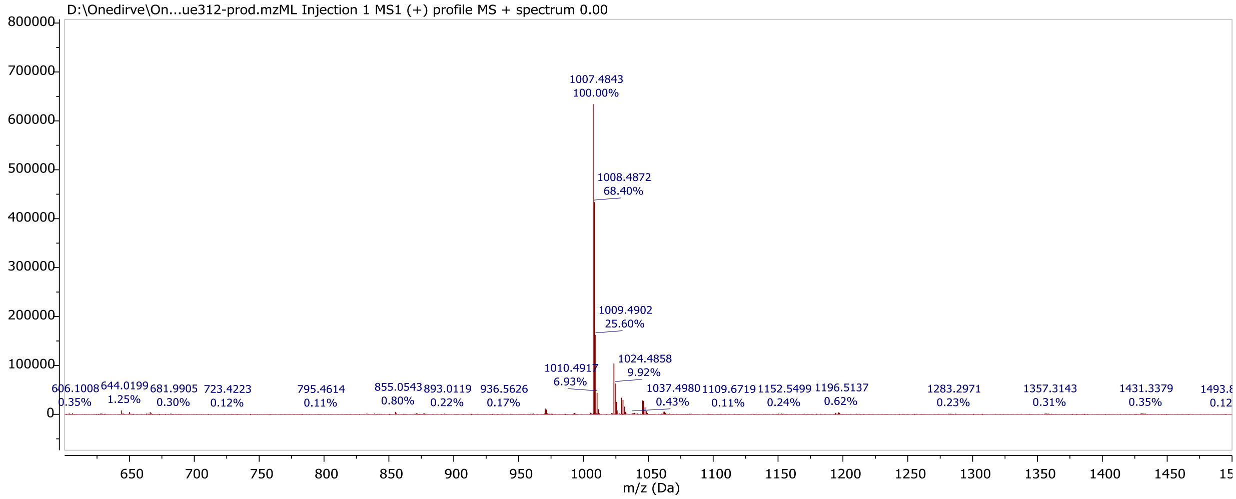

HRMS(MAIDL-TOF) of **DP$_1$-Pep$_1$**

DP_2-Pep_1: DP_2-NH-YFMVF-$CONH_2$

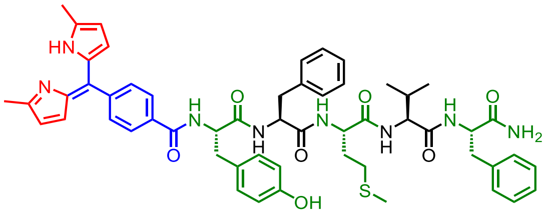

Chemical Formula: $C_{55}H_{62}N_8O_7S$
Exact Mass: 978.4462

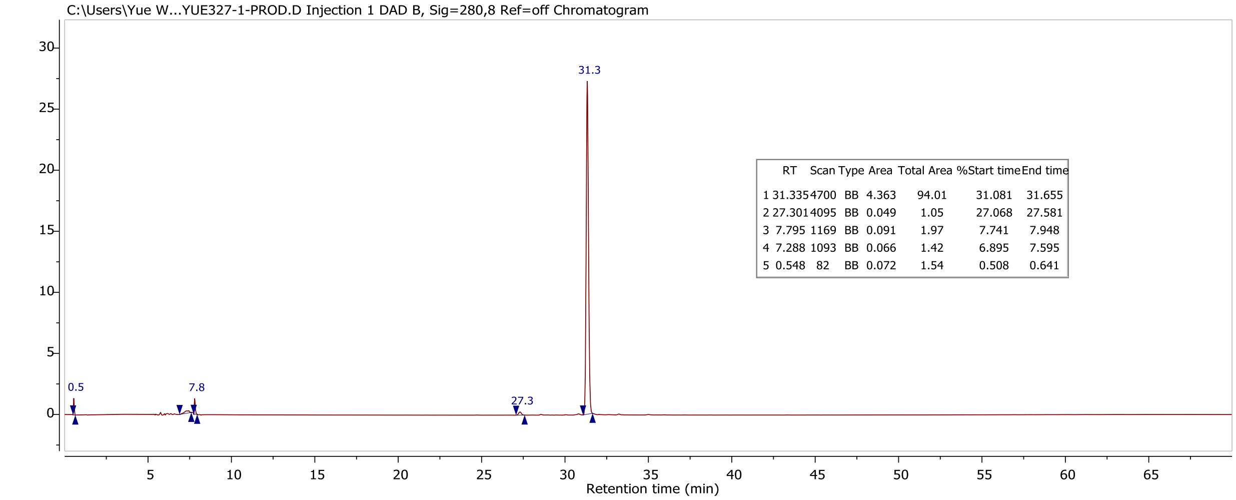

Analytic HPLC of **DP_2-Pep_1**

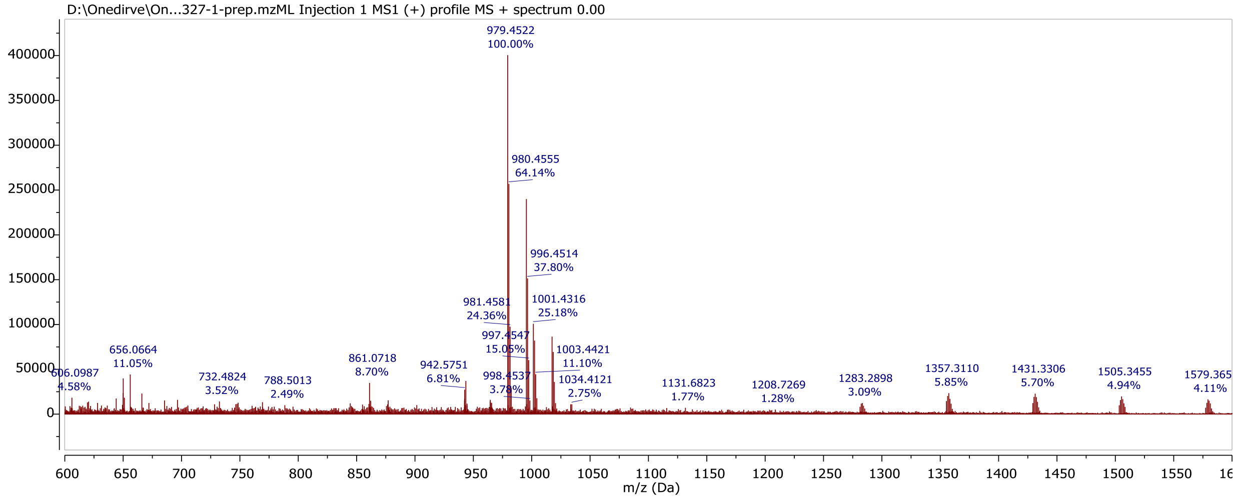

HRMS(MAIDL-TOF) of **DP_2-Pep_1**

DP_3-Pep_1: DP_3-NH-YFMVF-$CONH_2$

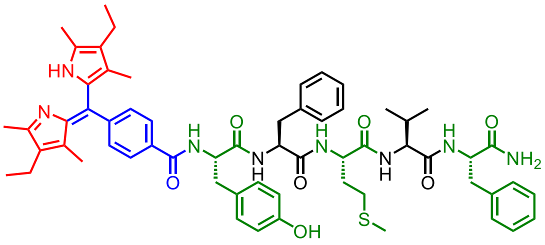

Chemical Formula: $C_{61}H_{74}N_8O_7S$
Exact Mass: 1062.5401

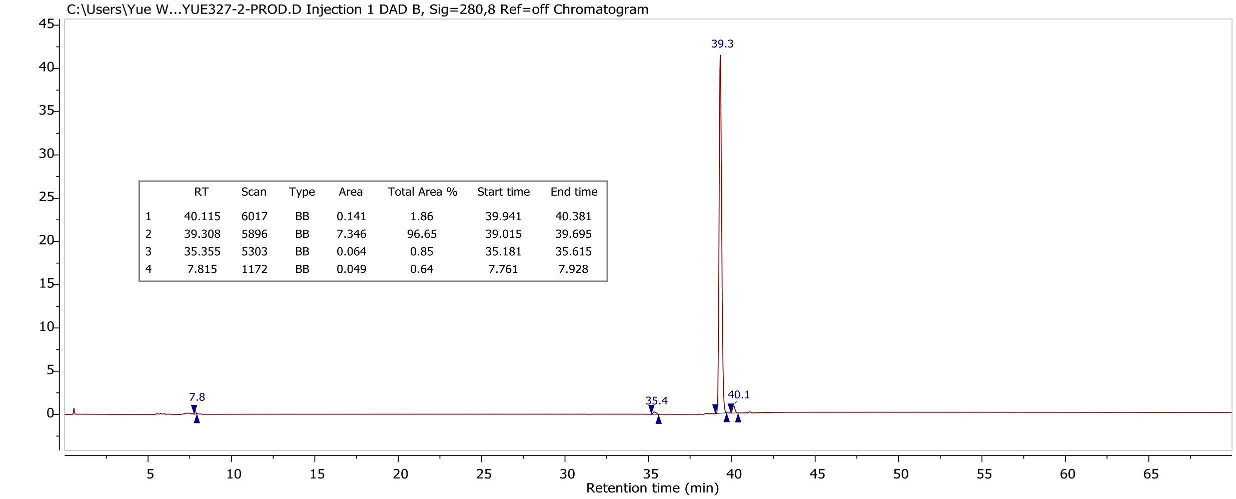

	RT	Scan	Type	Area	Total Area %	Start time	End time
1	40.115	6017	BB	0.141	1.86	39.941	40.381
2	39.308	5896	BB	7.346	96.65	39.015	39.695
3	35.355	5303	BB	0.064	0.85	35.181	35.615
4	7.815	1172	BB	0.049	0.64	7.761	7.928

Analytic HPLC of **DP_3-Pep_1**

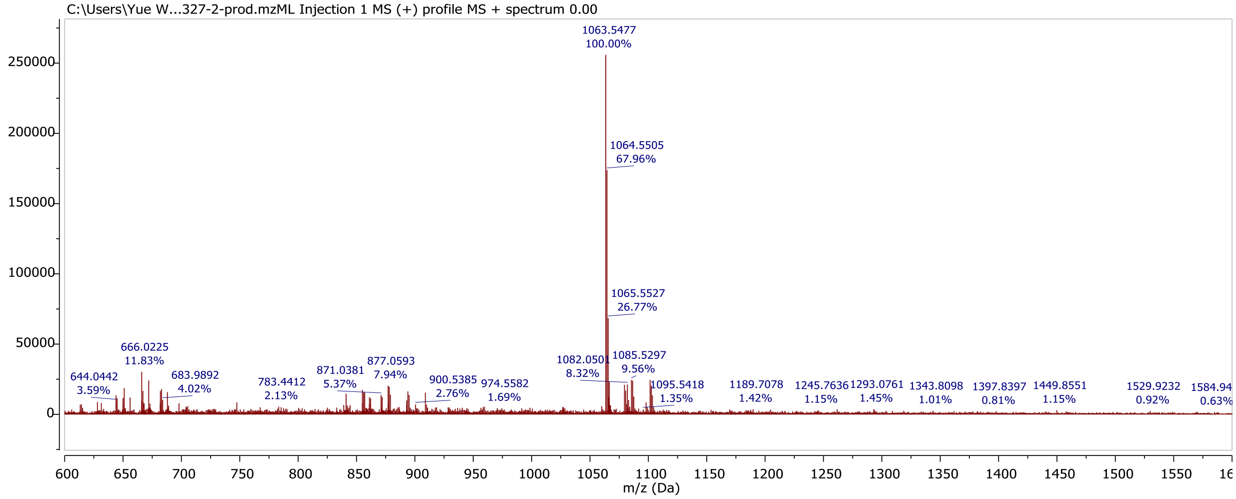

HRMS(MAIDL-TOF) of **DP_3-Pep_1**

DP$_4$-Pep$_1$: DP$_4$-NH-YFMVF-CONH$_2$

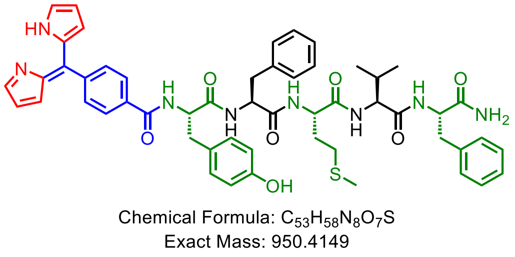

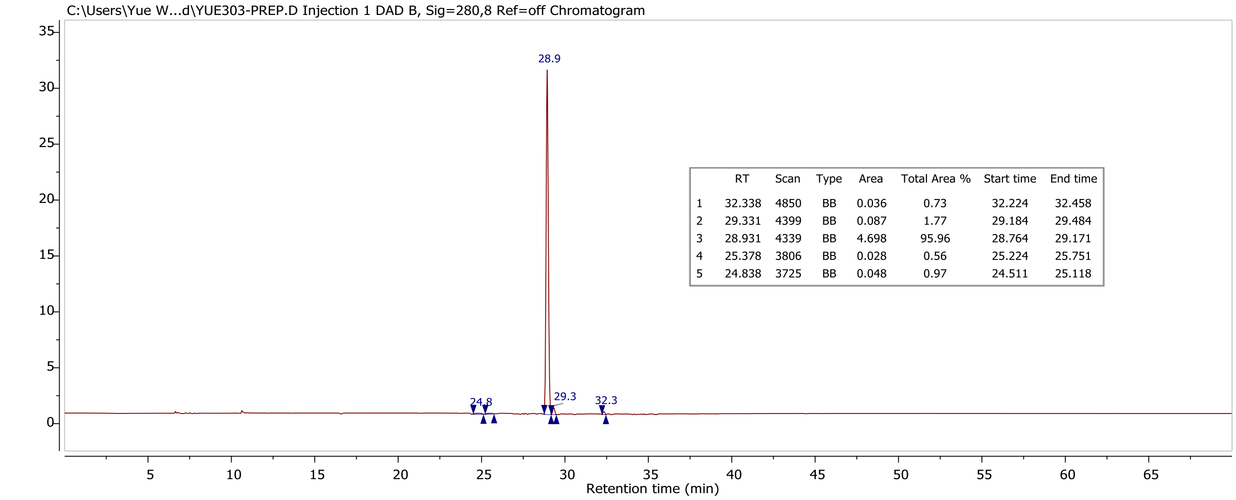

	RT	Scan	Type	Area	Total Area %	Start time	End time
1	32.338	4850	BB	0.036	0.73	32.224	32.458
2	29.331	4399	BB	0.087	1.77	29.184	29.484
3	28.931	4339	BB	4.698	95.96	28.764	29.171
4	25.378	3806	BB	0.028	0.56	25.224	25.751
5	24.838	3725	BB	0.048	0.97	24.511	25.118

Analytic HPLC of **DP$_4$-Pep$_1$**

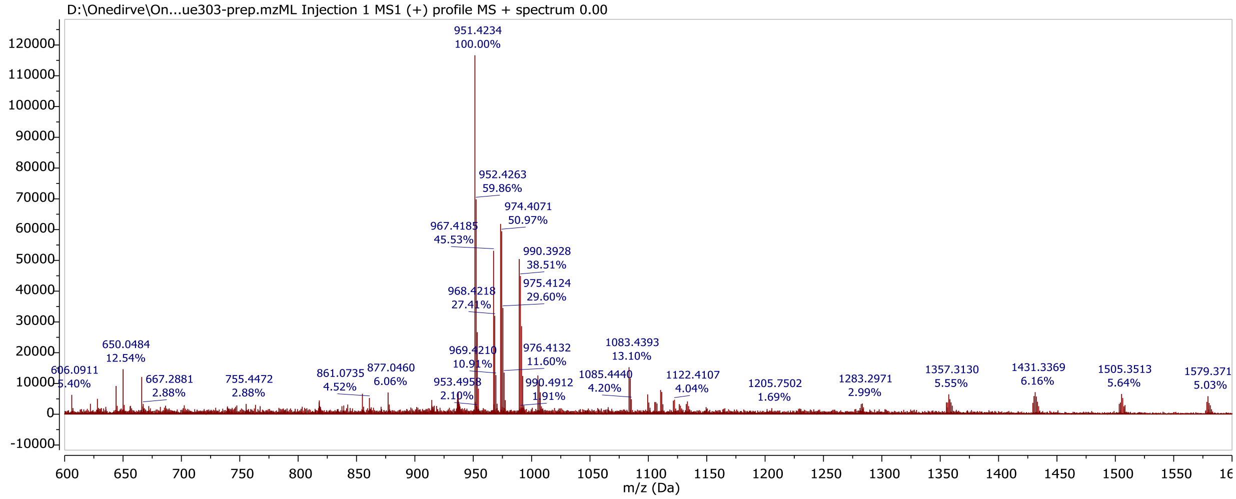

HRMS(MAIDL-TOF) of **DP$_4$-Pep$_1$**

DP$_5$-Pep$_1$: DP$_5$-NH-YFMVF-CONH$_2$

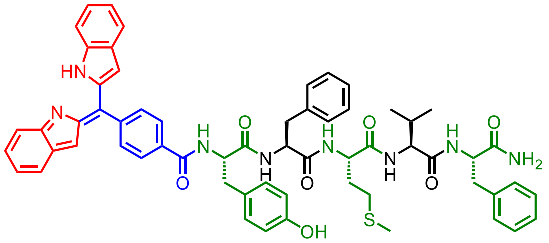

Chemical Formula: $C_{61}H_{62}N_8O_7S$
Exact Mass: 1050.4462

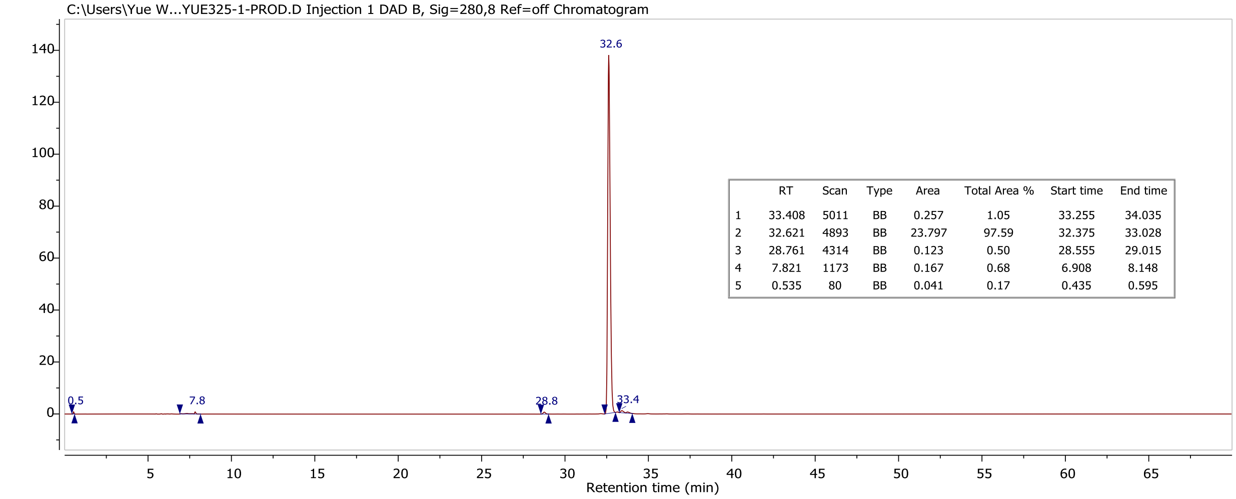

	RT	Scan	Type	Area	Total Area %	Start time	End time
1	33.408	5011	BB	0.257	1.05	33.255	34.035
2	32.621	4893	BB	23.797	97.59	32.375	33.028
3	28.761	4314	BB	0.123	0.50	28.555	29.015
4	7.821	1173	BB	0.167	0.68	6.908	8.148
5	0.535	80	BB	0.041	0.17	0.435	0.595

Analytic HPLC of **DP$_5$-Pep$_1$**

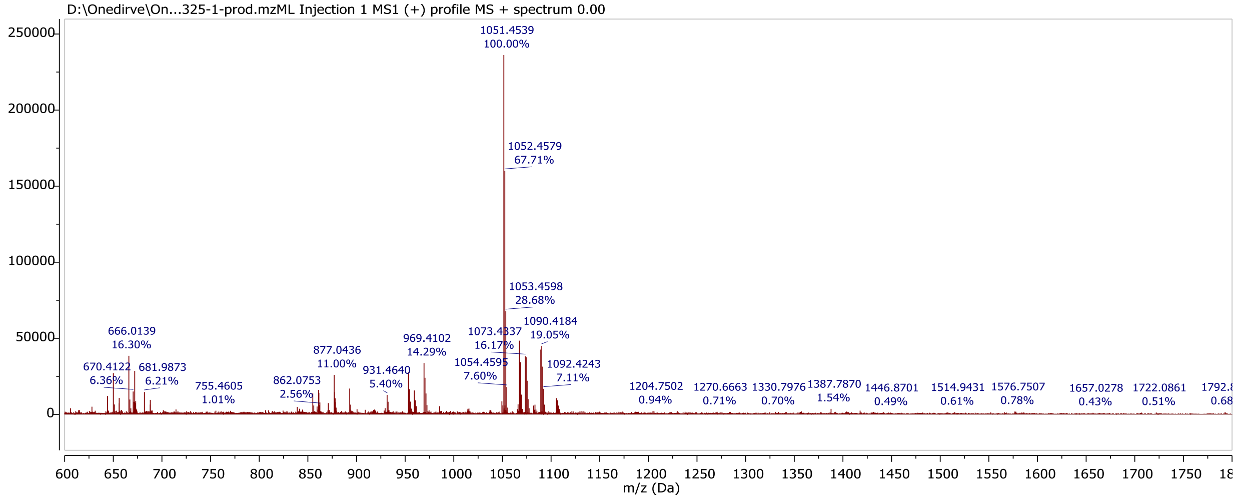

HRMS(MAIDL-TOF) of **DP$_5$-Pep$_1$**

DP_6-Pep_1: DP_6-NH-YFMVF-$CONH_2$

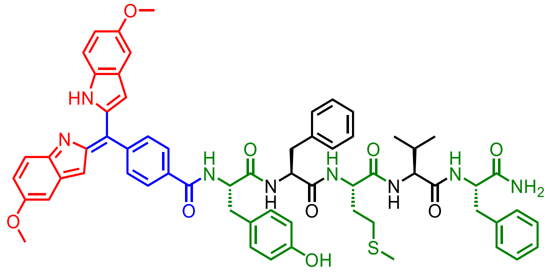

Chemical Formula: $C_{63}H_{66}N_8O_9S$
Exact Mass: 1110.4673

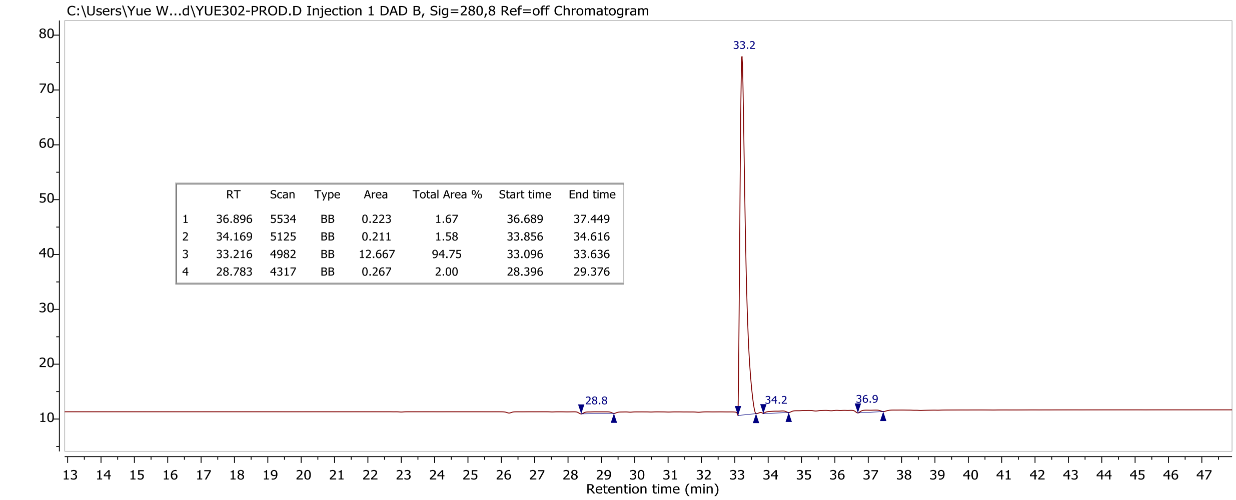

	RT	Scan	Type	Area	Total Area %	Start time	End time
1	36.896	5534	BB	0.223	1.67	36.689	37.449
2	34.169	5125	BB	0.211	1.58	33.856	34.616
3	33.216	4982	BB	12.667	94.75	33.096	33.636
4	28.783	4317	BB	0.267	2.00	28.396	29.376

Analytic HPLC of **DP_6-Pep_1**

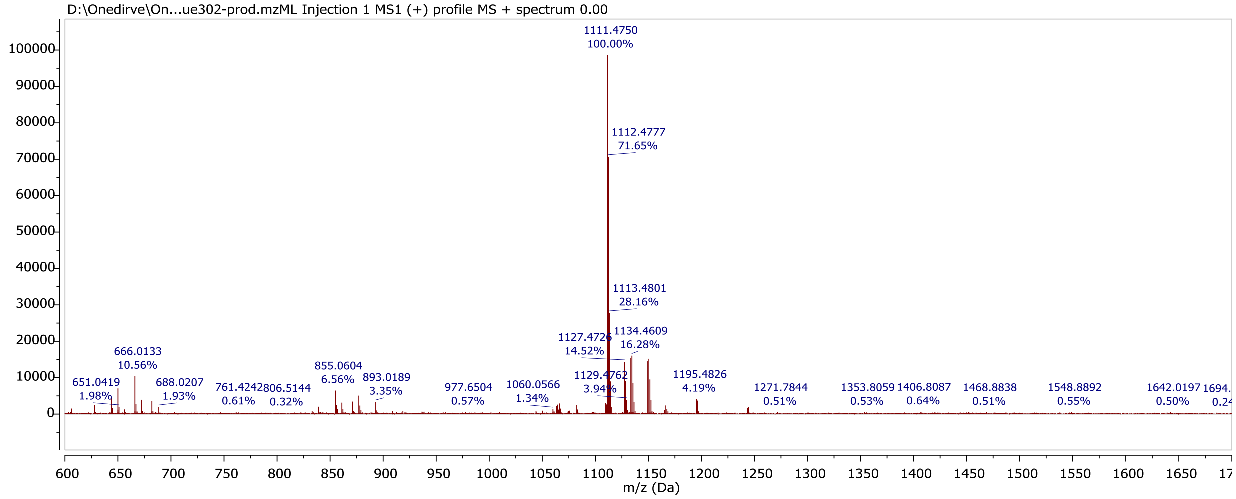

HRMS(MAIDL-TOF) of **DP_6-Pep_1**

DP_9-Pep_1: DP_9-NH-YFMVF-$CONH_2$

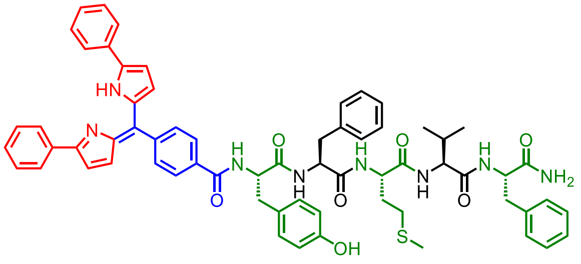

Chemical Formula: $C_{65}H_{66}N_8O_7S$
Exact Mass: 1102.4775

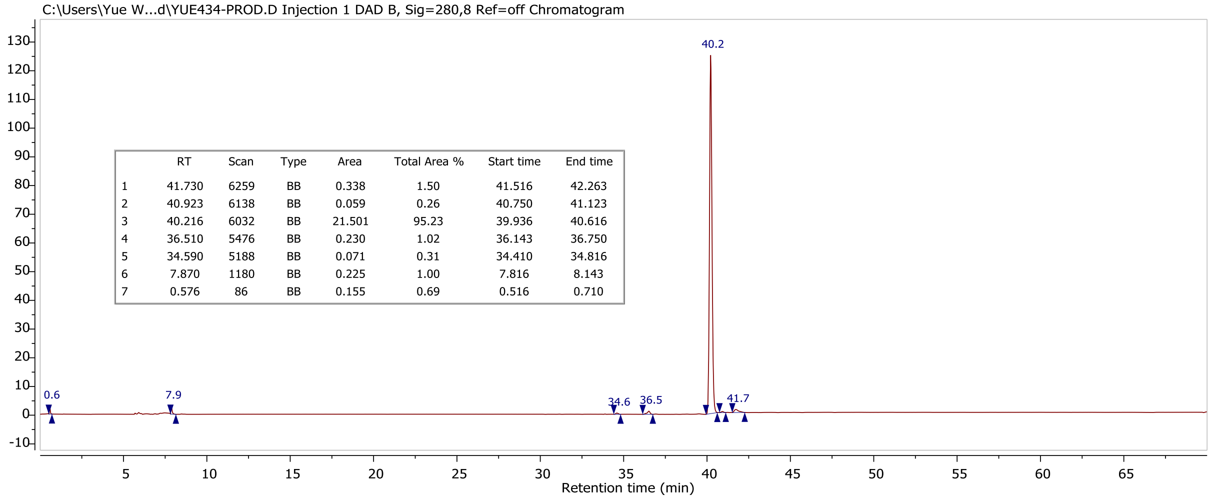

	RT	Scan	Type	Area	Total Area %	Start time	End time
1	41.730	6259	BB	0.338	1.50	41.516	42.263
2	40.923	6138	BB	0.059	0.26	40.750	41.123
3	40.216	6032	BB	21.501	95.23	39.936	40.616
4	36.510	5476	BB	0.230	1.02	36.143	36.750
5	34.590	5188	BB	0.071	0.31	34.410	34.816
6	7.870	1180	BB	0.225	1.00	7.816	8.143
7	0.576	86	BB	0.155	0.69	0.516	0.710

Analytic HPLC of **DP_9-Pep_1**

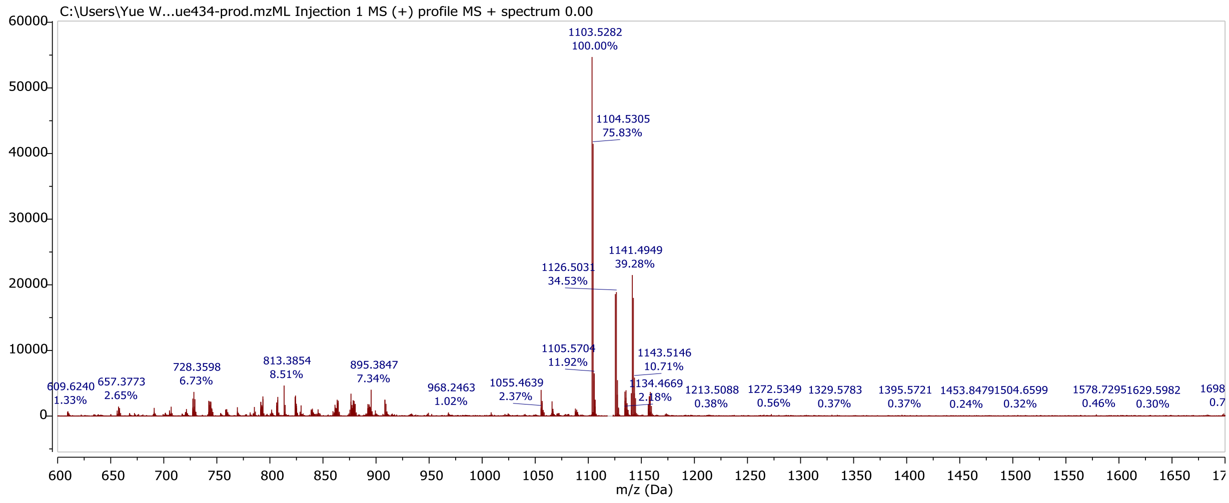

HRMS(MAIDL-TOF) of **DP_9-Pep_1**

DP_{10}-Pep_1: DP_{10}-NH-YFMVF-$CONH_2$

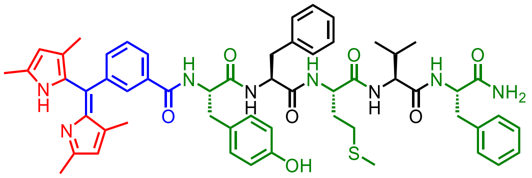

Chemical Formula: $C_{57}H_{66}N_8O_7S$
Exact Mass: 1006.4775

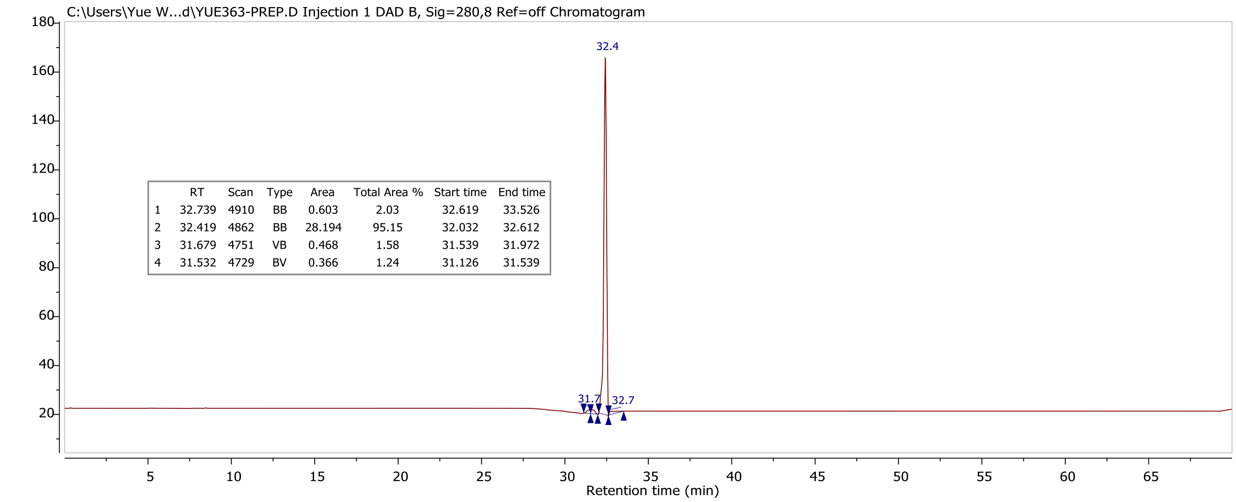

	RT	Scan	Type	Area	Total Area %	Start time	End time
1	32.739	4910	BB	0.603	2.03	32.619	33.526
2	32.419	4862	BB	28.194	95.15	32.032	32.612
3	31.679	4751	VB	0.468	1.58	31.539	31.972
4	31.532	4729	BV	0.366	1.24	31.126	31.539

Analytic HPLC of **DP_{10}-Pep_1**

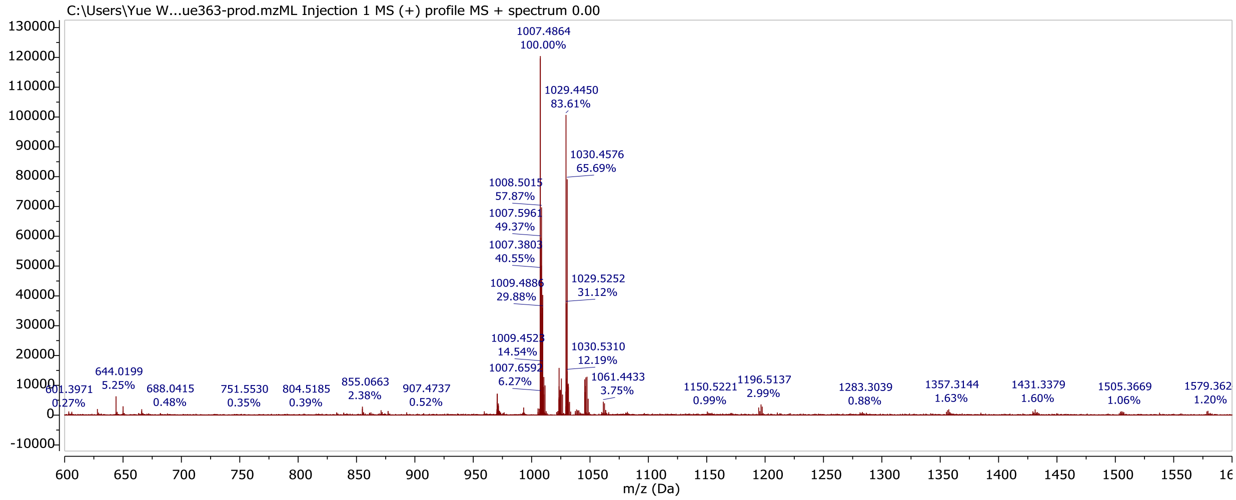

HRMS(MAIDL-TOF) of **DP_{10}-Pep_1**

DP_{12}-Pep_1: DP_{12}-NH-YFMVF-$CONH_2$

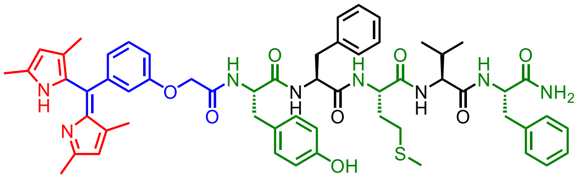

Chemical Formula: $C_{58}H_{68}N_8O_8S$
Exact Mass: 1036.4881

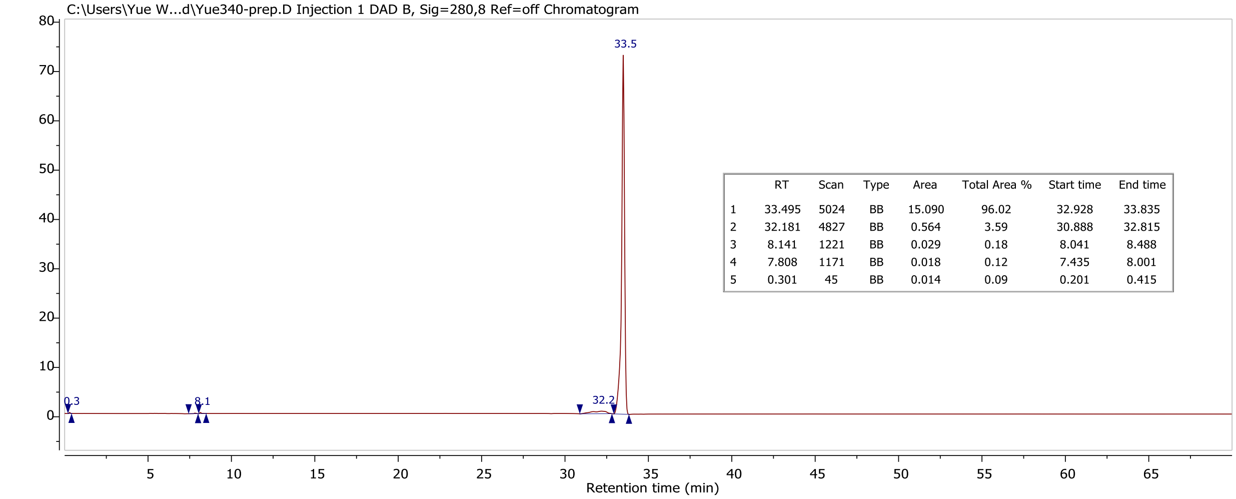

Analytic HPLC of **DP_{12}-Pep_1**

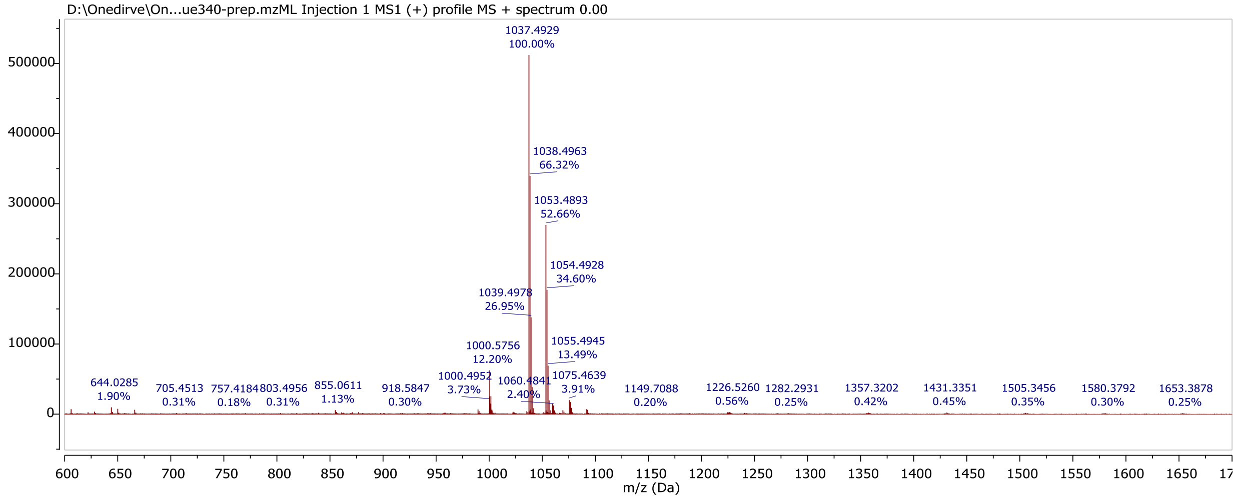

HRMS(MAIDL-TOF) of **DP_{12}-Pep_1**

DP$_{13}$-Pep$_1$: DP$_{13}$-NH-YFMVF-CONH$_2$

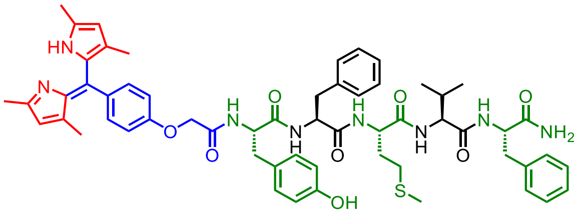

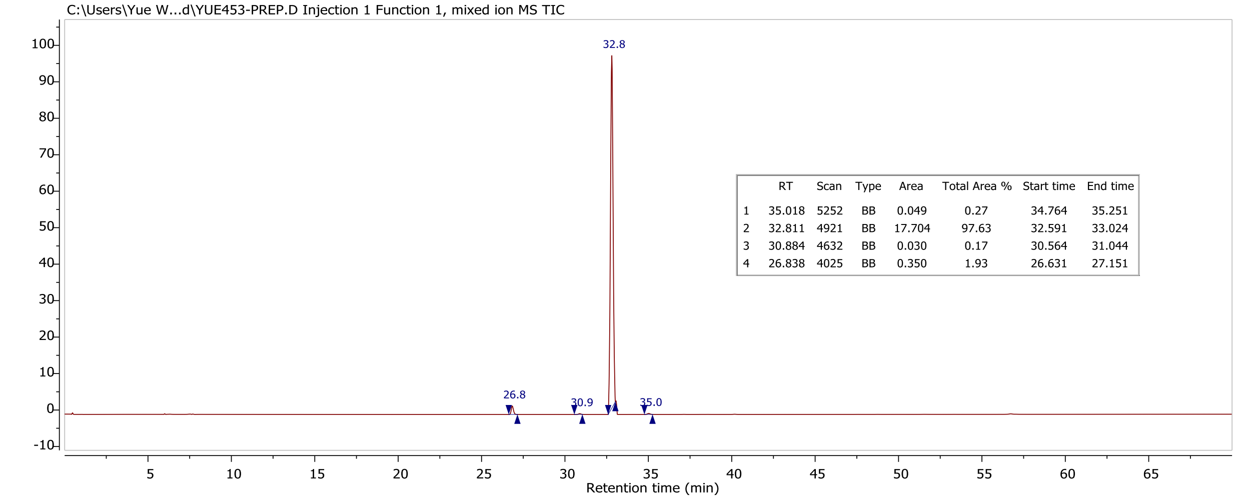

	RT	Scan	Type	Area	Total Area %	Start time	End time
1	35.018	5252	BB	0.049	0.27	34.764	35.251
2	32.811	4921	BB	17.704	97.63	32.591	33.024
3	30.884	4632	BB	0.030	0.17	30.564	31.044
4	26.838	4025	BB	0.350	1.93	26.631	27.151

Analytic HPLC of **DP$_{13}$-Pep$_1$**

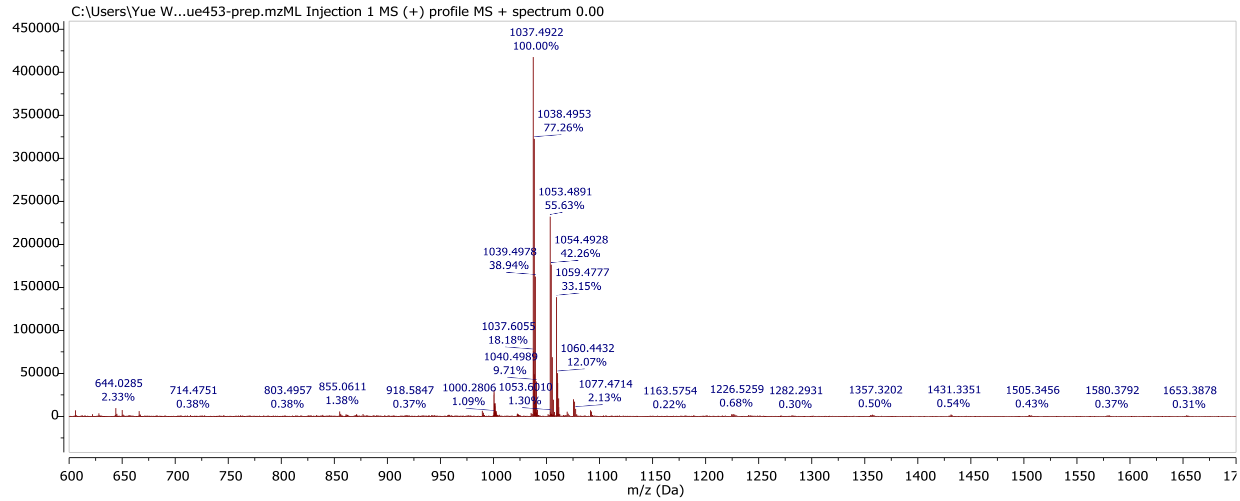

HRMS(MAIDL-TOF) of **DP$_{13}$-Pep$_1$**

DP_{14}-Pep_1: DP_{14}-NH-YFMVF-$CONH_2$

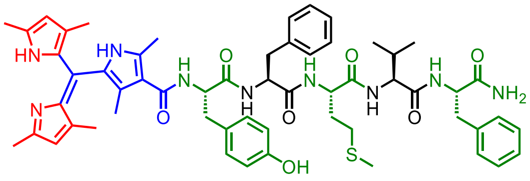

Chemical Formula: $C_{57}H_{69}N_9O_7S$
Exact Mass: 1023.5041

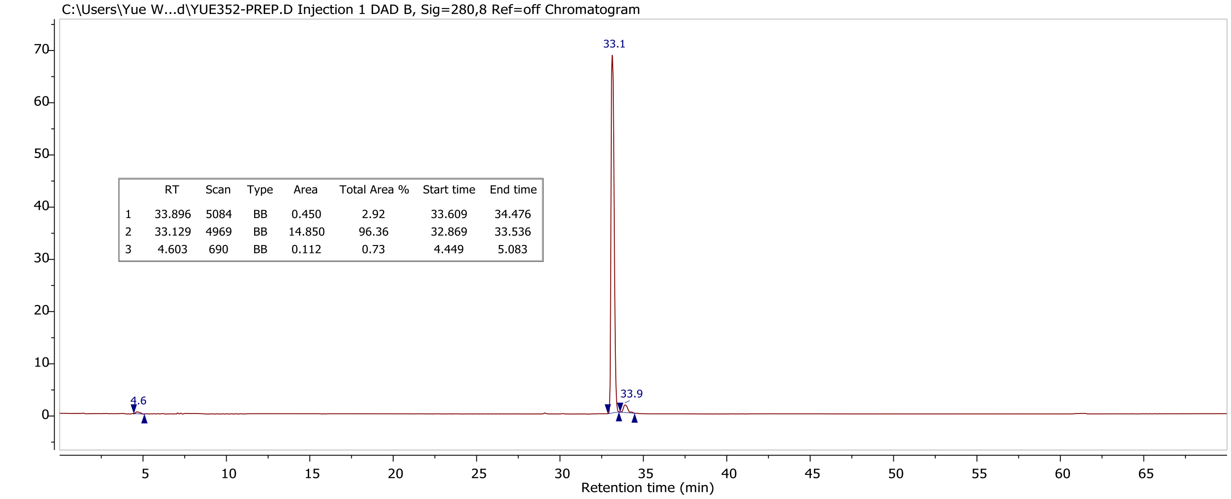

Analytic HPLC of **DP_{14}-Pep_1**

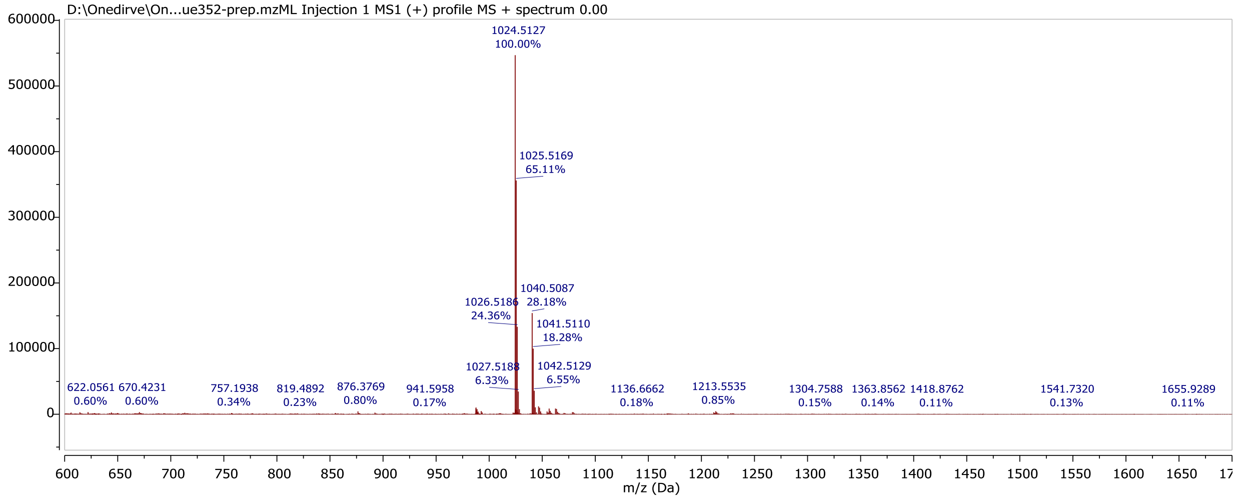

HRMS(MAIDL-TOF) of **DP_{14}-Pep_1**

DP_{15}-Pep_1: DP_{15}-NH-YFMVF-$CONH_2$

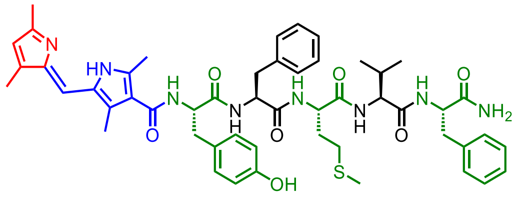

Chemical Formula: $C_{51}H_{62}N_8O_7S$
Exact Mass: 930.4462

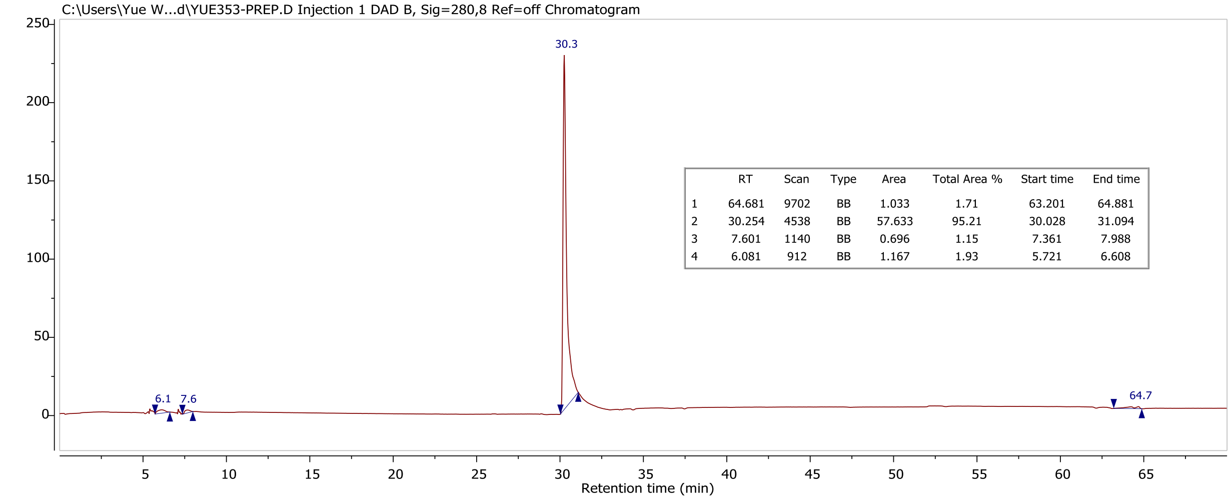

	RT	Scan	Type	Area	Total Area %	Start time	End time
1	64.681	9702	BB	1.033	1.71	63.201	64.881
2	30.254	4538	BB	57.633	95.21	30.028	31.094
3	7.601	1140	BB	0.696	1.15	7.361	7.988
4	6.081	912	BB	1.167	1.93	5.721	6.608

Analytic HPLC of **DP_{15}-Pep_1**

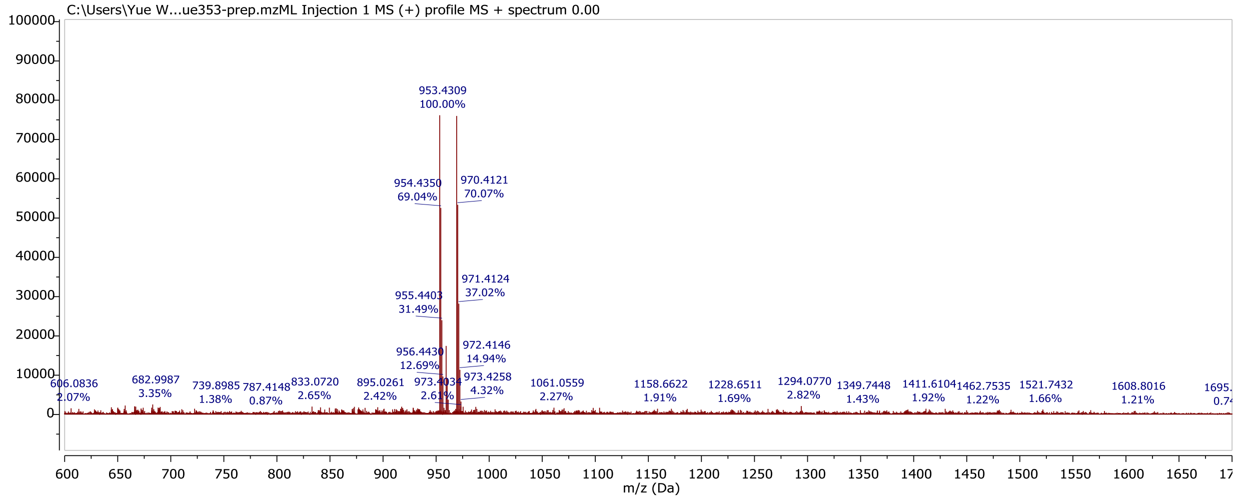

HRMS(MAIDL-TOF) of **DP_{15}-Pep_1**

DP_{16}-Pep_1: DP_{16}-NH-YFMVF-CONH$_2$

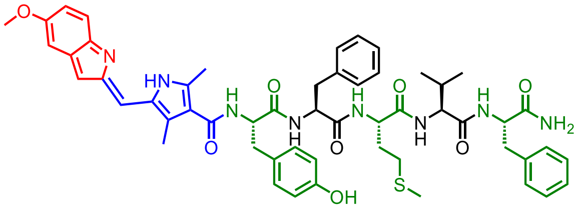

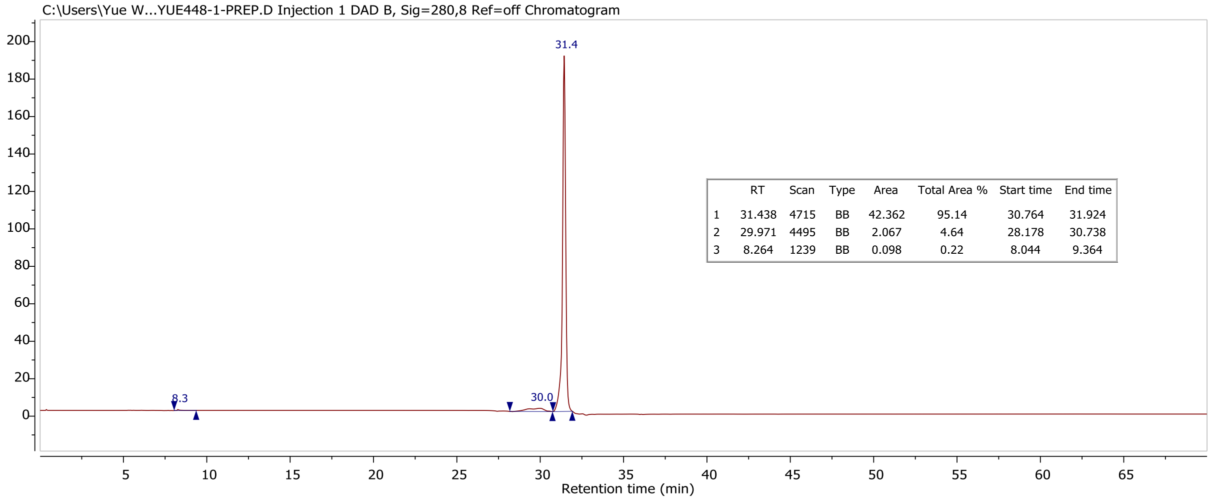

	RT	Scan	Type	Area	Total Area %	Start time	End time
1	31.438	4715	BB	42.362	95.14	30.764	31.924
2	29.971	4495	BB	2.067	4.64	28.178	30.738
3	8.264	1239	BB	0.098	0.22	8.044	9.364

Analytic HPLC of **DP_{16}-Pep_1**

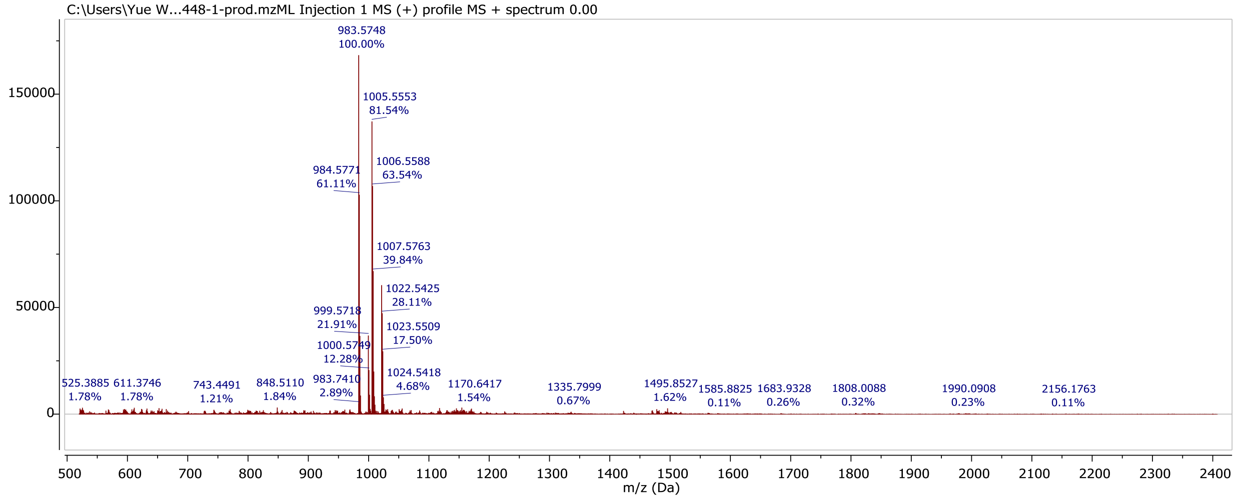

HRMS(MAIDL-TOF) of **DP_{16}-Pep_1**

DP_1-Pep_2: DP_1-HN-Ahx-YFIVFGGKRPR-COOH

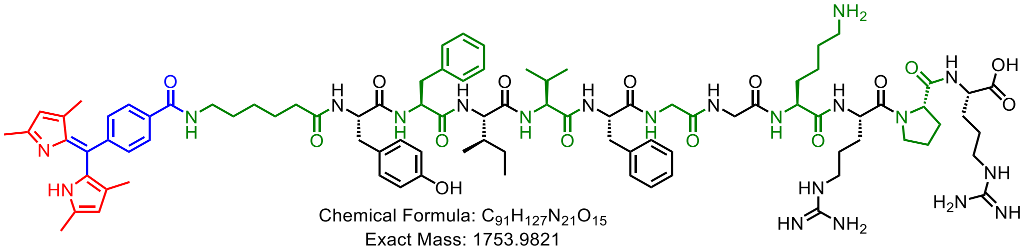

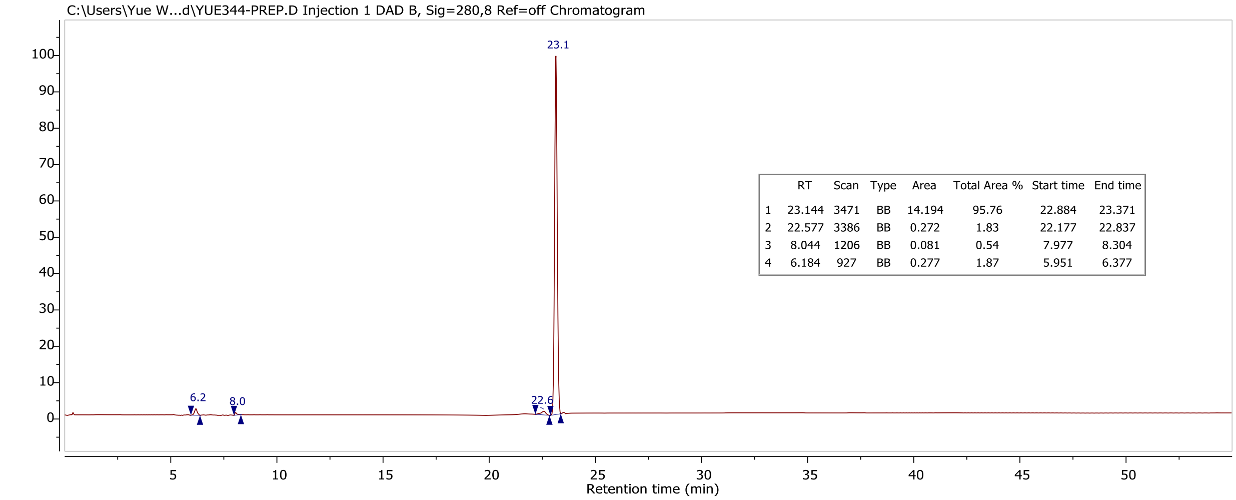

Analytic HPLC of **DP_1-Pep_2**

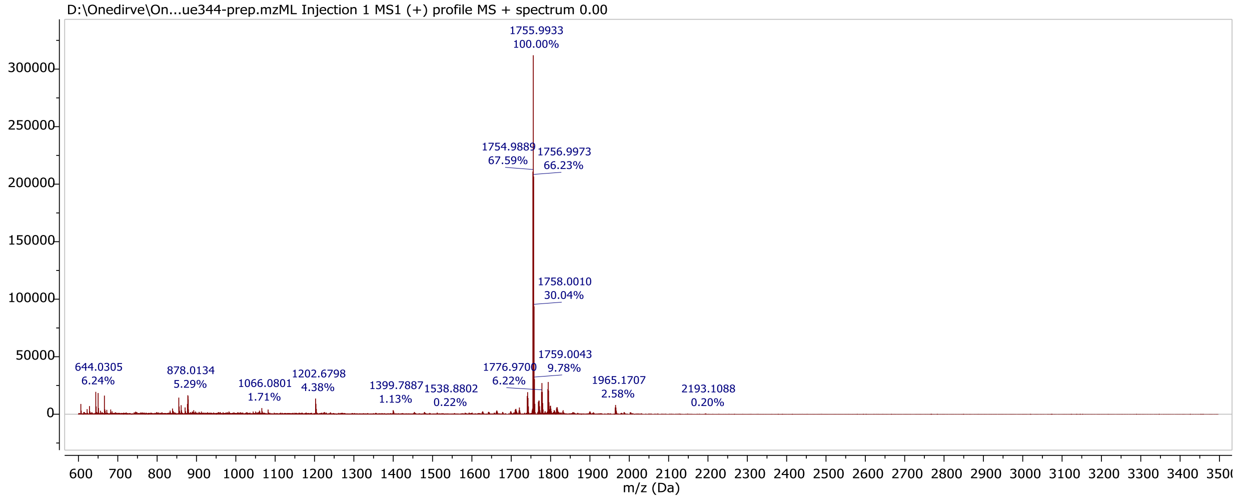

HRMS(MAIDL-TOF) of **DP_1-Pep_2**

DP_1-Pep_3: DP_1-HN-C-Ahx- RrRK-GG-YFMVF-COOH

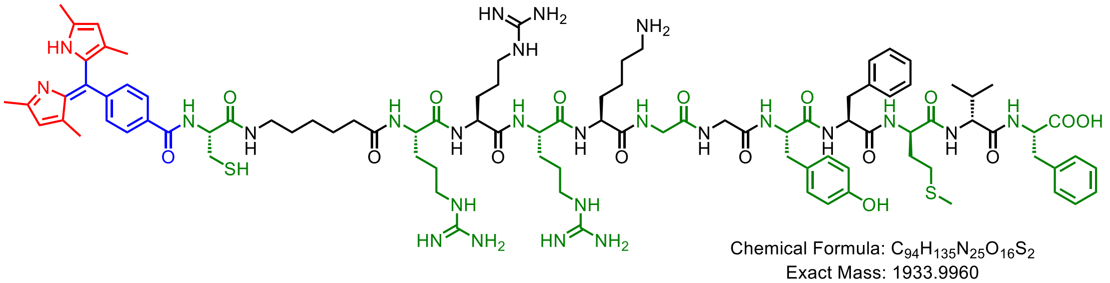

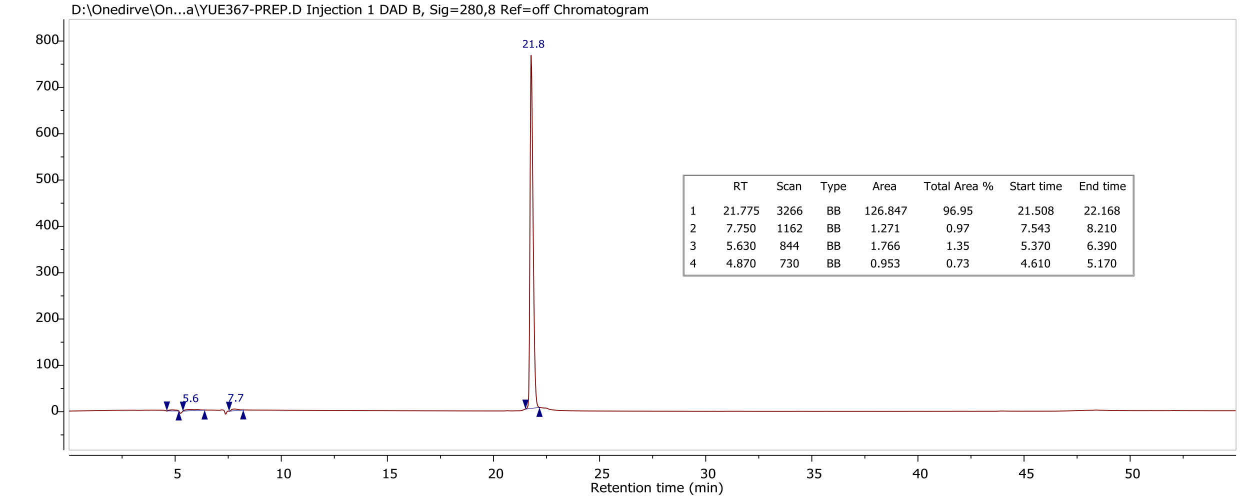

	RT	Scan	Type	Area	Total Area %	Start time	End time
1	21.775	3266	BB	126.847	96.95	21.508	22.168
2	7.750	1162	BB	1.271	0.97	7.543	8.210
3	5.630	844	BB	1.766	1.35	5.370	6.390
4	4.870	730	BB	0.953	0.73	4.610	5.170

Analytic HPLC of **DP_1-Pep_3**

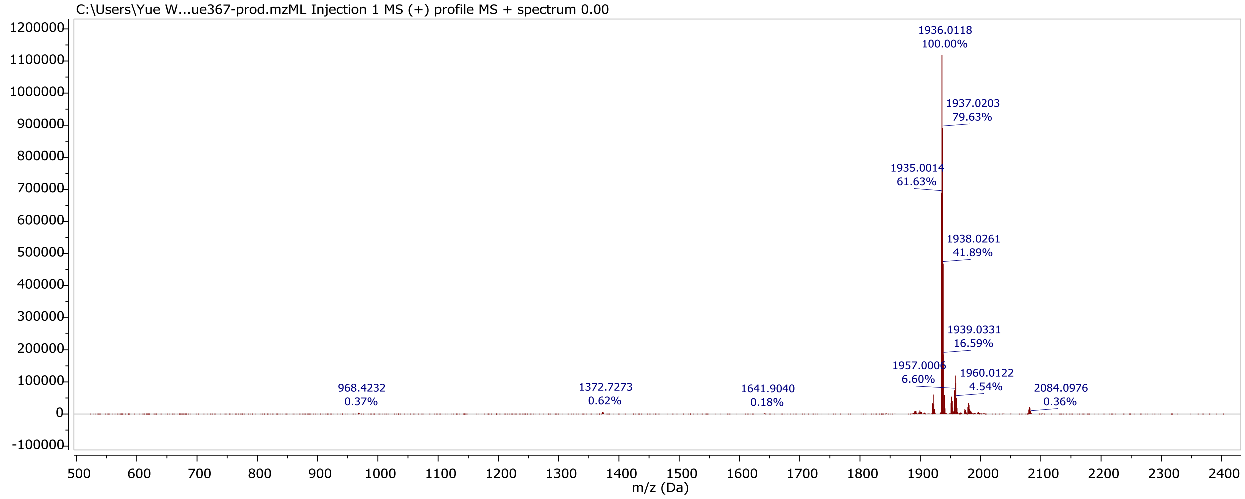

HRMS(MAIDL-TOF) of **DP_1-Pep_3**

DP_1-Pep_4: DP_1-HN-C-Ahx-YFMVF-GG-RrRK-COOH

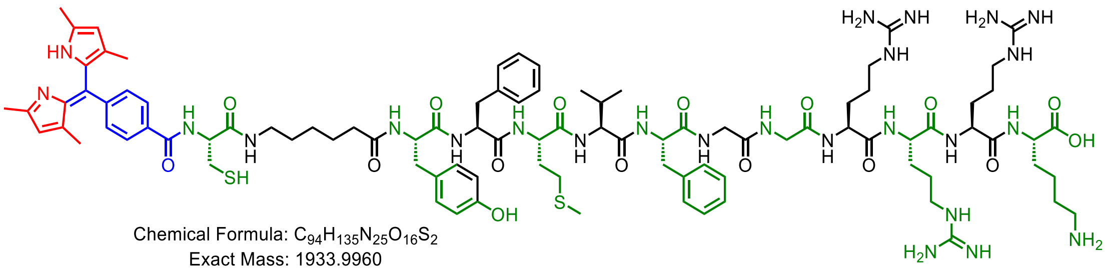

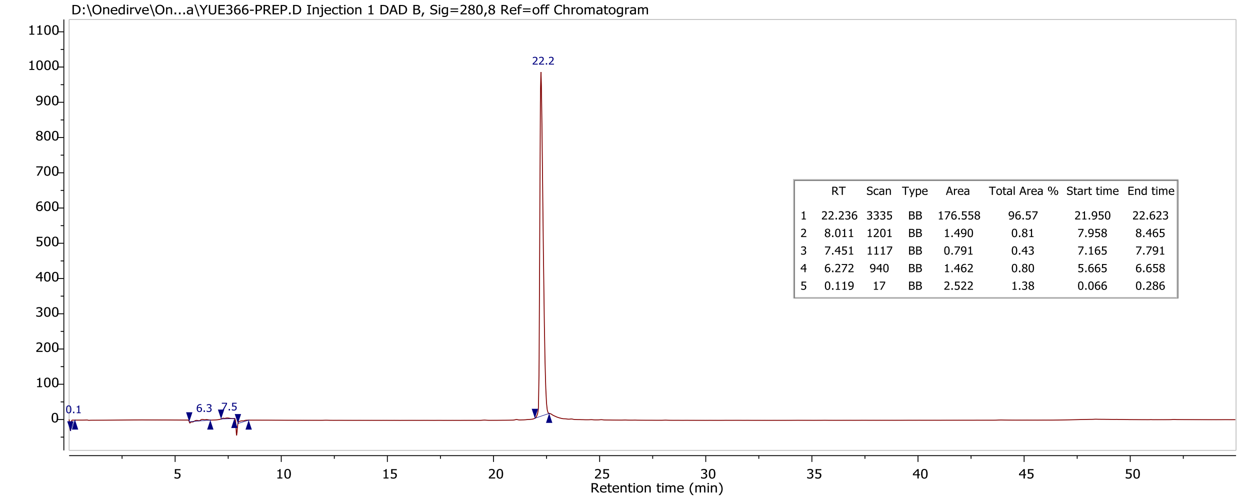

	RT	Scan	Type	Area	Total Area %	Start time	End time
1	22.236	3335	BB	176.558	96.57	21.950	22.623
2	8.011	1201	BB	1.490	0.81	7.958	8.465
3	7.451	1117	BB	0.791	0.43	7.165	7.791
4	6.272	940	BB	1.462	0.80	5.665	6.658
5	0.119	17	BB	2.522	1.38	0.066	0.286

Analytic HPLC of **DP_1-Pep_4**

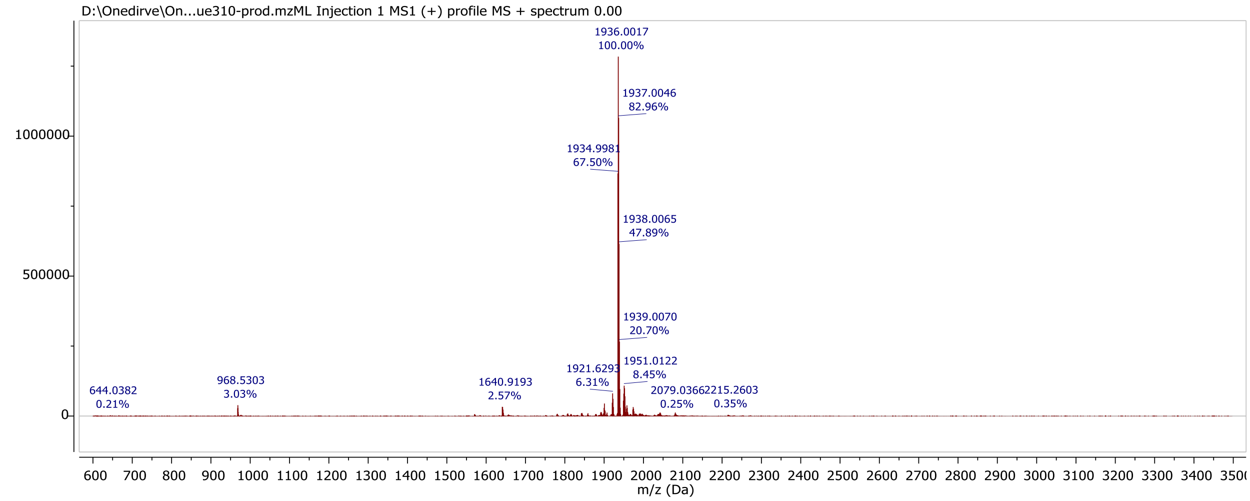

HRMS(MAIDL-TOF) of **DP_1-Pep_4**

DP_1-Pep_5: DP_1-HN-NWQYFIVF-COOH

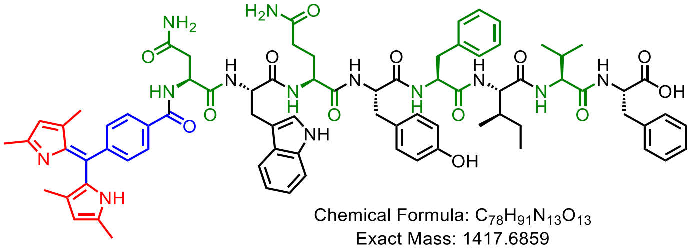

Chemical Formula: $C_{78}H_{91}N_{13}O_{13}$
Exact Mass: 1417.6859

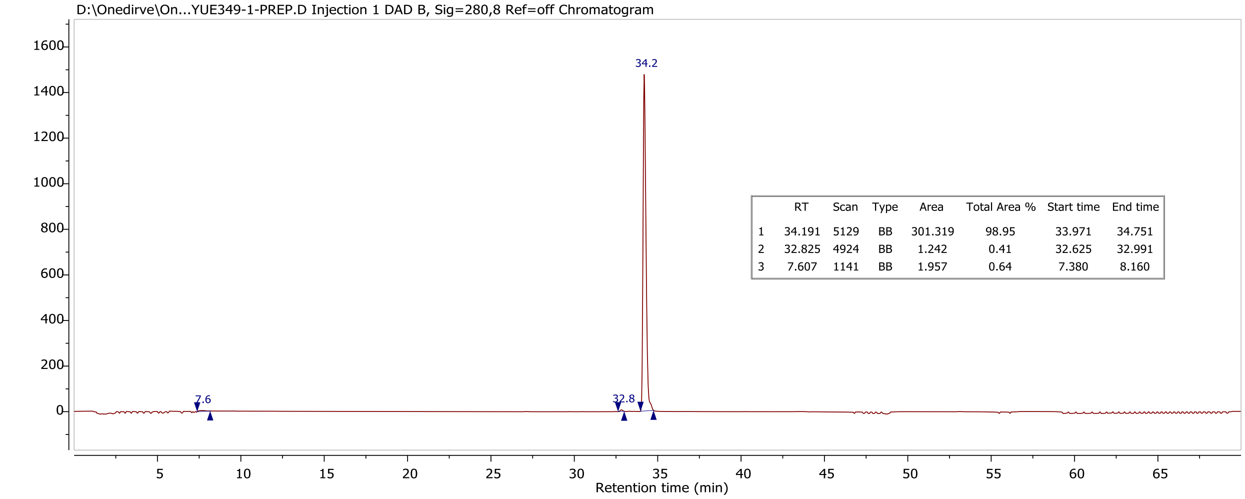

	RT	Scan	Type	Area	Total Area %	Start time	End time
1	34.191	5129	BB	301.319	98.95	33.971	34.751
2	32.825	4924	BB	1.242	0.41	32.625	32.991
3	7.607	1141	BB	1.957	0.64	7.380	8.160

Analytic HPLC of **DP_1-Pep_5**

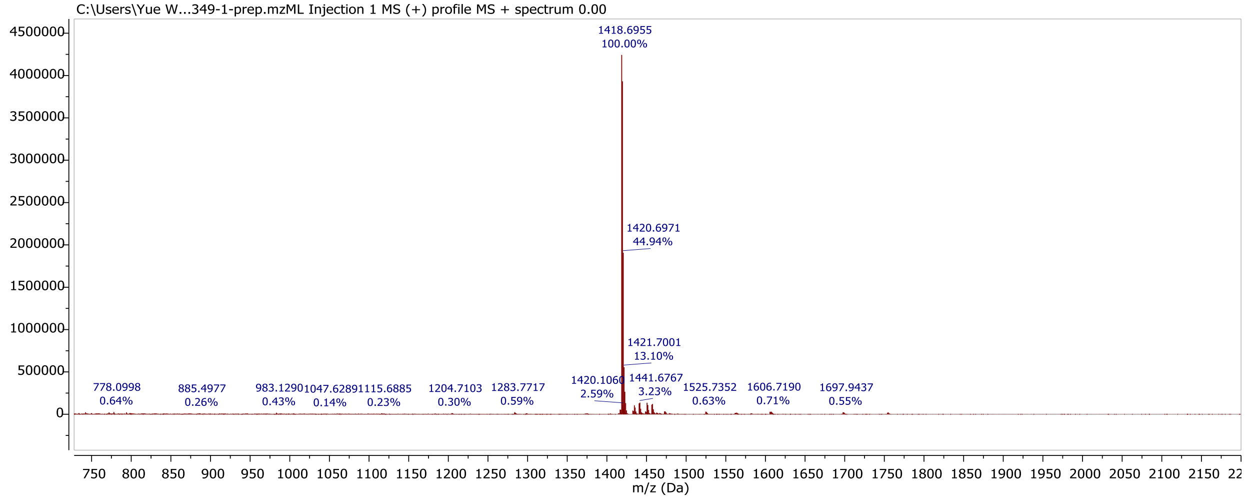

HRMS(MAIDL-TOF) of **DP_1-Pep_5**

DP_1-Pep_6: DP_1-HN-EHDYFIVF-COOH

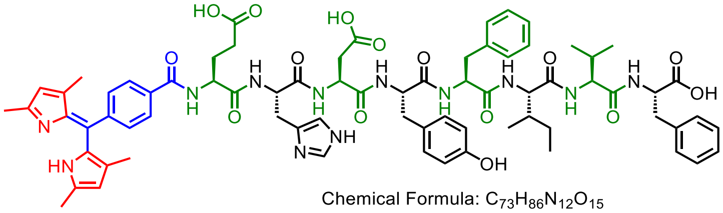

Chemical Formula: $C_{73}H_{86}N_{12}O_{15}$
Exact Mass: 1370.6336

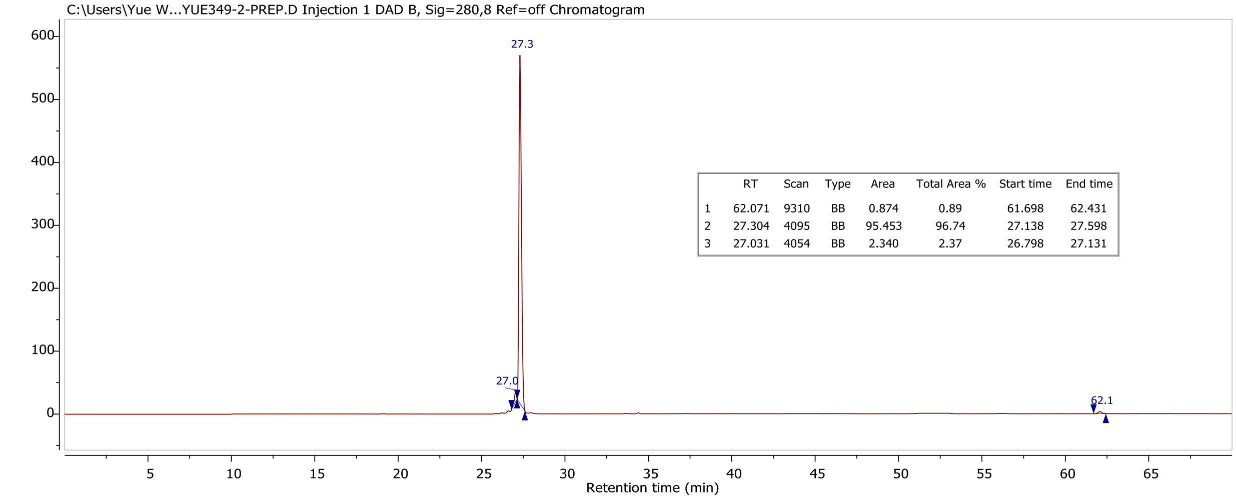

	RT	Scan	Type	Area	Total Area %	Start time	End time
1	62.071	9310	BB	0.874	0.89	61.698	62.431
2	27.304	4095	BB	95.453	96.74	27.138	27.598
3	27.031	4054	BB	2.340	2.37	26.798	27.131

Analytic HPLC of **DP_1-Pep_6**

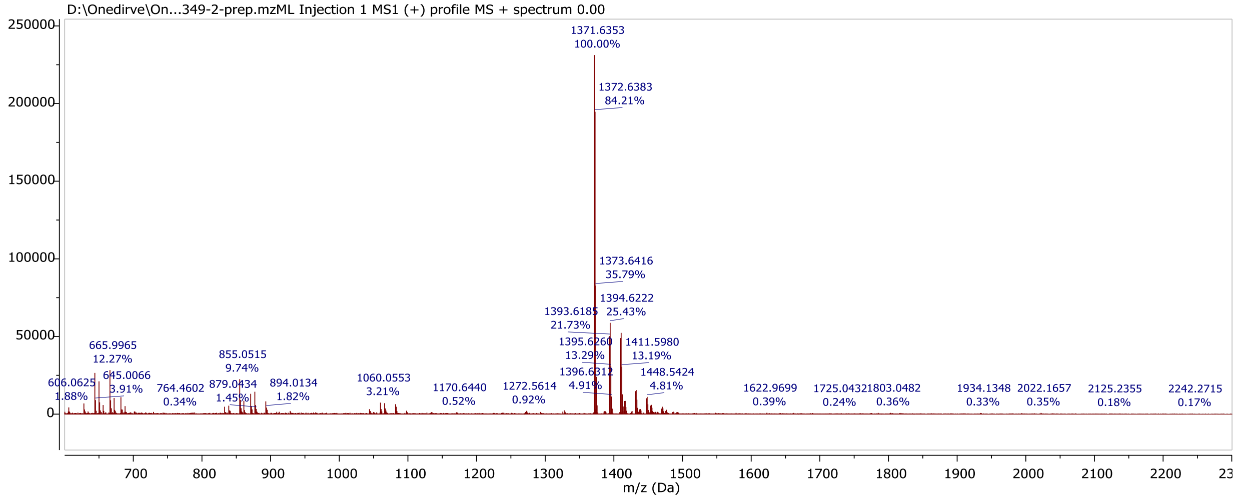

HRMS(MAIDL-TOF) of **DP_1-Pep_6**

DP$_1$-Pep$_7$: DP$_1$-HN-Ahx-P-(pTyr)-LKTK-COOH

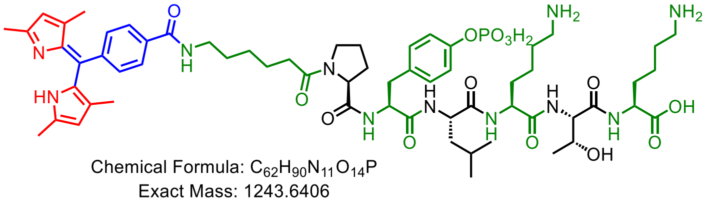

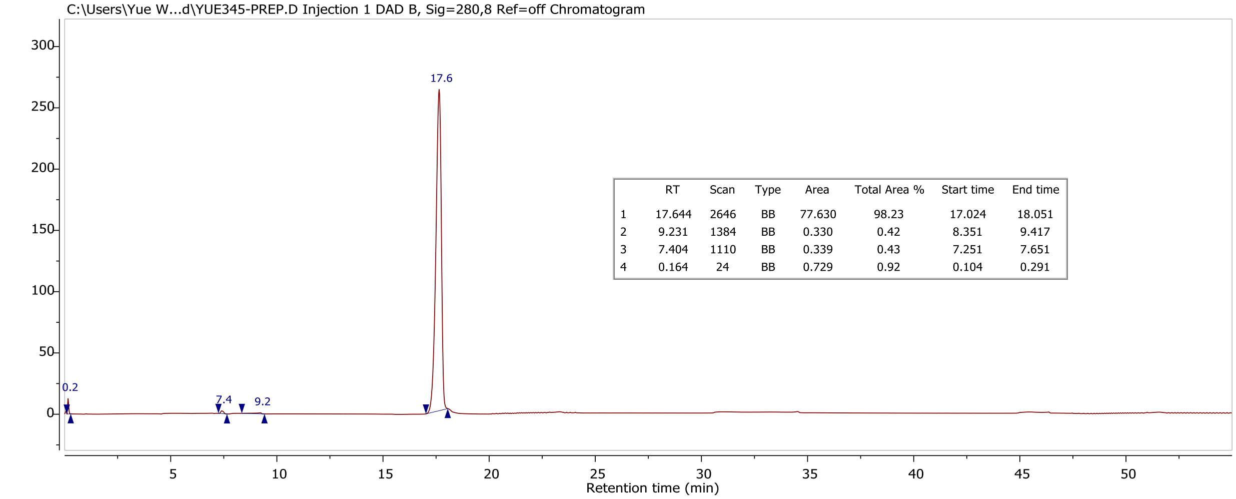

	RT	Scan	Type	Area	Total Area %	Start time	End time
1	17.644	2646	BB	77.630	98.23	17.024	18.051
2	9.231	1384	BB	0.330	0.42	8.351	9.417
3	7.404	1110	BB	0.339	0.43	7.251	7.651
4	0.164	24	BB	0.729	0.92	0.104	0.291

Analytic HPLC of **DP$_1$-Pep$_7$**

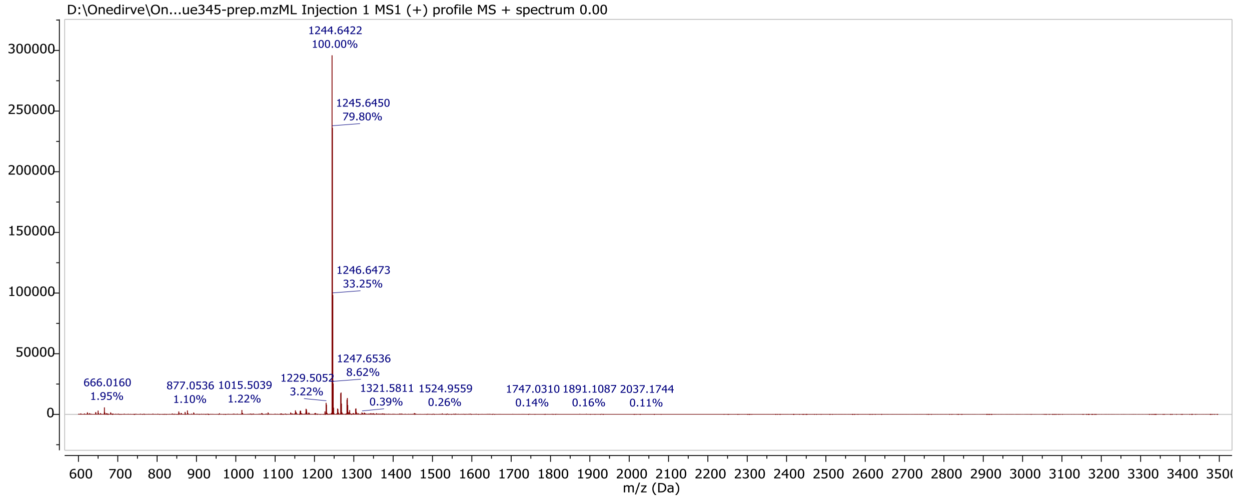

HRMS(MAIDL-TOF) of **DP$_1$-Pep$_7$**

DP_1-Pep_8: DP_1-HN-Ahx-P-(pTyr)-LKTKRrRK-COOH

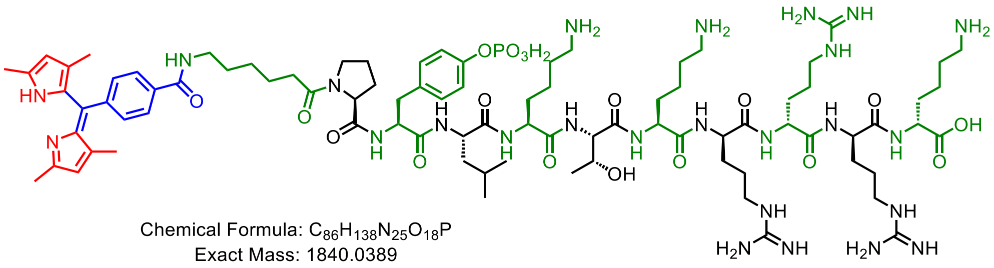

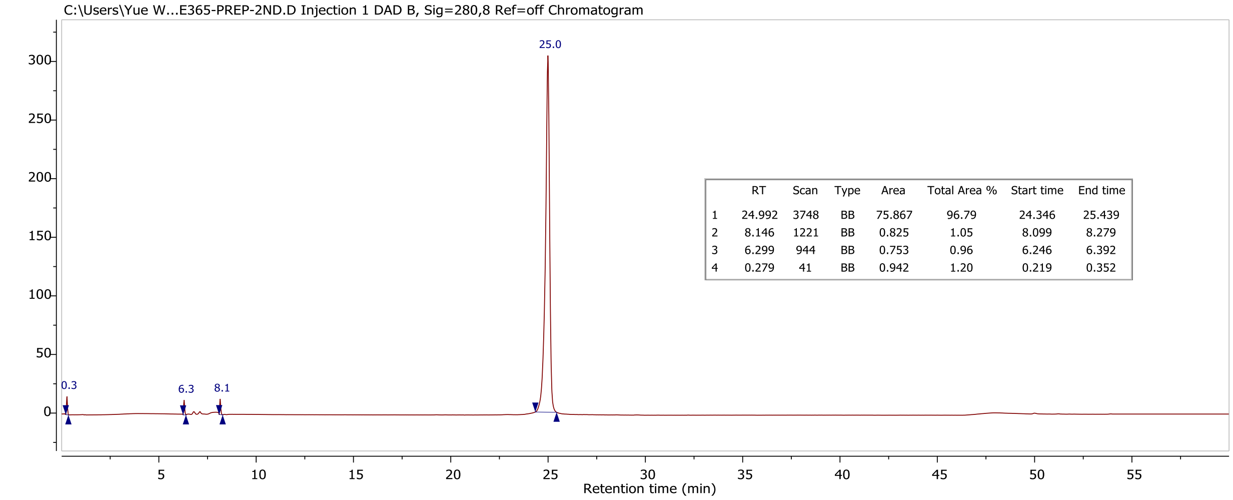

	RT	Scan	Type	Area	Total Area %	Start time	End time
1	24.992	3748	BB	75.867	96.79	24.346	25.439
2	8.146	1221	BB	0.825	1.05	8.099	8.279
3	6.299	944	BB	0.753	0.96	6.246	6.392
4	0.279	41	BB	0.942	1.20	0.219	0.352

Analytic HPLC of **DP_1-Pep_8**

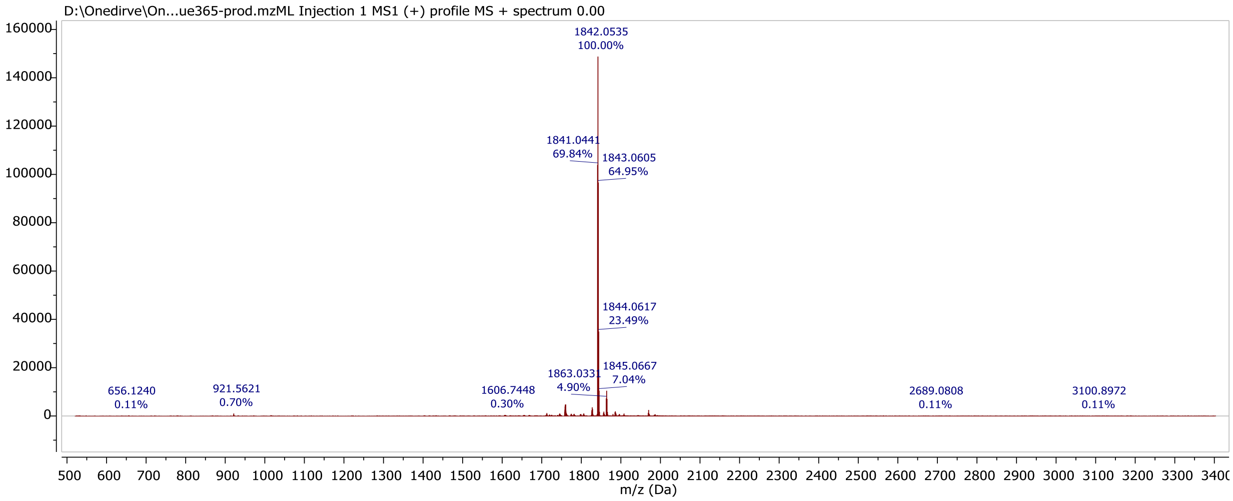

HRMS(MAIDL-TOF) of **DP_1-Pep_8**

DP_1-Pep_9: DP_1-HN-Nal-Cpa-Pal-SY-Cit-LRPG-$CONH_2$

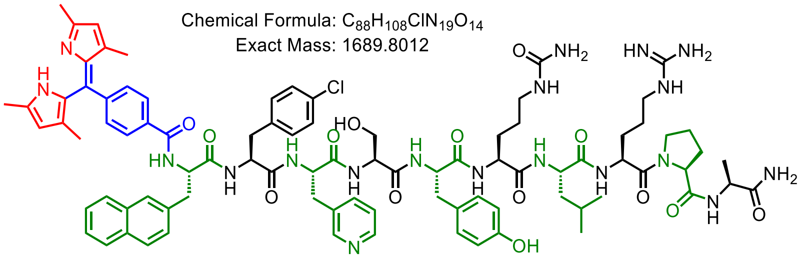

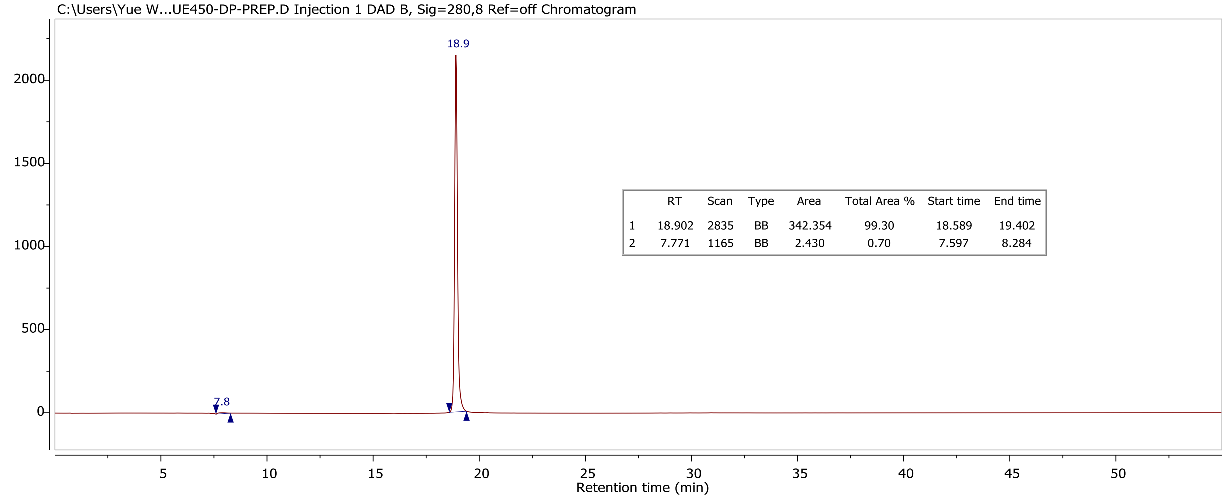

	RT	Scan	Type	Area	Total Area %	Start time	End time
1	18.902	2835	BB	342.354	99.30	18.589	19.402
2	7.771	1165	BB	2.430	0.70	7.597	8.284

Analytic HPLC of **DP_1-Pep_9**

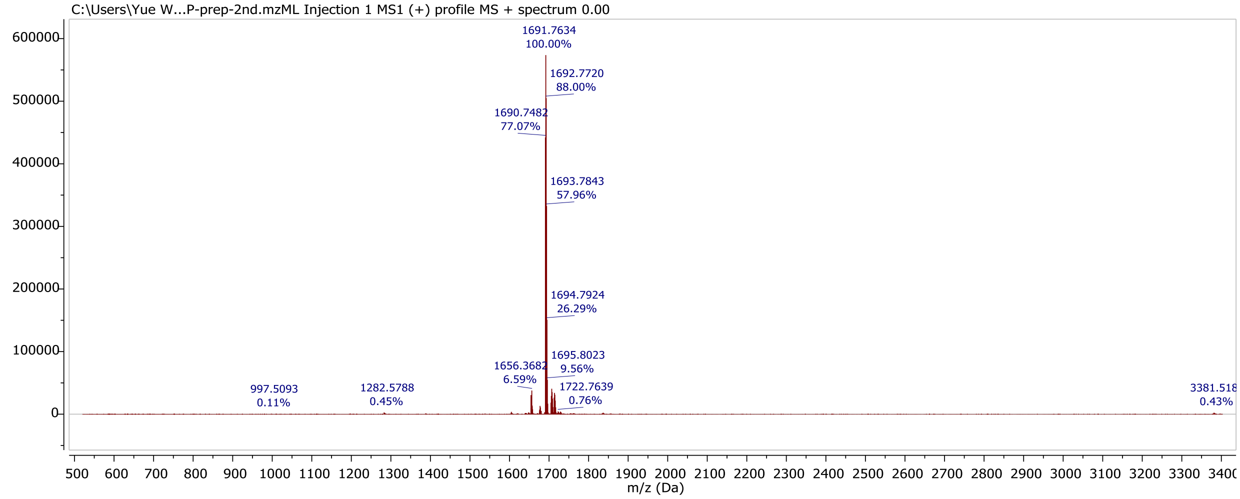

HRMS(MAIDL-TOF) of **DP_1-Pep_9**

DP_1-Pep_{10}: DP_1-HN-DRVYIHPF-$CONH_2$

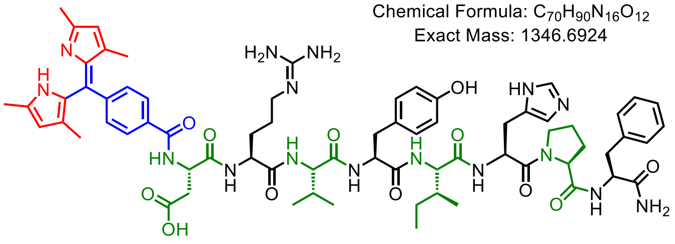

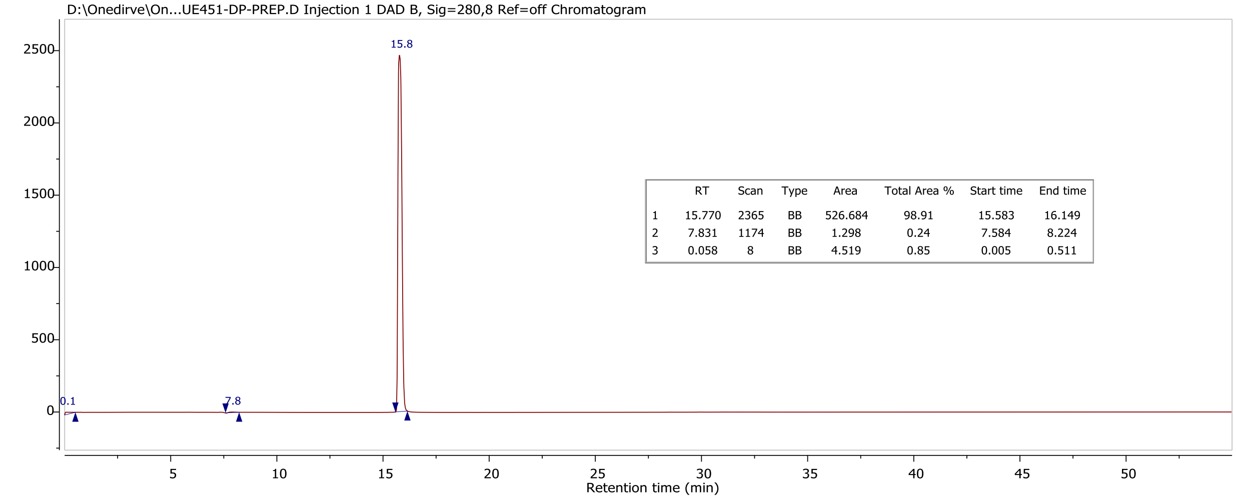

	RT	Scan	Type	Area	Total Area %	Start time	End time
1	15.770	2365	BB	526.684	98.91	15.583	16.149
2	7.831	1174	BB	1.298	0.24	7.584	8.224
3	0.058	8	BB	4.519	0.85	0.005	0.511

Analytic HPLC of **DP_1-Pep_{10}**

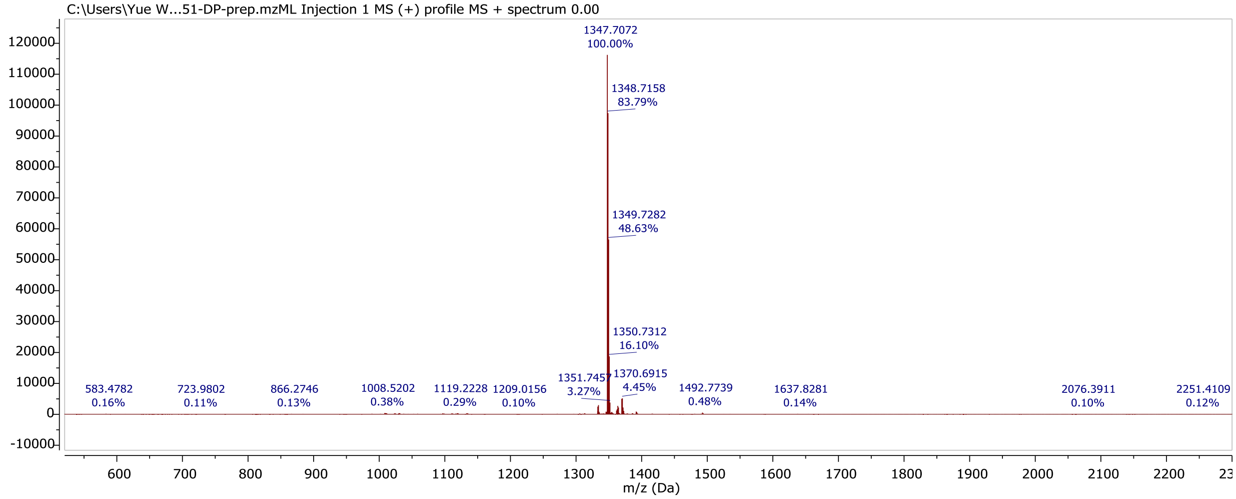

HRMS(MAIDL-TOF) of **DP_1-Pep_{10}**

DP_1-Pep_{11}: DP_1-HN-GHL-$CONH_2$

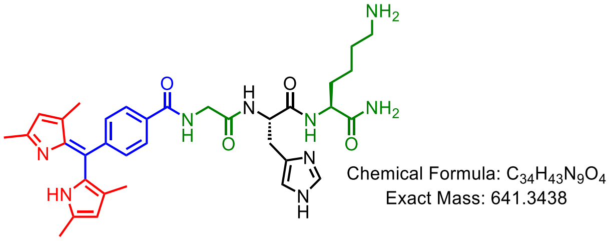

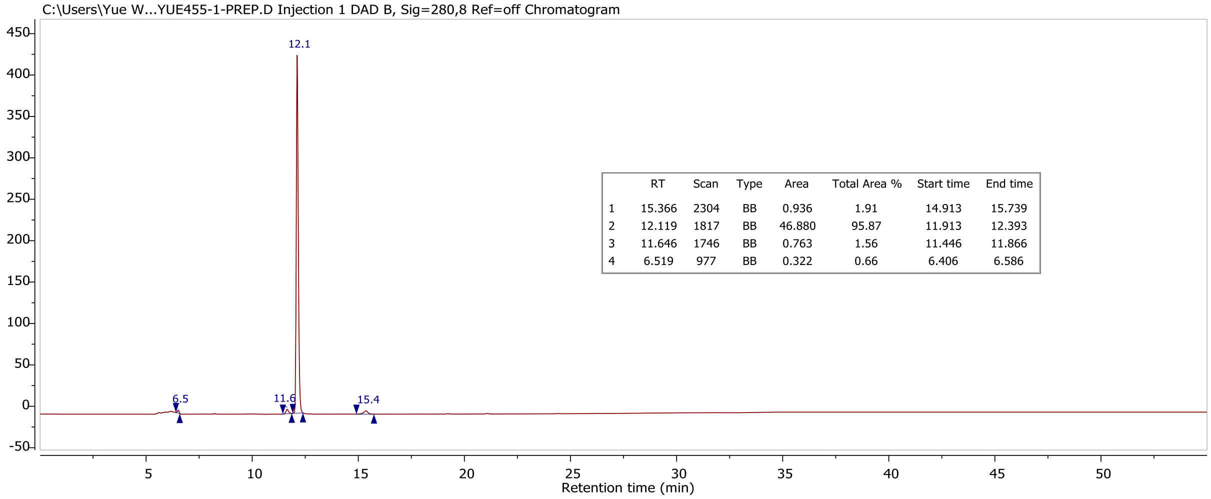

	RT	Scan	Type	Area	Total Area %	Start time	End time
1	15.366	2304	BB	0.936	1.91	14.913	15.739
2	12.119	1817	BB	46.880	95.87	11.913	12.393
3	11.646	1746	BB	0.763	1.56	11.446	11.866
4	6.519	977	BB	0.322	0.66	6.406	6.586

Analytic HPLC of **DP_1-Pep_{11}**

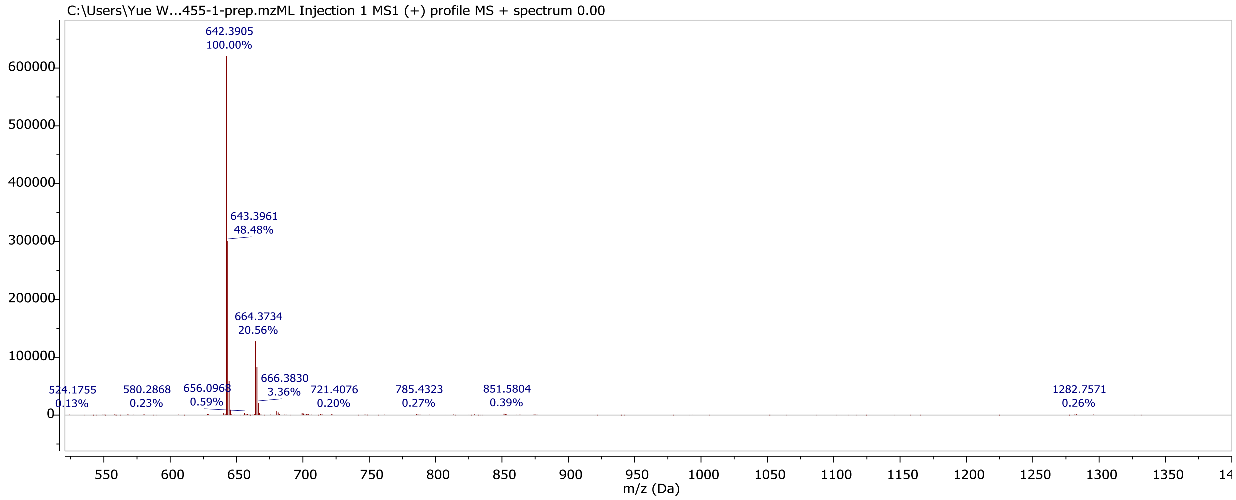

HRMS(MAIDL-TOF) of **DP_1-Pep_{11}**

DP$_1$-Pep$_{11}$*: H$_2$N-GHL(DP$_1$)-CONH$_2$

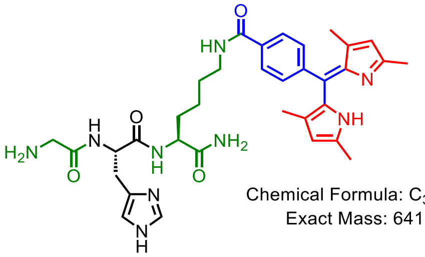

Chemical Formula: $C_{34}H_{43}N_9O_4$
Exact Mass: 641.3438

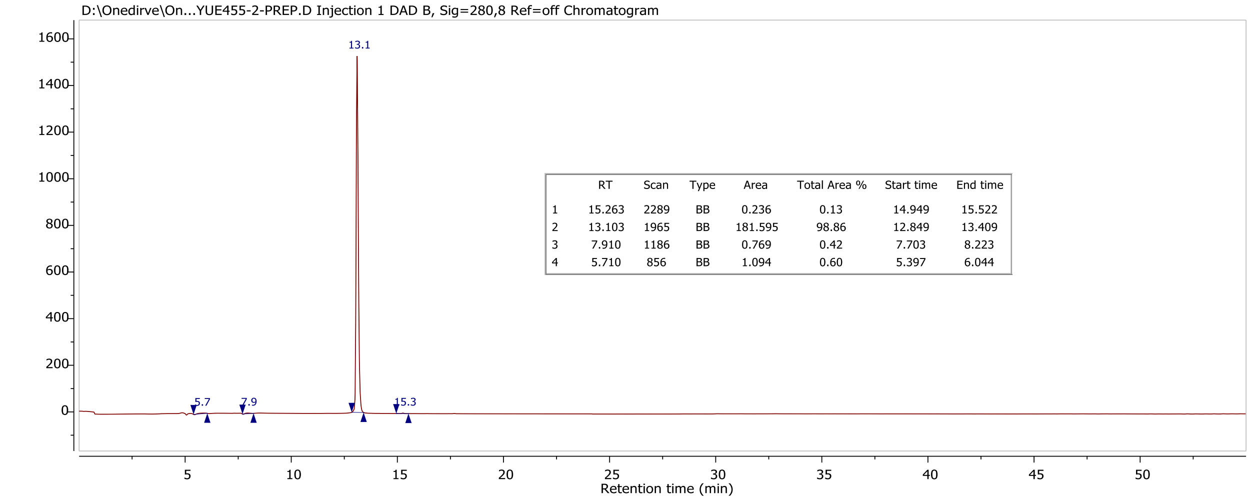

	RT	Scan	Type	Area	Total Area %	Start time	End time
1	15.263	2289	BB	0.236	0.13	14.949	15.522
2	13.103	1965	BB	181.595	98.86	12.849	13.409
3	7.910	1186	BB	0.769	0.42	7.703	8.223
4	5.710	856	BB	1.094	0.60	5.397	6.044

Analytic HPLC of **DP$_1$-Pep$_{11}$***

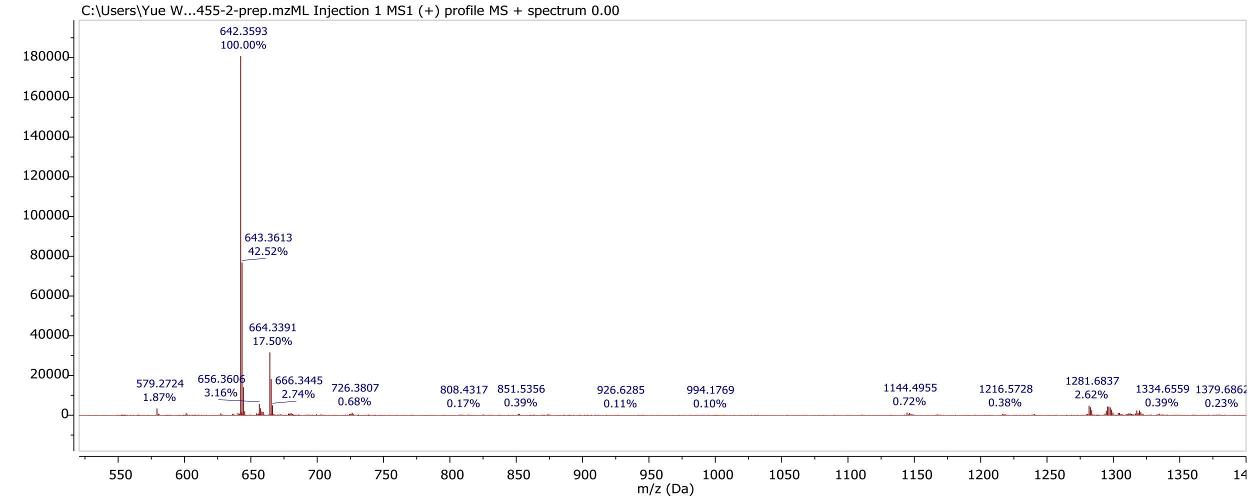

HRMS(MAIDL-TOF) of **DP$_1$-Pep$_{11}$***

BODIPY$_1$-Pep$_1$: BODIPY$_1$-NH-YFMVF-CONH$_2$

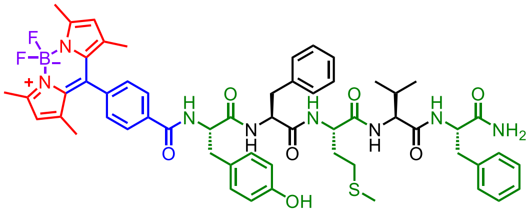

Chemical Formula: $C_{57}H_{65}BF_2N_8O_7S$
Exact Mass: 1054.4758

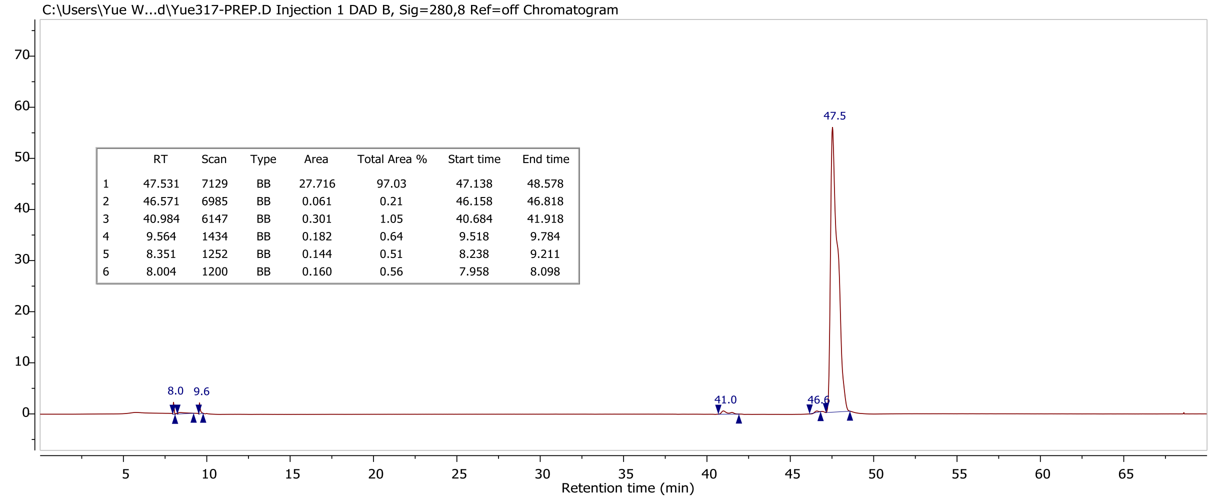

	RT	Scan	Type	Area	Total Area %	Start time	End time
1	47.531	7129	BB	27.716	97.03	47.138	48.578
2	46.571	6985	BB	0.061	0.21	46.158	46.818
3	40.984	6147	BB	0.301	1.05	40.684	41.918
4	9.564	1434	BB	0.182	0.64	9.518	9.784
5	8.351	1252	BB	0.144	0.51	8.238	9.211
6	8.004	1200	BB	0.160	0.56	7.958	8.098

Analytic HPLC of **BODIPY$_1$-Pep$_1$**

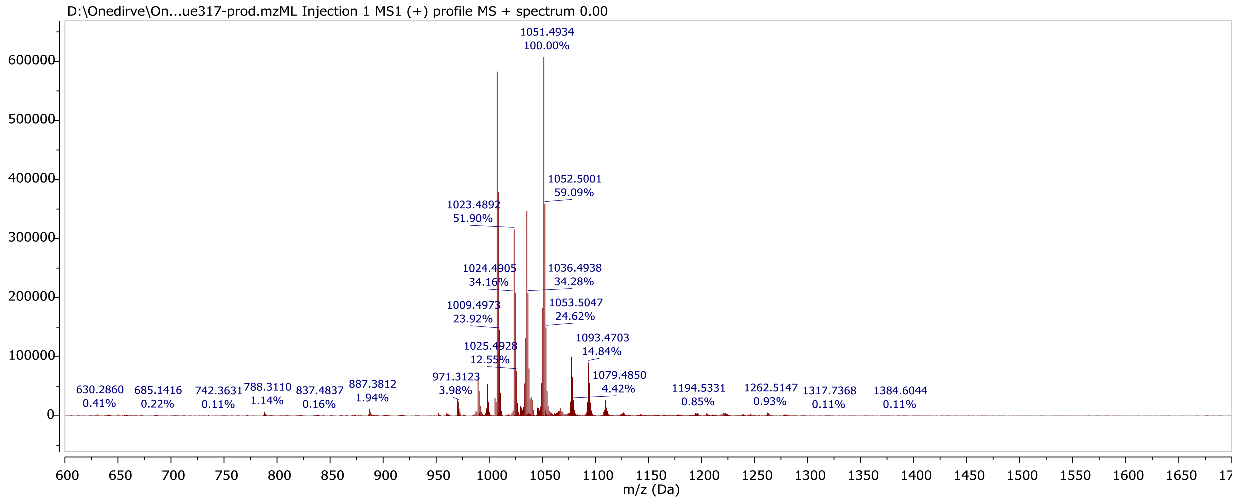

HRMS(MAIDL-TOF) of **BODIPY$_1$-Pep$_1$**

BODIPY$_2$-Pep$_1$: BODIPY$_2$-NH-YFMVF-CONH$_2$

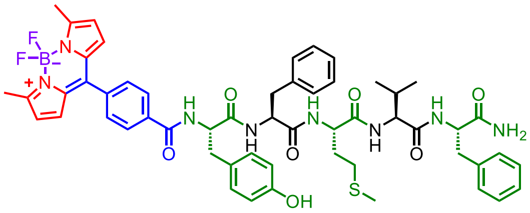

Chemical Formula: $C_{55}H_{61}BF_2N_8O_7S$
Exact Mass: 1026.4445

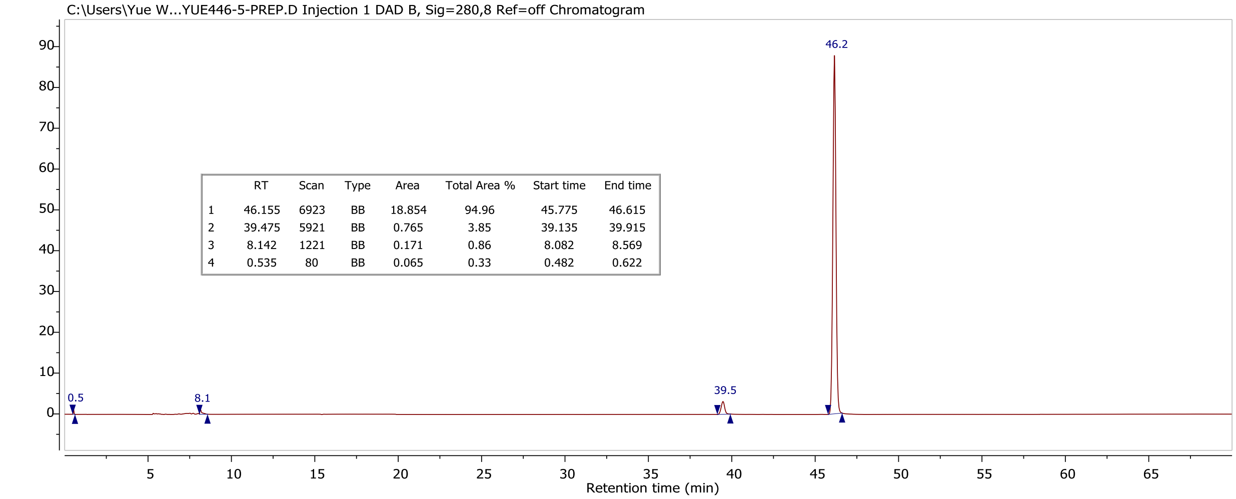

	RT	Scan	Type	Area	Total Area %	Start time	End time
1	46.155	6923	BB	18.854	94.96	45.775	46.615
2	39.475	5921	BB	0.765	3.85	39.135	39.915
3	8.142	1221	BB	0.171	0.86	8.082	8.569
4	0.535	80	BB	0.065	0.33	0.482	0.622

Analytic HPLC of **BODIPY$_2$-Pep$_1$**

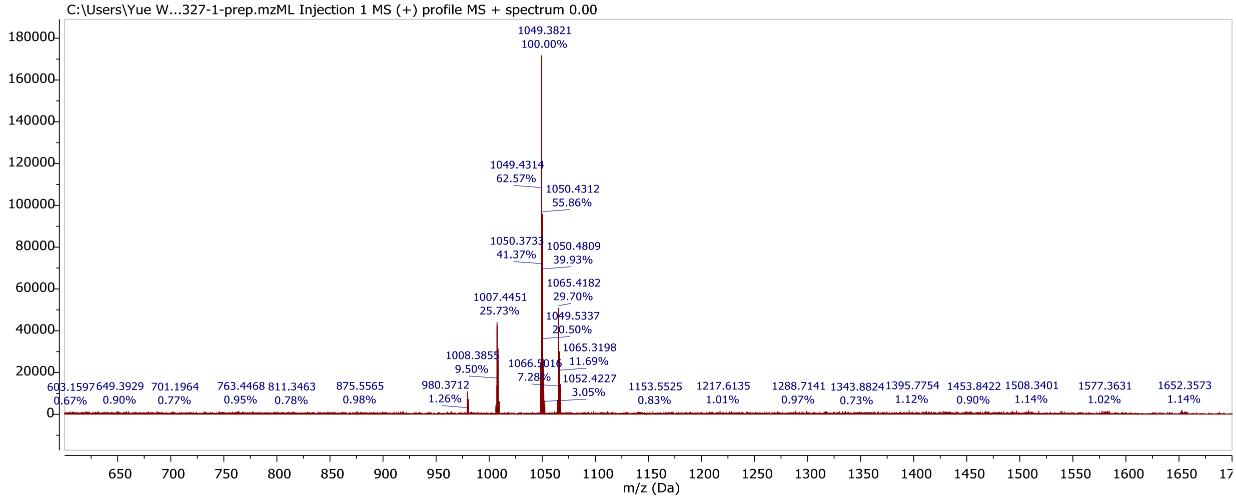

HRMS(MAIDL-TOF) of **BODIPY$_2$-Pep$_1$**

BODIPY$_3$-Pep$_1$: BODIPY$_3$-NH-YFMVF-CONH$_2$

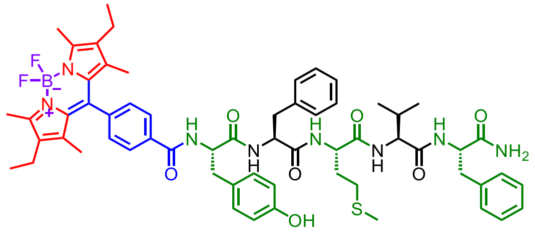

Chemical Formula: $C_{61}H_{73}BF_2N_8O_7S$

Exact Mass: 1110.5384

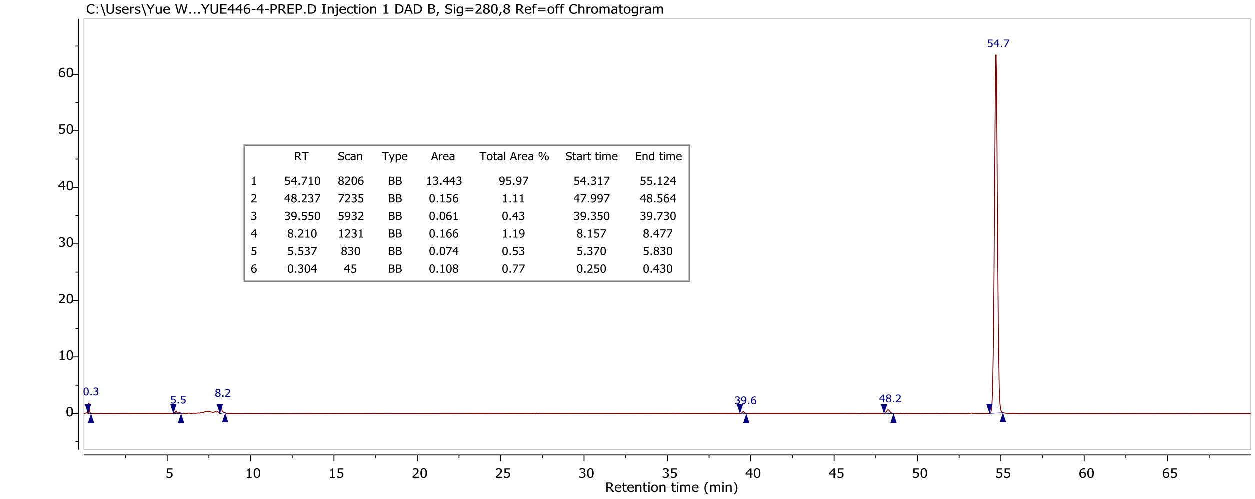

	RT	Scan	Type	Area	Total Area %	Start time	End time
1	54.710	8206	BB	13.443	95.97	54.317	55.124
2	48.237	7235	BB	0.156	1.11	47.997	48.564
3	39.550	5932	BB	0.061	0.43	39.350	39.730
4	8.210	1231	BB	0.166	1.19	8.157	8.477
5	5.537	830	BB	0.074	0.53	5.370	5.830
6	0.304	45	BB	0.108	0.77	0.250	0.430

Analytic HPLC of **BODIPY$_3$-Pep$_1$**

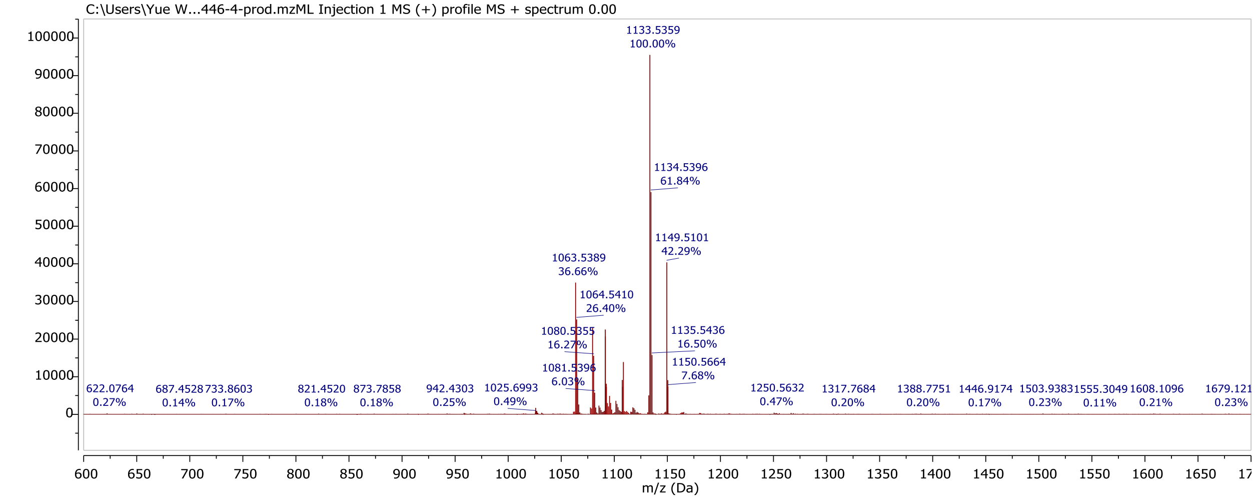

HRMS(MAIDL-TOF) of **BODIPY$_3$-Pep$_1$**

BODIPY$_9$-Pep$_1$: BODIPY$_9$-NH-YFMVF-CONH$_2$

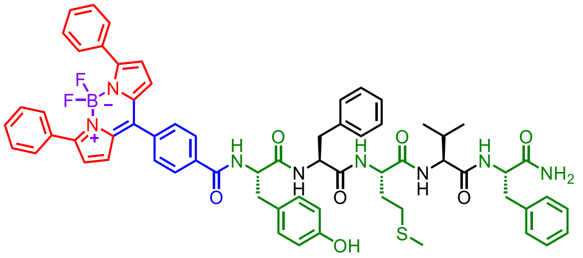

Chemical Formula: $C_{65}H_{65}BF_2N_8O_7S$

Exact Mass: 1150.4758

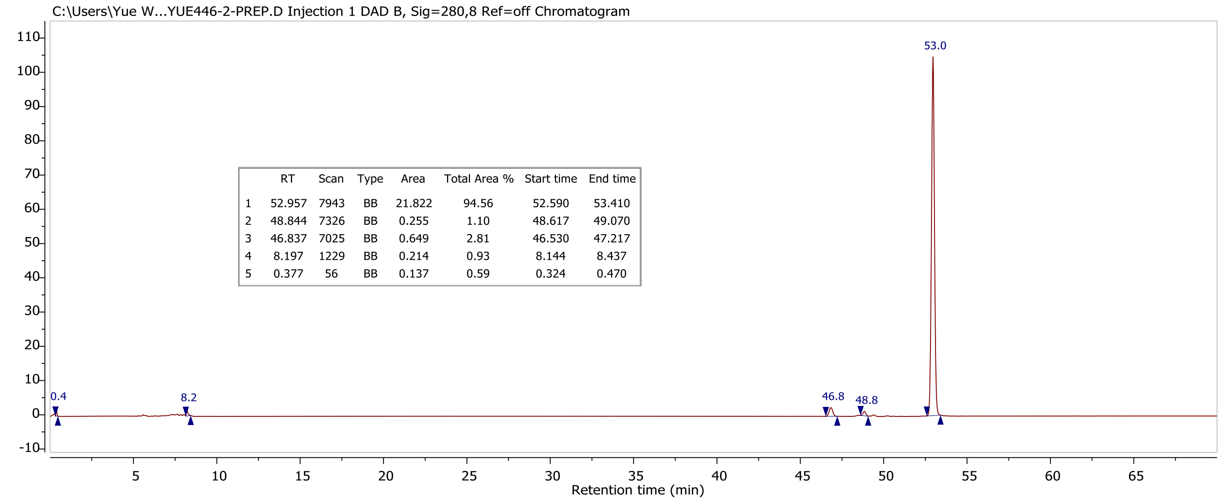

	RT	Scan	Type	Area	Total Area %	Start time	End time
1	52.957	7943	BB	21.822	94.56	52.590	53.410
2	48.844	7326	BB	0.255	1.10	48.617	49.070
3	46.837	7025	BB	0.649	2.81	46.530	47.217
4	8.197	1229	BB	0.214	0.93	8.144	8.437
5	0.377	56	BB	0.137	0.59	0.324	0.470

Analytic HPLC of **BODIPY$_9$-Pep$_1$**

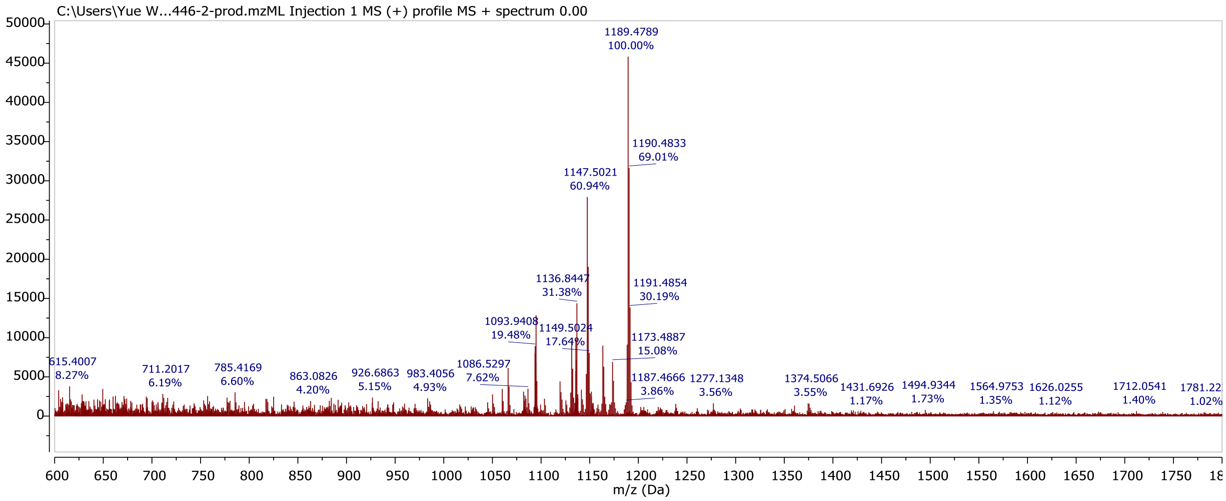

HRMS(MAIDL-TOF) of **BODIPY$_9$-Pep$_1$**

BODIPY$_{15}$-Pep$_1$: BODIPY$_{15}$-NH-YFMVF-CONH$_2$

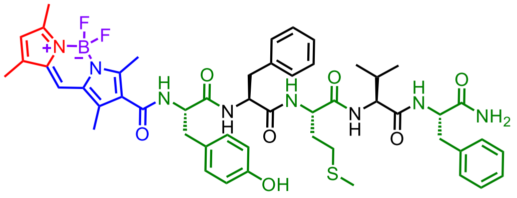

Chemical Formula: $C_{51}H_{61}BF_2N_8O_7S$
Exact Mass: 978.4445

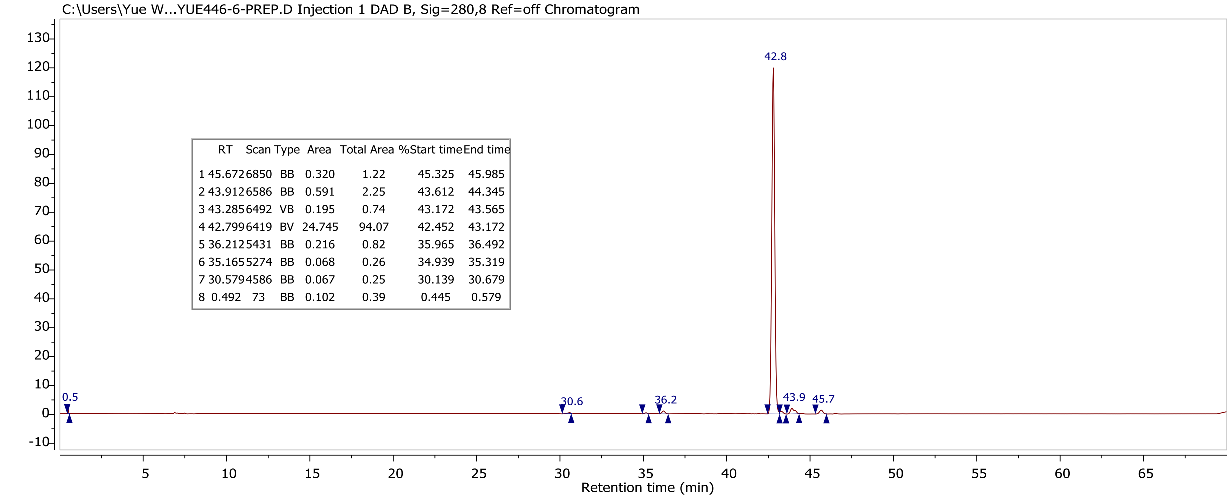

Analytic HPLC of **BODIPY$_{15}$-Pep$_1$**

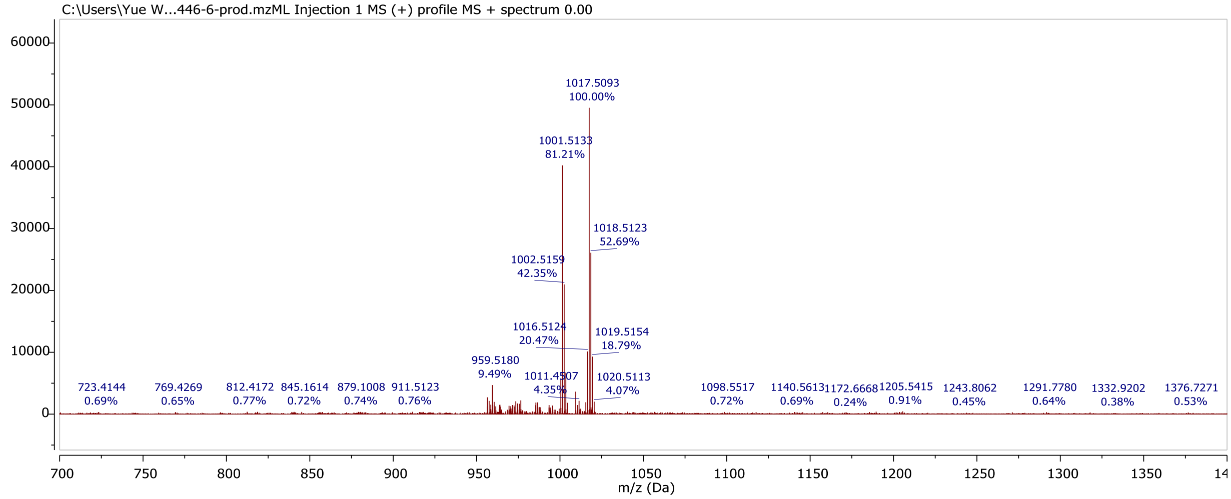

HRMS(MAIDL-TOF) of **BODIPY$_{15}$-Pep$_1$**

BODIPY$_1$-Pep$_4$: BODIPY$_1$-HN-C-Ahx-YFMVF-GG-RrRK-COOH

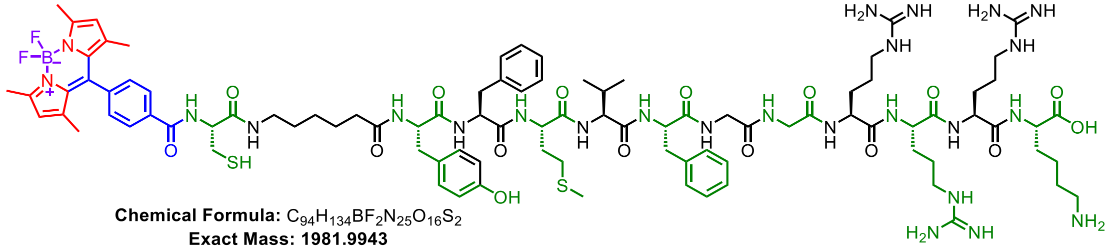

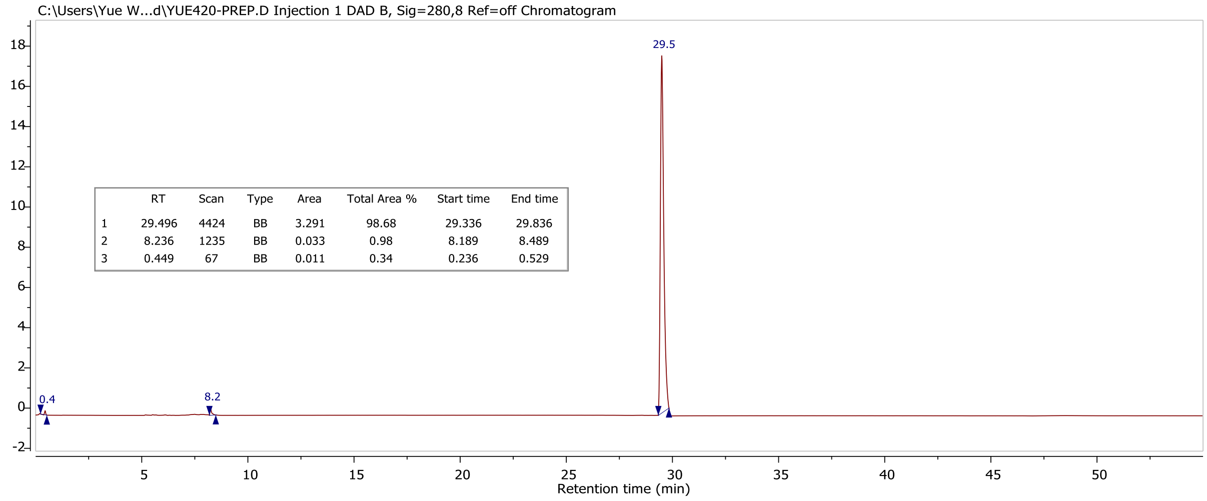

	RT	Scan	Type	Area	Total Area %	Start time	End time
1	29.496	4424	BB	3.291	98.68	29.336	29.836
2	8.236	1235	BB	0.033	0.98	8.189	8.489
3	0.449	67	BB	0.011	0.34	0.236	0.529

Analytic HPLC of **BODIPY$_1$-Pep$_4$**

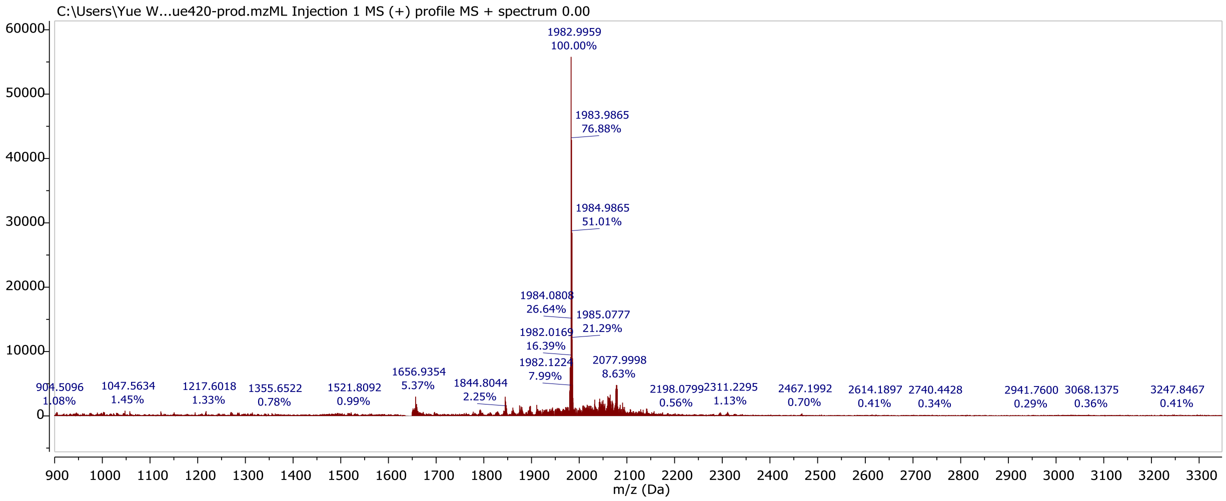

HRMS(MAIDL-TOF) of **BODIPY$_1$-Pep$_4$**

BODIPY$_1$-Pep$_5$: BODIPY$_1$-HN-NWQYFIVF-COOH

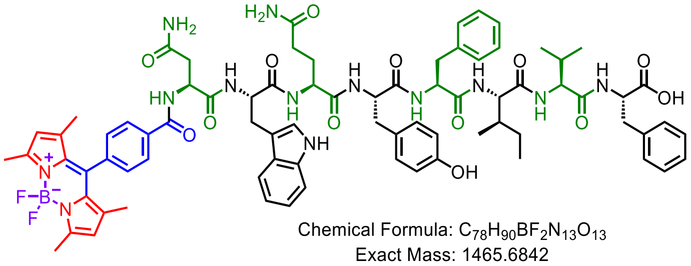

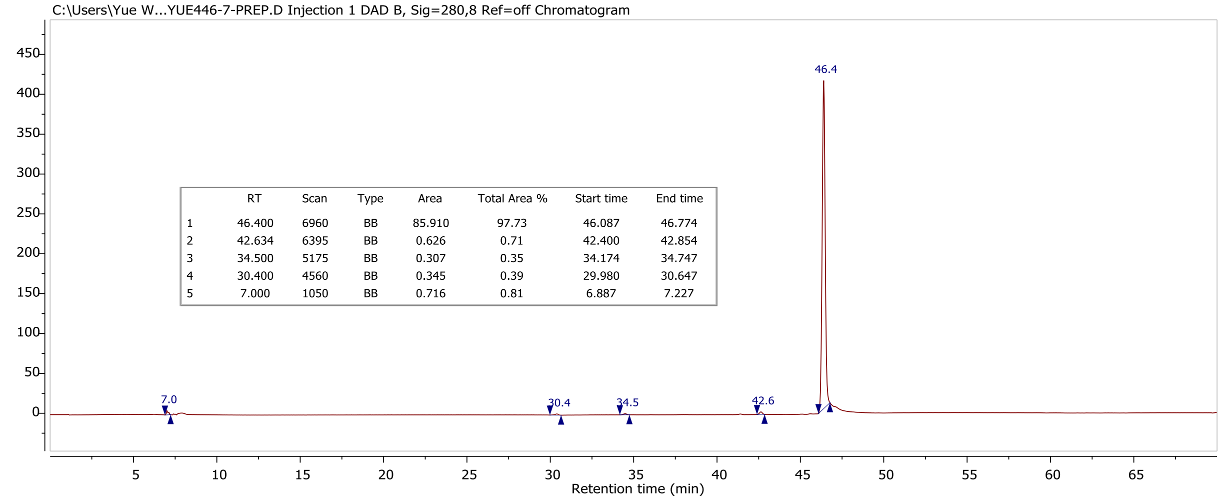

	RT	Scan	Type	Area	Total Area %	Start time	End time
1	46.400	6960	BB	85.910	97.73	46.087	46.774
2	42.634	6395	BB	0.626	0.71	42.400	42.854
3	34.500	5175	BB	0.307	0.35	34.174	34.747
4	30.400	4560	BB	0.345	0.39	29.980	30.647
5	7.000	1050	BB	0.716	0.81	6.887	7.227

Analytic HPLC of **BODIPY$_1$-Pep$_5$**

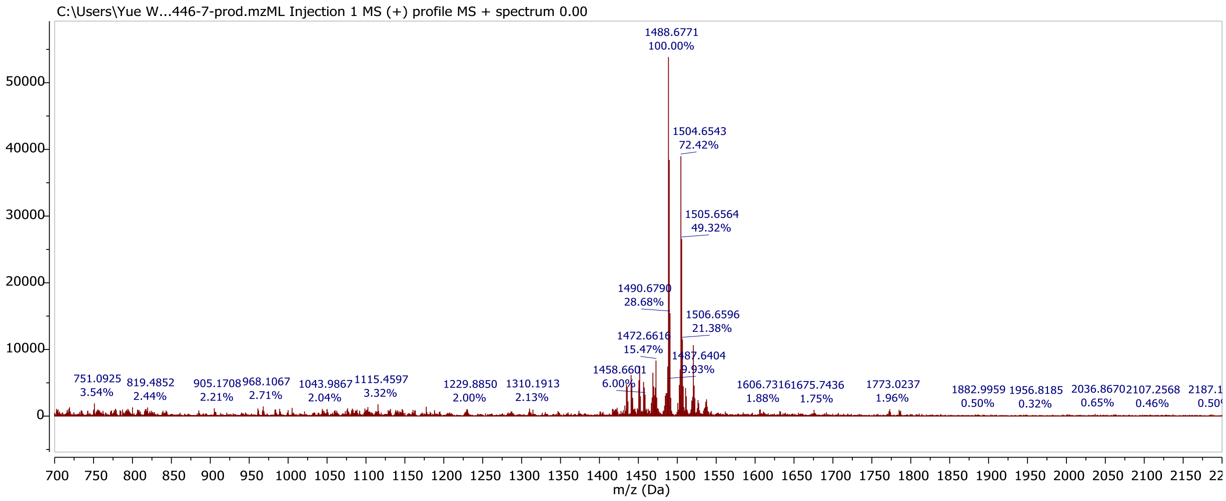

HRMS(MAIDL-TOF) of **BODIPY$_1$-Pep$_5$**

BODIPY$_1$-Pep$_6$: BODIPY$_1$-HN-EHDYFIVF-COOH

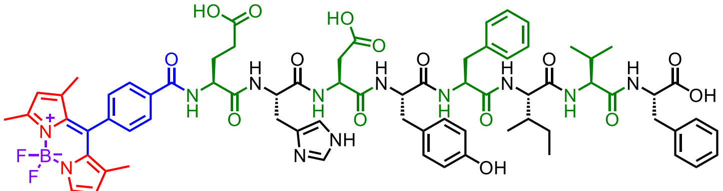

Chemical Formula: $C_{73}H_{85}BF_2N_{12}O_{15}$
Exact Mass: 1418.6318

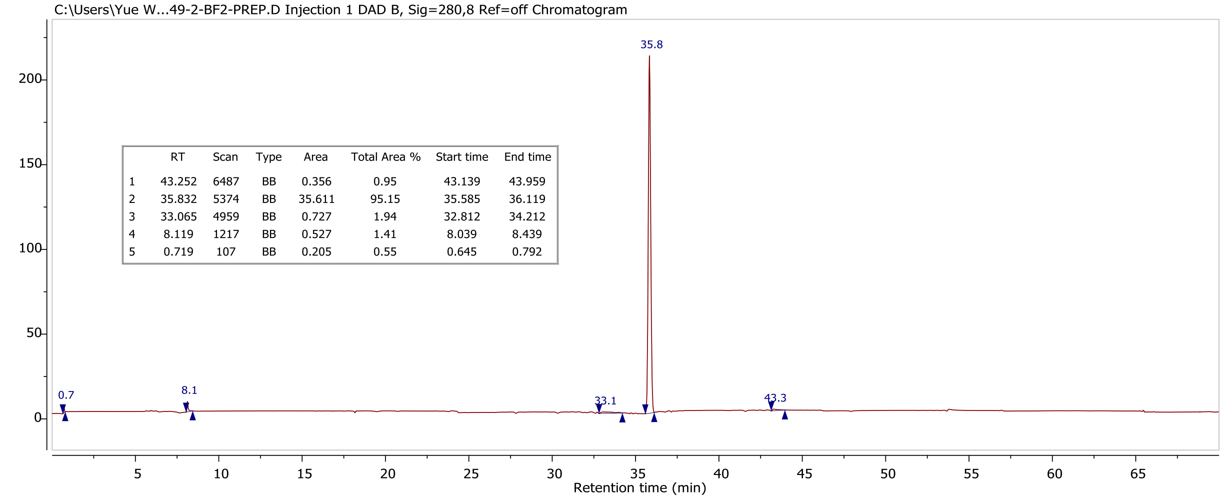

	RT	Scan	Type	Area	Total Area %	Start time	End time
1	43.252	6487	BB	0.356	0.95	43.139	43.959
2	35.832	5374	BB	35.611	95.15	35.585	36.119
3	33.065	4959	BB	0.727	1.94	32.812	34.212
4	8.119	1217	BB	0.527	1.41	8.039	8.439
5	0.719	107	BB	0.205	0.55	0.645	0.792

Analytic HPLC of **BODIPY$_1$-Pep$_6$**

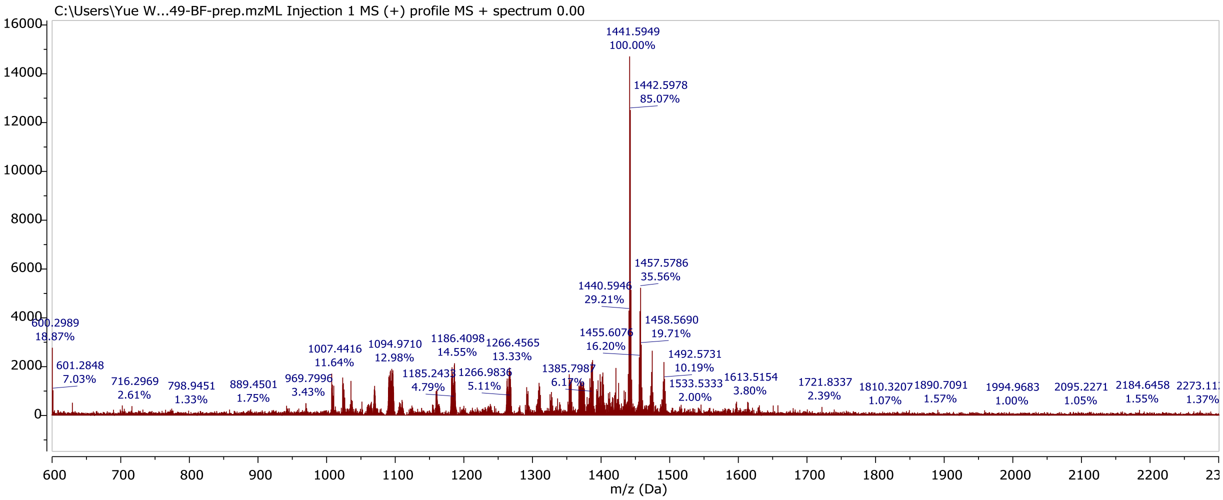

HRMS(MAIDL-TOF) of **BODIPY$_1$-Pep$_6$**

BODIPY$_1$-Pep$_7$: DP$_1$-HN-Ahx-P-(pTyr)-LKTK-COOH

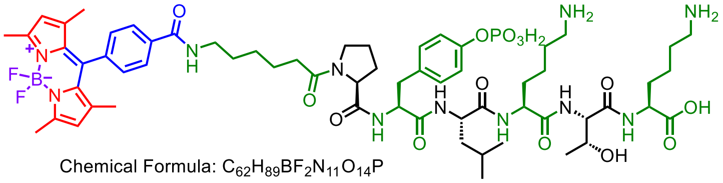

Chemical Formula: $C_{62}H_{89}BF_2N_{11}O_{14}P$
Exact Mass: 1291.6389

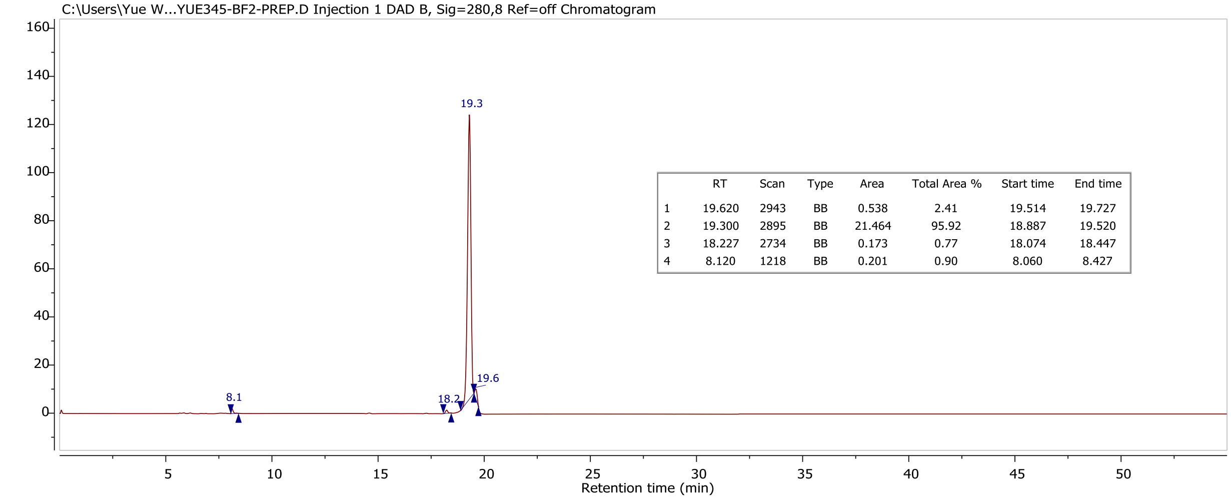

	RT	Scan	Type	Area	Total Area %	Start time	End time
1	19.620	2943	BB	0.538	2.41	19.514	19.727
2	19.300	2895	BB	21.464	95.92	18.887	19.520
3	18.227	2734	BB	0.173	0.77	18.074	18.447
4	8.120	1218	BB	0.201	0.90	8.060	8.427

Analytic HPLC of **BODIPY$_1$-Pep$_7$**

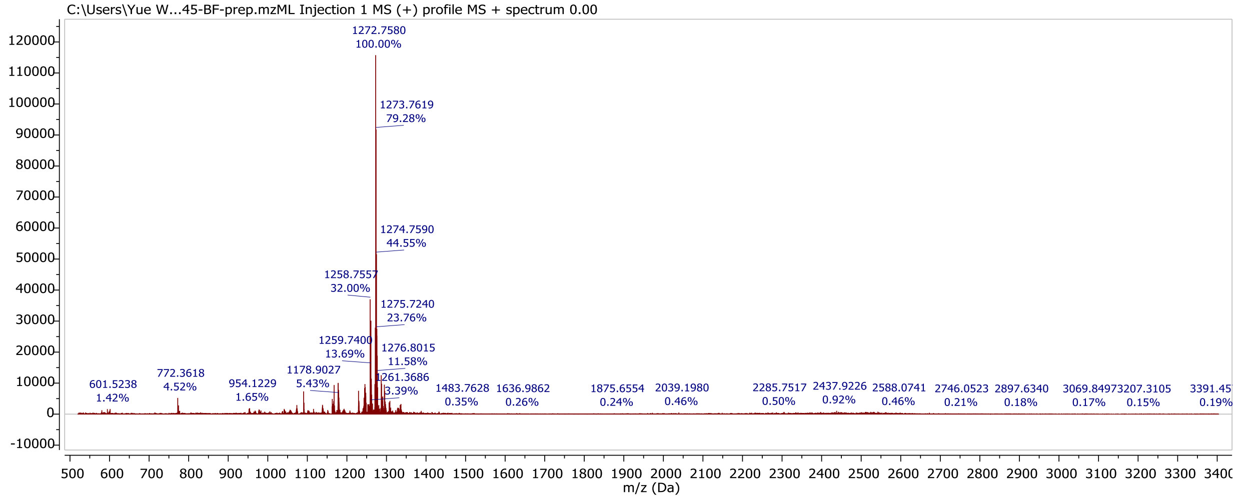

HRMS(MAIDL-TOF) of **BODIPY$_1$-Pep$_7$**

BODIPY$_1$-Pep$_{10}$: BODIPY$_1$-HN-DRVYIHPF-CONH$_2$

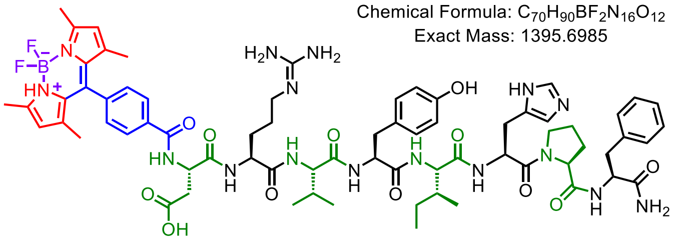

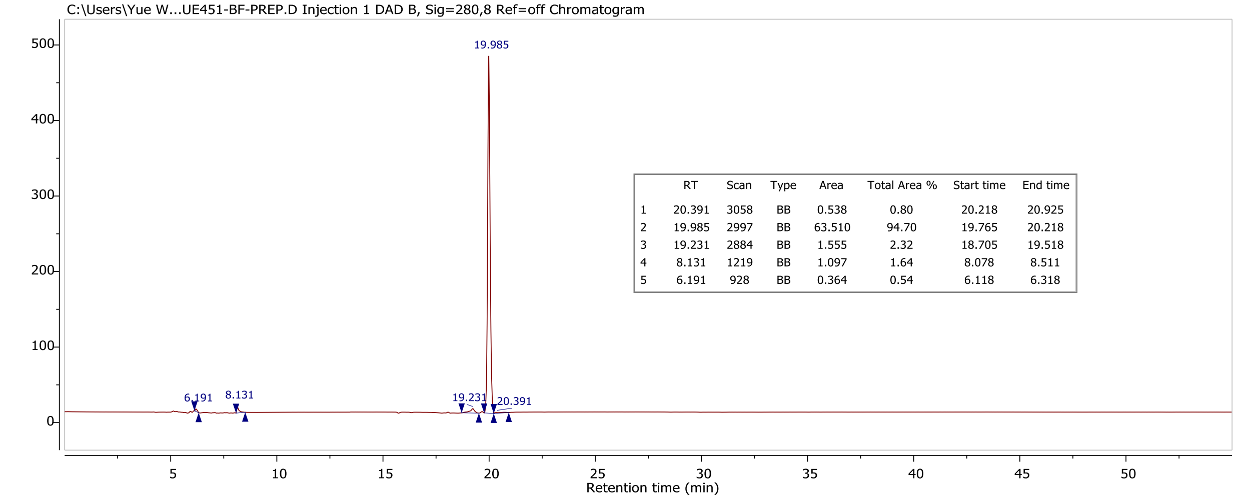

	RT	Scan	Type	Area	Total Area %	Start time	End time
1	20.391	3058	BB	0.538	0.80	20.218	20.925
2	19.985	2997	BB	63.510	94.70	19.765	20.218
3	19.231	2884	BB	1.555	2.32	18.705	19.518
4	8.131	1219	BB	1.097	1.64	8.078	8.511
5	6.191	928	BB	0.364	0.54	6.118	6.318

Analytic HPLC of **BODIPY$_1$-Pep$_{10}$**

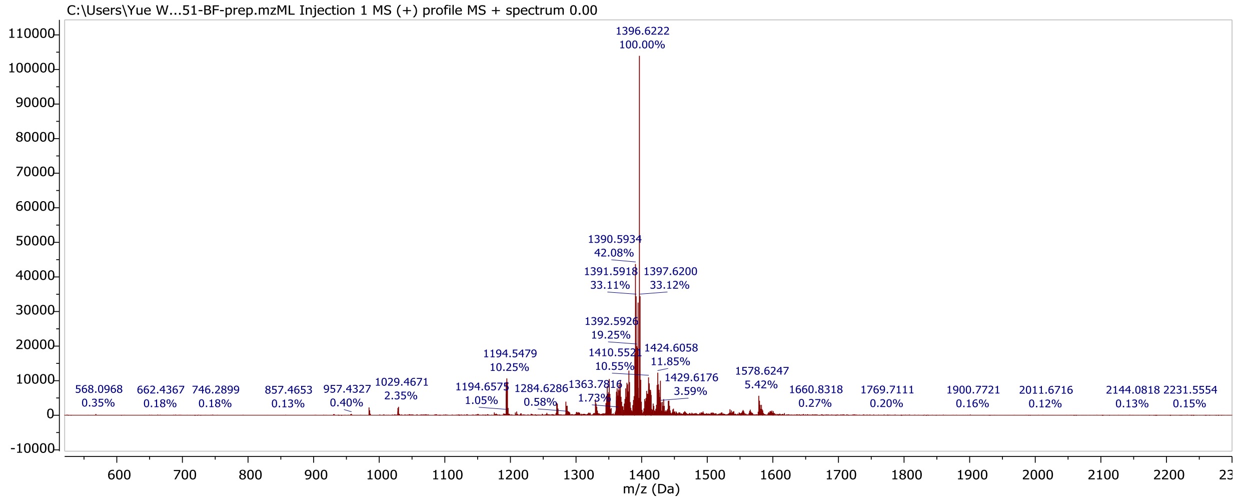

HRMS(MAIDL-TOF) of **BODIPY$_1$-Pep$_{10}$**
